# One-pot four-component synthesis of pyrimidyl and pyrazolyl substituted azulenes by glyoxylation–decarbonylative alkynylation–cyclocondensation sequences

**DOI:** 10.3762/bjoc.7.136

**Published:** 2011-08-26

**Authors:** Charlotte F Gers, Julia Rosellen, Eugen Merkul, Thomas J J Müller

**Affiliations:** 1 Institut für Organische Chemie und Makromolekulare Chemie, Heinrich-Heine-Universität Düsseldorf, Universitätsstr. 1, D-40225 Düsseldorf, Germany

**Keywords:** azulenes, catalysis, decarbonylation, multicomponent reactions, ynones

## Abstract

A novel one-pot four-component synthesis of pyrimidyl- and pyrazolylazulenes through the use of glyoxylation–decarbonylative alkynylation–cyclocondensation sequences starting from azulene or guaiazulene as substrates, gives rise to the formation of the target compounds in moderate to good yields.

## Introduction

Diversity-oriented synthesis has become an important field in organic chemistry, initiated by the increasing demand for new scaffolds for pharmaceuticals and biologically active compounds over the past decades [1–3]. Herein, multicomponent reactions adopt a central position since each component can be varied within a wide range of functionalities and substituents [4–8]. Furthermore, these one-pot processes are highly advantageous because they combine shortened reaction times and resource efficiency with diminished waste production in comparison to traditional multistep syntheses. Thus, they can be considered to be economically and ecologically efficient [9–10].

In particular, multicomponent syntheses of heterocycles initiated by transition metal catalysis received increasing attention in the past decade [11]. As a one-pot synthetic methodology, this novel concept combines the unique reactivity patterns of transition metal catalysis with fundamental organic reactivity, in a sequential or consecutive fashion. Over the years, we have contributed to this concept through Pd/Cu-catalyzed accesses to enones and ynones and the in situ transformation of these intermediates into many classes of heterocycles [12–15]. These novel MCRs nicely correspond with diversity-oriented strategies towards functional organic chromophores [1–2].

**Scheme 1:**
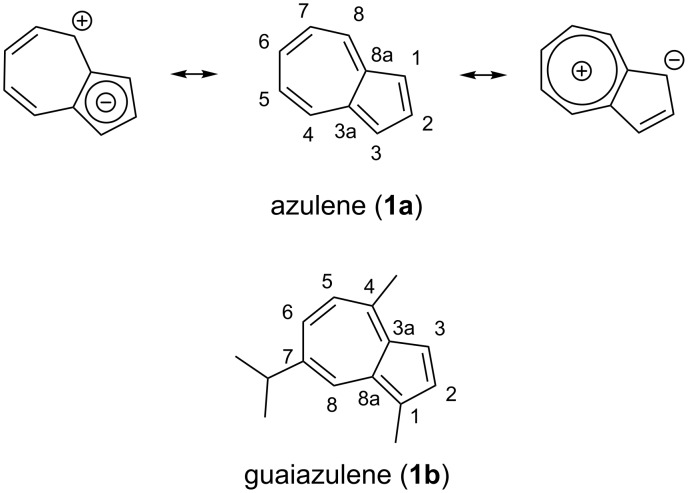
Selected resonance structures of azulene (**1a**) and structure of the sesquiterpene guaiazulene (**1b**).

The striking blue color of azulene (**1a**) (from the Spanish word “azul” = blue) has aroused scientific attention for a long time [16–17]. This prominent appearance results from the electronic transition between the S_0_ and S_1_ state [18], as a consequence of low energy frontier molecular orbital transitions [19]. The bicyclic structure of this nonbenzoid hydrocarbon results from a five–seven ring annulation with a planar, cyclic conjugation of 10 π-electrons. The dipole moment of **1a** at μ = 1.08 D [20] is astoundingly large in comparison to that of naphthalene at μ = 0 D and can be rationalized by a significant contribution of cyclopentadienyl anion/tropylium cation resonance structures (Scheme 1) [19].

Since the elucidation of the structure and the first synthesis of the azulene skeleton by Pfau and Plattner [21–22], its reactivity has been intensively studied [23–26]. The aromatic system is susceptible to nucleophilic addition in the 4-, 6- and 8-positions [23], whereas electrophilic aromatic substitution, such as Friedel–Crafts-type reactions, generally occurs in the 1-position [24]. Interestingly, the azulene motif is also found in terpenoids [27–28]. Guaiazulene (**1b**) (Scheme 1), a commonly known derivative of azulene (**1a**), is a naturally occurring sesquiterpene [29]. Guaiazulene (**1b**) has found entry in a wide range of cosmetic formulations [30]. In addition, numerous azulene derivatives display appealing properties for material [31–33] and pharmaceutical sciences [34–38]. Furthermore, the use of the azulene moiety as part of a protecting group chromophore in carbohydrate chemistry has recently been reported [39].

*N-*Heteroaryl-substituted azulenes can be accessed by stoichiometric [40–41] as well as Pd-catalyzed cross-coupling processes [42–44]. However, these methods have only delivered a narrow range of derivatives. Prior to application in Pd-catalyzed processes, azulenes must be functionalized, either by halogenation or borylation, and some of these derivatives were found to be quite unstable [45–46]. To the best of our knowledge, no diversity-oriented multicomponent syntheses of azulenyl heterocycles have been reported so far. Here, we report the development of one-pot four-component syntheses toward pyrimidyl- and pyrazolylazulenes.

## Results and Discussion

**Scheme 2:**
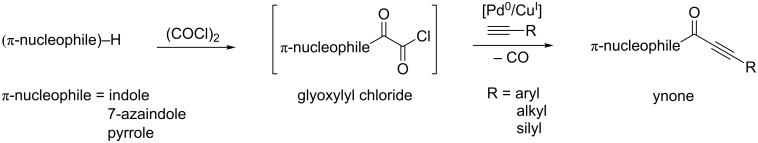
Synthesis of ynones by glyoxylation–decarbonylative Sonogashira coupling.

Recently, we reported a three-component synthesis leading to the formation of ynones by a conceptually novel glyoxylation–decarbonylative Sonogashira coupling sequence (Scheme 2) [47]. The Lewis acid free glyoxylation of electron rich *N-*heterocycles, such as indoles and pyrroles, leads to the formation of glyoxylyl chlorides, which can be reacted without isolation by decarbonylative Sonogashira coupling to form the desired ynones. So far, only one example of the synthesis of azulenylynones has been described [48].

**Scheme 3:**
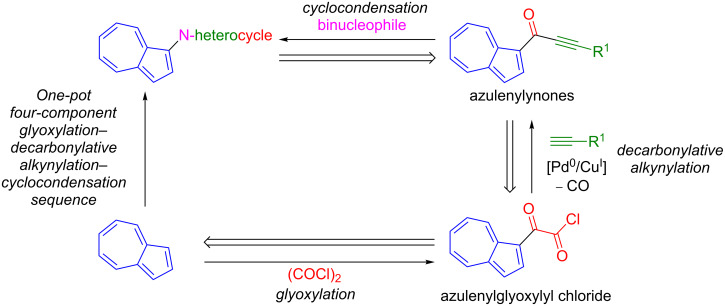
Retrosynthetic analysis of *N*-heterocyclic substituted azulenes by a one-pot four-component approach.

Our retrosynthetic analysis (Scheme 3) suggests that a wide range of *N-*heterocycle-substituted azulenes should be accessible through Michael addition–cyclocondensation of azulenylynones with binucleophiles. Azulenylynones in turn could be simply disconnected by our glyoxylation–decarbonylative alkynylation transform [47] back to azulenes.

Previously, glyoxylation of azulene (**1a**), with oxalyl chloride in 1-position was reported to be essentially complete within 5 min [39]. Oxalyl bromide could be equally used as a glyoxylating agent [49–50]. Likewise, the glyoxylation of **1b** has been reported to proceed in 3-position with both reagents, yet with lower reactivity, and its conversion was found to be incomplete even after 2 h. In addition, the formation of side products [51] and decarbonylation [52] was observed, presumably caused by the steric hindrance of the methyl group in 4-position.

Encouraged by our smooth glyoxylation–alkynylation sequences with a variety of unfunctionalized π-nucleophiles, such as pyrazoles, thiophenes, furans, and even the hydrocarbon azulene (**1a**) [53], we decided to perform optimization studies of the glyoxylation–decarbonylative alkynylation with guaiazulene (**1b**), a commercially available and inexpensive azulene derivative, and 1-hexyne (**2b**) as model substrates (Table 1) (for experimental details, see Supporting Information File 1).

**Table 1:** Optimization studies for the synthesis of ynone **3k**.^a^

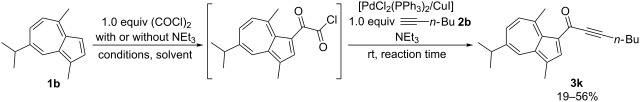									

	Glyoxylation step				Sonogashira coupling step				
				
Entry	NEt_3_ [equiv]	*T* [°C]	*t* [h]		PdCl_2_(PPh_3_)_2_ [mol %]	CuI [mol %]	NEt_3_ [equiv]	*t* [h]	Yield **3k** [%]^b^

1	1.0	0 °C to rt	4		1	1	1.0	1	40
2	-	0 °C to rt	4		1	1	2.0	1	43
3	1.0	0 °C to rt	24		1	1	1.0	1	36
4	1.0	0 °C to rt	2		1	1	1.0	1	25
5	1.0	0 °C to rt	4		1	1	1.0	2	41
6^c^	-	rt to 50 °C	4		1	1	2.0	1	19
**7**	-	**0 °C to rt**	**4**		**2**	**2**	**2.0**	**1**	**56**
8^c^	-	rt	4		2	2	2.0	1	55

^a^The reactions were performed on a 2.00 mmol scale in 10 mL of THF as a solvent (*c* (**1b**) = 0.2 M); ^b^isolated yield; ^c^1,4-Dioxane was used as a solvent (*c* (**1b**) = 0.2 M).

**Scheme 4:**
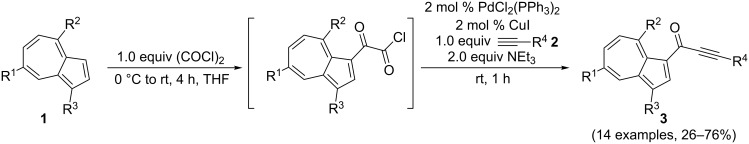
Three-component synthesis of azulenyl- and guaiazulenylynones **3** by glyoxylation–decarbonylative Sonogashira coupling sequence.

Initially, the optimized conditions for the glyoxylation–decarbonylative alkynylation of indoles were applied [47], except for the addition of one equivalent of triethylamine in the glyoxylation step for scavenging the generated hydrogen chloride (Table 1, entry 1). However, the use of the amine base in the first step was unsatisfactory (Table 1, entry 2). Prolonged reaction times in the first step did not affect the yield. According to monitoring by TLC, glyoxylation of guaiazulene (**1b**) was incomplete even after 24 h reaction time (Table 1, entry 3). Shorter reaction times in the first step caused a substantial decrease of the yield (Table 1, entry 4), whereas longer reaction times in the Sonogashira coupling had no effect on the yield (Table 1, entry 5). Rising the reaction temperature of the glyoxylation step to 50 °C considerably diminished the yield (Table 1, entry 6). However, doubling the catalyst loading furnished significantly higher yields (Table 1, entry 7). 1,4-Dioxane was equally well employed as a solvent (Table 1, entry 8). From this optimization study, the conditions of entry 7 (Table 1) were considered to be optimal and were applied in the three-component synthesis of the azulenylynones **3** (Scheme 4, Table 2) (for experimental details, see Supporting Information File 1). Their structures were unambiguously supported by NMR spectroscopy, mass spectrometry, and combustion analysis.

**Table 2:** Three-component synthesis of azulenyl- and guaiazulenylynones **3**.^a^

Entry	Azulene **1**	Alkyne **2**	Azulenylynone **3**	[%]^b^

1	**1a** (R^1^ = R^2^ = R^3^ = H)	**2a** (R^4^ = Ph)	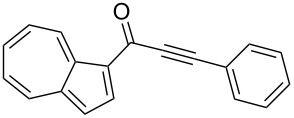 **3a**	65^c^
2	**1a**	**2b** (R^4^ = *n*-Bu)	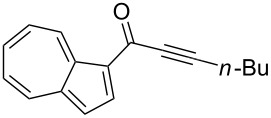 **3b**	66^c^
3	**1b** (R^1^ = iPr, R^2^ = R^3^ = Me)	**2a**	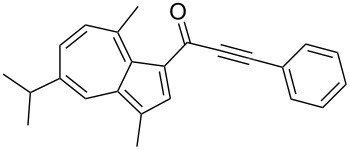 **3c**	55
4	**1b**	**2c** (R^4^ = *p*-tolyl)	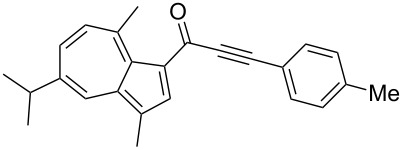 **3d**	57
5	**1b**	**2d** (R^4^ = *p*-CNC_6_H_4_)	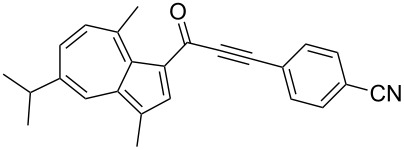 **3e**	60
6	**1b**	**2e** (R^4^ = *p*-NO_2_C_6_H_4_)	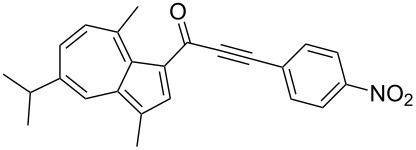 **3f**	76
7	**1b**	**2f** (R^4^ = *m*-FC_6_H_4_)	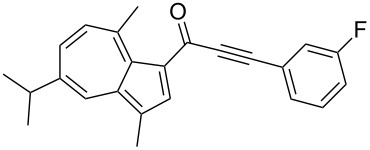 **3g**	51
8	**1b**	**2g** (R^4^ = 3,5-(MeO)_2_C_6_H_3_)	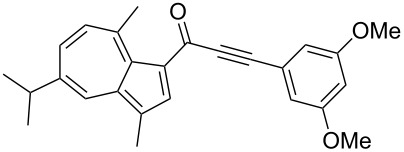 **3h**	47
9	**1b**	**2h** (R^4^ = 2-C_4_H_3_S)	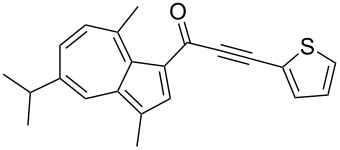 **3i**	55
10	**1b**	**2i** (R^4^ = 3-pyridyl)	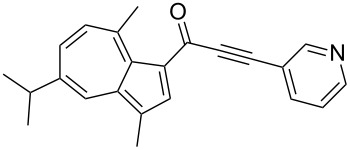 **3j**	31
11	**1b**	**2b**	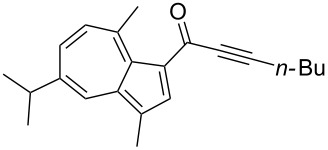 **3k**	56
12	**1b**	**2j** (R^4^ = cyclopropyl)	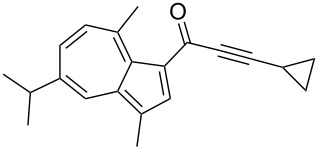 **3l**	42
13	**1b**	**2k** (R^4^ = CH(OEt)_2_)	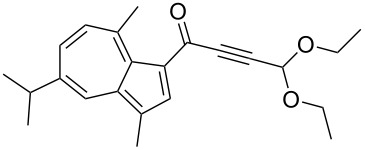 **3m**	30
14	**1b**	**2l** (R^4^ = TIPS)	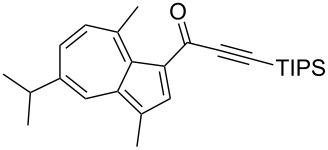 **3n**	26

^a^The reactions were performed on a 2.00 mmol scale in 10 mL of THF as a solvent (*c* (**1**) = 0.2 M); ^b^Isolated and purified compounds; ^c^The reactions were performed on a 1.00 mmol scale in 5 mL THF as a solvent (*c* (**1**) = 0.2 M).

Azulene (**1a**) and guaiazulene (**1b**) were both applied as substrates in the reaction sequence, giving rise to azulenyl- and guaiazulenylynones **3**. The azulenyl derivatives **3a** and **3b** were obtained in higher yields compared to the guaiazulenylynones **3c**–**n**. A variety of substituted arylacetylenes were utilized in the reaction sequence. Electron neutral (Table 2, entries 1 and 3), electron withdrawing (Table 2, entries 5–7), and electron donating (Table 2, entries 4 and 8) substituents were equally well tolerated. In addition, heteroaryl-substituted acetylenes (Table 2, entries 9 and 10) as well as simple aliphatic acetylenes (Table 2, entries 2, 11, and 12) were successfully employed. Finally, propargylaldehyde diethylacetal (Table 2, entry 13) and TIPS-protected acetylene (Table 2, entry 14) also participated in the sequence, although relatively low yields were achieved.

**Scheme 5:**
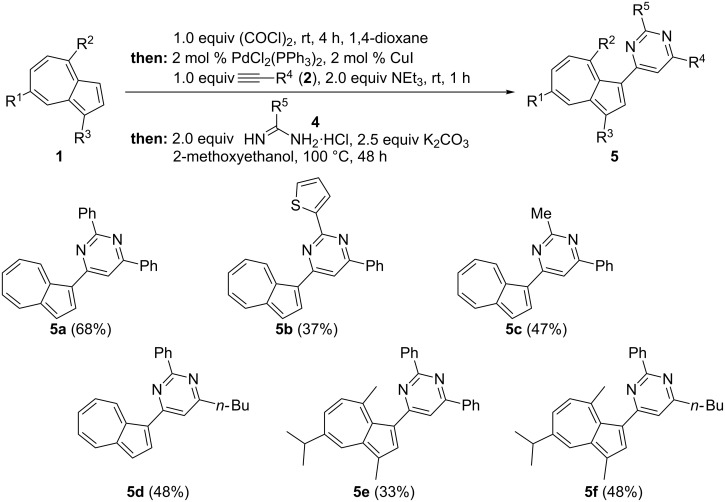
Four-component synthesis of pyrimidylazulenes **5** by glyoxylation–decarbonylative Sonogashira coupling–cyclocondensation sequence (yields refer to isolated and purified compounds).

With this versatile three-component synthesis of azulenylynones in hand, the stage was set to expand the sequence to a four-component access to pyrimidyl- and pyrazolyl-substituted azulenes. Hence, the conditions for the terminating Michael addition–cyclocondensation step, adopted from a recent work [54], were only slightly adjusted as a consequence of the lower electrophilicity of azulenylynones in comparison to aryl- and heteroaryl-substituted ynones that we have previously synthesized. Therefore, upon the subsequent reaction of the azulenes **1** with oxalyl chloride, followed by Pd/Cu-catalyzed decarbonylative alkynylation with terminal alkynes **2**, and finally by cyclocondensation of the ynone intermediates with substituted amidine hydrochlorides **4**, pyrimidylazulenes **5** were obtained in moderate to good yields in a one-pot fashion (Scheme 5) (for experimental details, see Supporting Information File 1).

The diversity-oriented nature of this four-component approach to pyrimidylazulenes **5** is underlined by flexible variation of the azulenyl, the alkynyl, and the amidinyl substrates. In particular, the amidine component **4** leads to the formation of aryl (compounds **5a**, **5d**–**5f**), heteroaryl (compound **5b**) or alkyl (compound **5c**) pyrimidylazulene derivatives.

**Scheme 6:**
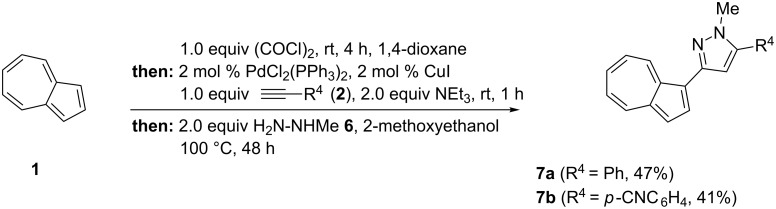
Four-component synthesis of pyrazolylazulenes **7** by glyoxylation–decarbonylative Sonogashira coupling–cyclocondensation sequence (yields refer to isolated and purified compounds).

Likewise, pyrazolylazulenes were obtained in the course of a consecutive glyoxylation–decarbonylative Sonogashira coupling, followed by a cyclocondensation with methylhydrazine (**6**) to furnish two *N*-methylpyrazoles **7** in moderate yields (Scheme 6) (for experimental details, see Supporting Information File 1).

Attempts to employ phenylhydrazine, *N*-Boc-hydrazine, and hydrazine hydrate under standard conditions were met with failure. Based upon previous syntheses of *N*-methylpyrazoles from ynones and methylhydrazine [55–56] and the appearance of a single set of resonances in the proton and carbon NMR spectra, it is obvious that only a single regioisomer was formed. Although the synthesis of similarly substituted pyrazolylazulenes has already been described [57], our one-pot four-component approach utilizes readily available starting materials as well as a simple catalyst system. In addition, it avoids tedious multiple workup and purification operations.

## Conclusion

In conclusion, we have developed a one-pot four-component process for the synthesis of novel pyrimidyl- and pyrazolylazulenes. A wide range of substituents can be introduced by this modular approach to *N*-heterocyclic azulene derivatives. The key step of this diversity-oriented synthesis is the generation of azulenylynones by the glyoxylation–decarbonylative alkynylation sequence with azulene or guaiazulene as substrates. Undoubtedly, this novel four-component approach to heterocyclic derivatives of azulene is well suited for the development of functional chromophores with extended π-conjugation.

## Supporting Information

File 1 Experimental procedures, spectroscopic and analytical data, and copies of NMR spectra of compounds **3**, **5**, and **7**.

## References

[1] Müller T J J, D’Souza D M (2008). Pure Appl Chem.

[2] Müller T J J, Müller T J J, Bunz U H F (2007). Functional Organic Materials. Synthesis, Strategies, and Applications.

[3] Burke M D, Schreiber S L (2004). Angew Chem, Int Ed.

[4] Sunderhaus J D, Martin S F (2009). Chem–Eur J.

[5] Isambert N, Lavilla R (2008). Chem–Eur J.

[6] Orru R V A, de Greef M (2003). Synthesis.

[7] Touré B B, Hall D G (2009). Chem Rev.

[8] Bonne D, Coquerel Y, Constantieux T, Rodriguez J (2010). Tetrahedron: Asymmetry.

[9] Tietze L F (1996). Chem Rev.

[10] Coquerel Y, Boddaert T, Presset M, Mailhol D, Rodriguez J, Pignataro B (2010). Multiple Bond-Forming Transformations: The Key Concept toward Eco-Compatible Synthetic Organic Chemistry. Ideas in Chemistry and Molecular Sciences: Advances in Synthetic Chemistry.

[11] D’Souza D M, Müller T J J (2007). Chem Soc Rev.

[12] Müller T J J, Orru R V A, Ruijter E (2010). Palladium-Copper Catalyzed Alkyne Activation as an Entry to Multicomponent Syntheses of Heterocycles. Synthesis of Heterocycles via Multicomponent Reactions II.

[13] Willy B, Müller T J J (2009). Curr Org Chem.

[14] Willy B, Müller T J J (2008). ARKIVOC.

[15] Müller T J J, Attanasi O, Spinelli D (2006). Multi-component syntheses of heterocycles by virtue of palladium catalyzed generation of alkynones and chalcones. Targets in Heterocyclic Systems.

[16] Hansen H J (1996). Chimia.

[17] Hansen H J (1997). Chimia.

[18] Liu R S H (2002). J Chem Educ.

[19] Zeller K-P (1985). Houben-Weyl.

[20] Anderson A G, Steckler B M (1959). J Am Chem Soc.

[21] Pfau A S, Plattner P A (1936). Helv Chim Acta.

[22] Plattner P A, Pfau A S (1937). Helv Chim Acta.

[23] Hafner K, Bernhard C, Müller R (1961). Justus Liebigs Ann Chem.

[24] Anderson A G, Nelson J A (1950). J Am Chem Soc.

[25] Hafner K, Moritz K-L (1962). Justus Liebigs Ann Chem.

[26] Kędziorek M, Mayer P, Mayr H (2009). Eur J Org Chem.

[27] Fraga B M (2008). Nat Prod Rep.

[28] Faulkner D J (1995). Nat Prod Rep.

[29] Seo Y, Rho J-R, Geum N, Yoon J B, Shin J (1996). J Nat Prod.

[30] Andersen F A (1999). Int J Toxicol.

[31] Barman S, Furukawa H, Blacque O, Venkatesan K, Yaghi O M, Berke H (2010). Chem Commun.

[32] Wang F, Lai Y-H (2003). Macromolecules.

[33] Zhang X-H, Li C, Wang W-B, Cheng X-X, Wang X-S, Zhang B-W (2007). J Mater Chem.

[34] Ramadan M, Goeters S, Watzer B, Krause E, Lohmann K, Bauer R, Hempel B, Imming P (2006). J Nat Prod.

[35] Jakovlev V, Isaac O, Flaskamp E (1983). Planta Med.

[36] Chen C-H, Lee O, Yao C-N, Chuang M-Y, Chang Y-L, Chang M-H, Wen Y-F, Yang W-H, Ko C-H, Chou N-T (2010). Bioorg Med Chem Lett.

[37] Safayhi H, Sabieraj J, Sailer E-R, Ammon H P T (1994). Planta Med.

[38] Fiori J, Teti G, Gotti R, Mazzotti G, Falconi M (2011). Toxicol in Vitro.

[39] Timmer M S M, Stocker B L, Northcote P T, Burkett B A (2009). Tetrahedron Lett.

[40] Shoji T, Yokoyama R, Ito S, Watanabe M, Toyota K, Yasunami M, Morita N (2007). Tetrahedron Lett.

[41] Shoji T, Okada K, Ito S, Toyota K, Morita N (2010). Tetrahedron Lett.

[42] Kurotobi K, Tabata H, Miyauchi M, Murafuji T, Sugihara Y (2002). Synthesis.

[43] Wakabayashi S, Kato Y, Mochizuki K, Suzuki R, Matsumoto M, Sugihara Y, Shimizu M (2007). J Org Chem.

[44] Wakabayashi S, Uriu R, Asakura T, Akamatsu C, Sugihara Y (2008). Heterocycles.

[45] Ito S, Kubo T, Morita N, Matsui Y, Watanabe T, Ohta A, Fujimori K, Murafuji T, Sugihara Y, Tajiri A (2004). Tetrahedron Lett.

[46] Higashi J, Shoji T, Ito S, Toyota K, Yasunami M, Morita N (2008). Eur J Org Chem.

[47] Merkul E, Oeser T, Müller T J J (2009). Chem–Eur J.

[48] Hafner K, Bangert K-F (1961). Justus Liebigs Ann Chem.

[49] Treibs W, Orttmann H (1958). Naturwissenschaften.

[50] Ito S, Okujima T, Kikuchi S, Shoji T, Morita N, Asao T, Ikoma T, Tero-Kubota S, Kawakami J, Tajiri A (2008). J Org Chem.

[51] Reid D H, Stafford W H, Stafford W L (1958). J Chem Soc.

[52] Treibs W (1959). Chem Ber.

[53] Merkul E, Dohe J, Gers C, Rominger F, Müller T J J (2011). Angew Chem, Int Ed.

[54] Boersch C, Merkul E, Müller T J J (2011). Angew Chemie, Int Ed.

[55] Willy B, Müller T J J (2011). Org Lett.

[56] Willy B, Müller T J J (2008). Eur J Org Chem.

[57] Wang D-L, Deng J-J, Xu J, Imafuku K (2007). Heterocycles.

