# Supplementary material for: One-pot four-component synthesis of pyrimidyl and pyrazolyl substituted azulenes by glyoxylation–decarbonylative alkynylation–cyclocondensation sequences
Source: Beilstein J Org Chem. 2011 Aug 26;7:1173–81. doi: 10.3762/bjoc.7.136 (PMC3168959; doi:10.3762/bjoc.7.136)
Supplement: File 1 — Experimental procedures, spectroscopic and analytical data, and copies of NMR spectra of compounds 3, 5, and 7. [file Beilstein_J_Org_Chem-07-1173-s001.pdf]

Supporting Information

for

One-pot four-component synthesis of pyrimidyl and pyrazolyl substituted azulenes by glyoxylation–decarbonylative alkynylation–cyclocondensation sequences

Charlotte F. Gers, Julia Rosellen, Eugen Merkul, and Thomas J. J. Müller*

Address: Institut für Organische Chemie und Makromolekulare Chemie, Heinrich-Heine-Universität Düsseldorf, Universitätsstr. 1, D-40225 Düsseldorf, Germany.

Email: Charlotte F. Gers - Charlotte.Gers@uni-duesseldorf.de, Julia Rosellen - Julia.Rosellen@uni-duesseldorf.de, Eugen Merkul - E.Merkul@uni-duesseldorf.de, Thomas J. J. Müller* - ThomasJJ.Mueller@uni-duesseldorf.de

* Corresponding author

Experimental procedures, spectroscopic and analytical data, and copies of NMR spectra of compounds 3, 5, and 7.

Contents

1 General considerations 1

2 Preparation of ynones via glyoxylation–decarbonylative Sonogashira coupling sequence 2

2.1 General procedure for the synthesis of ynones 2

2.2 Spectroscopic data of ynones **3** 6

2.2.1 1-(Azulen-1-yl)-3-phenylprop-2-yn-1-one (**3a**) 6

2.2.2 1-(Azulen-1-yl)hept-2-yn-1-one (**3b**) 7

2.2.3 1-[3,8-Dimethyl-5-(propan-2-yl)azulen-1-yl]-3-phenylprop-2-yn-1-one (**3c**) 8

2.2.4 1-[3,8-Dimethyl-5-(propan-2-yl)azulen-1-yl]-3-(4-methylphenyl)prop-2-yn-1-one (**3d**) 9

2.2.5 4-{3-[3,8-Dimethyl-5-(propan-2-yl)azulen-1-yl]-3-oxoprop-1-yn-1-yl}benzonitrile (**3e**) 10

2.2.6 1-[3,8-Dimethyl-5-(propan-2-yl)azulen-1-yl]-3-(4-nitrophenyl)prop-2-yn-1-one (**3f**) 11

2.2.7 1-[3,8-Dimethyl-5-(propan-2-yl)azulen-1-yl]-3-(3-fluorophenyl)prop-2-yn-1-one (**3g**) 12

2.2.8 3-(3,5-Dimethoxyphenyl)-1-[3,8-dimethyl-5-(propan-2-yl)azulen-1- yl]prop-2-yn-1-one (**3h**) 13

2.2.9 1-[3,8-Dimethyl-5-(propan-2-yl)azulen-1-yl]-3-(thiophen-2-yl)prop-2-yn-1-one (**3i**) 14

2.2.10 1-[3,8-Dimethyl-5-(propan-2-yl)azulen-1-yl]-3-(pyridin-3-yl)prop-2-yn-1-one (**3j**) 15

2.2.11 1-[3,8-Dimethyl-5-(propan-2-yl)azulen-1-yl]hept-2-yn-1-one (**3k**) 16

2.2.12 3-Cyclopropyl-1-[3,8-dimethyl-5-(propan-2-yl)azulen-1-yl]prop-2-yn-1- one (**3l**) 17

2.2.13 1-[3,8-Dimethyl-5-(propan-2-yl)azulen-1-yl]-4,4-diethoxybut-2-yn-1-one (**3m**) 18

2.2.14 1-[3,8-Dimethyl-5-(propan-2-yl)azulen-1-yl]-3-[tris(propan-2-yl)silyl]prop-2-yn-1-one (**3n**) 19

3 Preparation of pyrimidines **5** 20

3.1 General procedure for the synthesis of pyrimidines **5** 20

3.2 Spectroscopic data of pyrimidines **5**. 23

3.2.1 4-(Azulen-1-yl)-2,6-diphenylpyrimidine (**5a**) 23
3.2.2 4-(Azulen-1-yl)-6-phenyl-2-(thiophen-2-yl)pyrimidine (**5b**) 24
3.2.3 4-(Azulen-1-yl)-2-methyl-6-phenylpyrimidine (**5c**) 25
3.2.4 4-(Azulen-1-yl)-6-butyl-2-phenylpyrimidine (**5d**) 26
3.2.5 4-[3,8-Dimethyl-5-(propan-2-yl)azulen-1-yl]-2,6-diphenyl-pyrimidine (**5e**) 27
3.2.6 4-Butyl-6-[3,8-dimethyl-5-(propan-2-yl)azulen-1-yl]-2-phenyl-pyrimidine (**5f**) 28
4 Preparation of pyrazoles **7** 29
4.1 General procedure for the synthesis of pyrazoles **7** 29
4.2 Spectroscopic data of pyrazoles **7** 31
4.2.1 3-(Azulen-1-yl)-1-methyl-5-phenyl-1*H*-pyrazole (**7a**) 31
4.2.2 4-{3-[3,8-Dimethyl-5-(propan-2-yl)azulen-1-yl]-1-methyl-1*H*-pyrazol-4-yl}benzonitrile(**7b**) 32
5 ^{1}H and ^{13}C NMR spectra of ynones **3** 33
5.1 1-(Azulen-1-yl)-3-phenylprop-2-yn-1-one (**3a**) 33
5.2 1-(Azulen-1-yl)hept-2-yn-1-one (**3b**) 36
5.3 1-[3,8-Dimethyl-5-(propan-2-yl)azulen-1-yl]-3-phenylprop-2-yn-1-one (**3c**) 39
5.4 1-[3,8-Dimethyl-5-(propan-2-yl)azulen-1-yl]-3-(4-methylphenyl)prop-2-yn-1-one (**3d**) 42
5.5 4-{3-[3,8-Dimethyl-5-(propan-2-yl)azulen-1-yl]-3-oxoprop-1-yn-1-yl}benzonitrile (**3e**) 45
5.6 1-[3,8-Dimethyl-5-(propan-2-yl)azulen-1-yl]-3-(4-nitrophenyl)prop-2-yn-1-one (**3f**) 48
5.7 1-[3,8-Dimethyl-5-(propan-2-yl)azulen-1-yl]-3-(3-fluorophenyl)prop-2-yn-1-one (**3g**) 51
5.8 3-(3,5-Dimethoxyphenyl)-1-[3,8-dimethyl-5-(propan-2-yl)azulen-1-yl]prop-2-yn-1-one (**3h**) 54
5.9 1-[3,8-Dimethyl-5-(propan-2-yl)azulen-1-yl]-3-(thiophen-2-yl)prop-2-yn-1-one (**3i**) 57
5.10 1-[3,8-Dimethyl-5-(propan-2-yl)azulen-1-yl]-3-(pyridin-3-yl)prop-2-yn-1-one (**3j**) 60
5.11 1-[3,8-Dimethyl-5-(propan-2-yl)azulen-1-yl]hept-2-yn-1-one (**3k**) 63

5.12 3-Cyclopropyl-1-[3,8-dimethyl-5-(propan-2-yl)azulen-1-yl]prop-2-yn-1-one (**3l**) ... 66
5.13 1-[3,8-Dimethyl-5-(propan-2-yl)azulen-1-yl]-4,4-diethoxybut-2-yn-1-one (**3m**) ... 69
5.14 1-[3,8-Dimethyl-5-(propan-2-yl)azulen-1-yl]-3-[tris(propan-2-yl)silyl]prop-2-yn-1-one (**3n**) ... 72
6 ^{1}H and ^{13}C NMR spectra of pyrimidines **5** ... 75
6.1 4-(Azulen-1-yl)-2,6-diphenylpyrimidine (**5a**) ... 75
6.2 4-(Azulen-1-yl)-6-phenyl-2-(thiophen-2-yl)pyrimidine (**5b**) ... 78
6.3 4-(Azulen-1-yl)-2-methyl-6-phenylpyrimidine (**5c**) ... 81
6.4 4-(Azulen-1-yl)-6-butyl-2-phenylpyrimidine (**5d**) ... 84
6.5 4-[3,8-Dimethyl-5-(propan-2-yl)azulen-1-yl]-2,6-diphenyl-pyrimidine (**5e**) ... 87
6.6 4-Butyl-6-[3,8-dimethyl-5-(propan-2-yl)azulen-1-yl]-2-phenyl-pyrimidine (**5f**) ... 90
7 ^{1}H and ^{13}C NMR spectra of pyrazoles **7** ... 93
7.1 3-(Azulen-1-yl)-1-methyl-5-phenyl-1*H*-pyrazole (**7a**) ... 93
7.2 4-{3-[3,8-Dimethyl-5-(propan-2-yl)azulen-1-yl]-1-methyl-1*H*-pyrazol-4-yl}benzonitrile (**7b**) ... 96

1 General considerations

All cross-coupling reactions were carried out in oven-dried Schlenk tubes using septa and syringes under a nitrogen or argon atmosphere. Dry tetrahydrofuran and 1,4-dioxane were supplied by MBraun system MB-SPS-800. Triethylamine was refluxed under an argon atmosphere over sodium ketyl, distilled and stored in a Schlenk flask under argon atmosphere over potassium hydroxide pellets.

Chemicals were either commercially obtained from ABCR GmbH & Co KG, Acros Organics, Alfa Aesar GmbH & Co KG, Fluka, Merck KGaA, Riedel-de Haën, Sigma-Aldrich Co and used as supplied or were already available in the research group.

All products were purified by column chromatography on silica gel 60 (0.015–0.040 mm) from Merck KGaA, Darmstadt using flash technique under a pressure of 2 bar. The crude mixtures were absorbed on Celite® 545 (0.02–0.10 mm) from Merck KGaA Darmstadt before chromatographic purification.

The reaction progress was observed qualitatively by using TLC Silica gel 60 F_{254} aluminium sheets. The spots were detected with UV light at 254 nm and with aqueous potassium permanganate solution.

The ^{1}H-, ^{13}C- and 135-DEPT-spectra were recorded on a Bruker AVIII-300 spectrometer. $CDCl_3$ was used as a solvent. The resonance of $CDCl_3$ was locked as internal standard ($CDCl_3$: ^{1}H δ 7.26, ^{13}C δ 77.0). The multiplicities of signals were abbreviated as follows: s: singlet; d: doublet; dd: doublet of doublets; dt: doublet of triplets, dq: doublet of quartets; ddd: doublet of doublets of doublets; t: triplet; sext: sextet; sept: septet; and m: multiplet. The type of carbon atom was determined on the basis of 135-DEPT NMR spectra.

The EI mass spectra were measured on a Finnigen MAT 200 spectrometer and the GC mass spectra were measured on a Thermo Finnigen Trace DSQ spectrometer.

IR spectra were obtained on Shimadzu IRAffinity. The intensity of the signals is abbreviated as following: s (strong), m (medium), w (weak).

The melting points (uncorrected) were measured on Reichert Thermovar.

Combustion analyses were carried out on Perkin Elmer Series II Analyser 2400 in the microanalytical laboratory of the Institut für Pharmazeutische und Medizinische Chemie at the Heinrich-Heine-Universität Düsseldorf.

2 Preparation of ynones by glyoxylation–decarbonylative Sonogashira coupling sequence

2.1 General procedure for the synthesis of ynones 3

1.00 mmol of azulene (**1a**) (130 mg) or 2.00 mmol of guaiazulene (**1b**) (405 mg) in dry THF (5 mL/mmol) were placed under an argon atmosphere in a screw-cap Schlenk tube, degassed with argon and cooled to 0 °C (water/ice, for 15 min). Then, oxalyl chloride (0.09 mL, 1.00 mmol, 1.00 equiv/0.18 mL, 2.00 mmol, 1.00 equiv) was added dropwise to the reaction mixture at 0 °C. The mixture was warmed to rt (water bath) and stirred for 4 h. Subsequently, $PdCl_2(PPh_3)_2$ (14 mg, 0.02 mmol, 2 mol %/28 mg, 0.04 mmol, 2 mol %), CuI (4 mg, 0.02 mmol, 2 mol %/8 mg, 0.04 mmol, 2 mol %), one equiv of terminal alkyne **2**, and dry triethylamine (0.28 mL, 2.00 mmol, 2.00 equiv/0.56 mL, 4.00 mmol, 2.00 equiv) were successively added to the reaction mixture, and stirring at rt was continued for 1 h. Then, 5 mL/10 mL water was added, the phases were separated and the aqueous phase was extracted with dichloromethane (3 × 5 mL/10 mL, monitored by TLC). The combined organic layers were dried with anhydrous sodium sulfate. After removal of the solvents in vacuo the residue was absorbed onto Celite® and purified by chromatography on silica gel with petroleum ether (boiling range 40–60 °C)/ethyl acetate, to give the ynones **3**.

Table 1: Experimental details for the three-component synthesis of ynones **3**.

Entry	Azulene **1**	Alkyne **2**	Ynone **3**<br>(isolated yield)	Chromatographic purification (eluent), R_f
1	**1a**, 130 mg<br>(1.00 mmol)<br>Alfa Aesar	Phenylacetylene (**2a**)<br>0.11 mL<br>(1.00 mmol)<br>Merck	166 mg<br>(0.65 mmol, 65%)<br>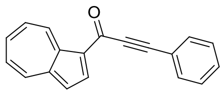<br>**3a**	(PE/EtOAc) = 7:1<br>R_f = 0.19
2	**1a**, 130 mg<br>(1.00 mmol)	Hexyne **2b**<br>0.12 mL<br>(1.00 mmol)<br>Alfa Aesar	155 mg<br>(0.66 mmol, 66%)<br>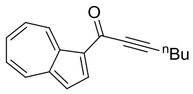<br>**3b**	(PE/EtOAc) = 7:1<br>R_f = 0.31
3	**1b**, 405 mg<br>(2.00 mmol)<br>ABCR	**2a**<br>0.23 mL<br>(2.00 mmol)	358 mg<br>(1.10 mmol, 55%)<br>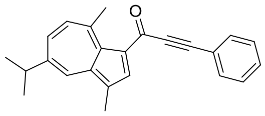<br>**3c**	(PE/EtOAc) = 15:1<br>R_f = 0.15
4	**1b**, 405 mg<br>(2.00 mmol)	1-Ethynyl-4-methylbenzene (**2c**)<br>0.26 mL<br>(2.00 mmol)<br>ABCR	385 mg<br>(1.13 mmol, 57%)<br>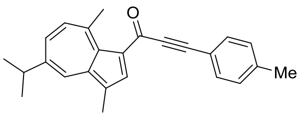<br>**3d**	(PE/EtOAc) = 20:1<br>R_f = 0.10
5	**1b**, 405mg<br>(2.00 mmol)	4-Ethynyl-benzonitrile (**2d**)<br>262 mg<br>(2.00 mmol)<br>Sigma-Aldrich	418 mg<br>(1.19 mmol, 60%)<br>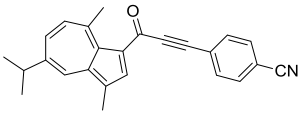<br>**3e**	(PE/EtOAc) = 10:1<br>R_f = 0.06

Table 1 (continued): Experimental details for the three-component synthesis of ynones **3**.

Entry	Azulene **1**	Alkyne **2**	Ynone **3** (isolated yield)	Chromatographic purification (eluent), R_f
6	**1b**, 405 mg (2.00 mmol)	1-Ethynyl-4-nitrobenzene (**2e**) 294 mg (2.00 mmol)	563 mg (1.51 mmol, 76%) 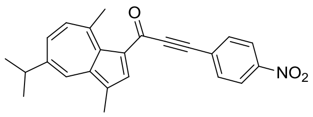 **3f**	(PE/EtOAc) = 5:1 R_f = 0.38
7	**1b**, 405 mg (2.00 mmol)	1-Ethynyl-3-fluorobenzene (**2f**) 0.24 mL (2.00 mmol) Sigma-Aldrich	349 mg (1.01 mmol, 51%) 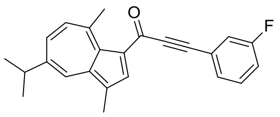 **3g**	(PE/EtOAc) = 15:1 R_f = 0.11
8	**1b**, 405 mg (2.00 mmol)	1-Ethynyl-3,5-dimethoxy-benzene (**2g**) 324 mg (2.00 mmol)	362 mg (0.94 mmol, 47%) 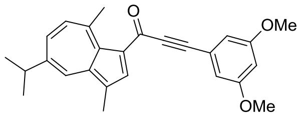 **3h**	(PE/EtOAc) = 8:1 R_f = 0.12
9	**1b**, 405 mg (2.00 mmol)	2-Ethynyl-thiophene **2h** 216 mg (2.00 mmol)	364 mg (1.10 mmol, 55%) 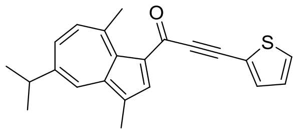 **3i**	(PE/EtOAc) = 15:1 R_f = 0.19
10	**1b**, 405 mg (2.00 mmol)	3-Ethynylpyridine (**2i**) 210 mg (2.00 mmol) Sigma-Aldrich	202 mg (0.62 mmol, 31%) 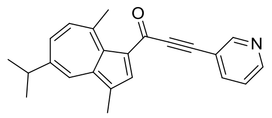 **3j**	(PE/EtOAc) = 3:1 R_f = 0.08

Table 1 (continued): Experimental details for the three-component synthesis of ynones **3**.

Entry	Azulene **1**	Alkyne **2**	Ynone **3** (isolated yield)	Chromatographic purification (eluent), R_f
11	**1b**, 405 mg (2.00 mmol)	**2b** 0.24 mL (2.00 mmol)	344 mg (1.12 mmol, 56%) 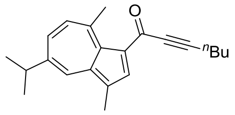 **3k**	(PE/EtOAc) = 20:1 R_f = 0.10
12	**1b**, 405 mg (2.00 mmol)	Ethynyl-cyclopropane (**2j**) 0.17 mL (2.00 mmol) Sigma-Aldrich	245 mg (0.85 mmol, 42%) 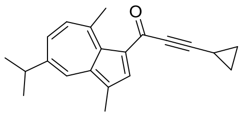 **3l**	(PE/EtOAc) = 10:1 R_f = 0.19
13	**1b**, 405 mg (2.00 mmol)	3,3'-Diethoxyprop-1-yne (**2k**) 0.29 mL (2.00 mmol) ABCR	212 mg (0.60 mmol, 30%) 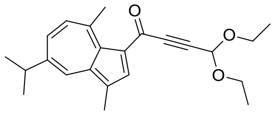 **3m**	(PE/EtOAc) = 10:1 R_f = 0.16
14	**1b**, 405 mg (2.00 mmol)	Ethynyltris(prop-2-yl)silane (**2l**) 0.45 mL (2.00 mmol) Fluka	213 mg (0.52 mmol, 26%) 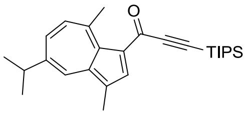 **3n**	(PE/EtOAc) = 20:1 R_f = 0.16

2.2 Spectroscopic data for ynones 3

2.2.1 1-(Azulen-1-yl)-3-phenylprop-2-yn-1-one (3a)

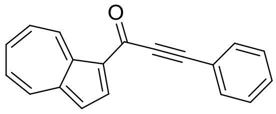

$C_{19}H_{12}O$
256.30

166 mg (0.65 mmol, 65%) as a dark red solid. Mp. 118 °C. ^{1}H NMR ($CDCl_3$, 300 MHz): δ 7.32 (d, *J* = 4.3 Hz, 1 H), 7.38-7.49 (m, 3 H), 7.45 (t, *J* = 9.7 Hz, 1 H), 7.62–7.73 (m, 3 H), 7.86 (t, *J* = 9.8 Hz, 1 H), 8.49 (d, *J* = 9.8 Hz, 1 H), 8.60 (d, *J* = 4.3 Hz, 1 H), 9.91 (d, *J* = 4.3 Hz, 1 H). ^{13}C NMR ($CDCl_3$, 75 MHz): δ 89.1 (C_{quat}), 89.2 (C_{quat}), 118.5 (CH), 121.0 (C_{quat}), 126.3 (C_{quat}), 128.5 (CH), 128.6 (CH), 131.1 (CH), 131.2 (CH), 132.7 (CH), 138.9 (CH), 139.3 (CH), 139.9 (CH), 140.6 (C_{quat}), 143.3 (CH), 146.6 (C_{quat}), 173.3 (C_{quat}). EI + MS (*m/z* (%)): 257 (15), 256 (M^+, 73), 255 ($(M-H)^+$, 12), 229 (20), 228 ($(M-CO)^+$, 100), 227 (26), 226 (60), 202 (11), 155 ($C_{11}H_7O^+$, 7), 127 ($C_{10}H_7^+$,16), 126 (14), 114 (21), 113 (16), 101 ($C_8H_5^+$, 15), 77 ($C_6H_5^+$, 7). IR (solid): $\tilde{\nu}$ 2957 (w) [cm^{-1}], 2924 (w), 2855 (w), 2204 (w), 2180 (w), 1585 (m), 1533 (m), 1490 (m), 1456 (m), 1409 (m), 1392 (m), 1279 (m), 1261 (m), 1198 (m), 1165 (w), 1101 (m), 1070 (w), 1024 (m), 970 (w), 918 (m), 874 (m), 841 (w), 791 (m), 760 (s), 689 (m), 673 (w), 644 (w). Anal. calcd. for $C_{19}H_{12}O$ (256.3): C 89.04, H 4.72. Found: C 88.85, H 4.57.

2.2.2 1-(Azulen-1-yl)hept-2-yn-1-one (3b)

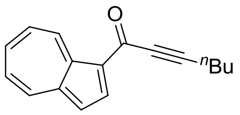

$C_{17}H_{16}O$
236.31

155 mg (0.66 mmol, 66%) as a dark red oil. ^{1}H NMR ($CDCl_3$, 300 MHz): δ 0.98 (t, *J* = 7.2 Hz, 3 H), 1.47–1.60 (m, 2 H), 1.63–1.74 (m, 2 H), 2.15 (t, *J* = 7.0 Hz, 2 H), 7.37 (d, *J* = 4.2 Hz, 1 H), 7.52 (t, *J* = 9.7 Hz, 1 H), 7.63 (t, *J* = 9.6 Hz, 1 H), 7.84 (t, *J* = 9.8 Hz, 1 H), 8.47 (d, *J* = 9.8 Hz, 1 H), 8.49 (d, *J* = 4.1 Hz, 1 H), 9.86 (d, *J* = 9.8 Hz, 1 H). ^{13}C NMR ($CDCl_3$, 75 MHz): δ 13.6 (CH_3), 18.9 (CH_2), 22.1 (CH_2), 30.1 (CH_2), 81.9 (C_{quat}), 92.4 (C_{quat}), 118.3 (CH), 126.3 (C_{quat}), 128.2 (CH), 129.9 (CH), 138.7 (CH), 139.2 (CH), 139.7 (CH), 140.4 (C_{quat}), 143.3 (CH), 146.4 (C_{quat}), 173.9 (C_{quat}). EI + MS (*m/z* (%)): 237 (19), 236 (M^+, 100), 208 ($(M\text{-}CO)^+$, 4), 207 ($(M\text{-}C_2H_5)^+$, 14), 193 ($(M\text{-}C_3H_7)^+$, 17), 179 ($(M\text{-}C_4H_9)^+$, 22), 178 (24), 166 (20), 165 ($(M\text{-}CO\text{-}C_3H_7)^+$, 99), 164 (18), 163 (16), 155 (($C_{11}H_9O^+$, 12) 152 (11), 128 (14), 127 ($C_{10}H_7^+$, 15). IR (oil): $\tilde{\nu}$ 2955 (w) [cm^{-1}], 2930 (w), 2870 (w), 2212 (w), 1587 (m), 1535 (w), 1495 (m), 1456 (m), 1408 (s), 1393 (s), 1321 (m), 1285 (m), 1221 (m), 1209 (m), 1177 (w), 1148 (m), 1036 (w), 1016 (w), 962 (w), 893 (w), 870 (w), 835 (w), 818 (s), 789 (m), 760 (s), 729 (m), 692 (w), 627 (w). Anal. calcd. for $C_{17}H_{16}O$ (236.3): C 86.40, H 6.82. Found: C 86.48, H 6.79.

2.2.3 1-[3,8-Dimethyl-5-(propan-2-yl)azulen-1-yl]-3-phenylprop-2-yn-1-one (3c)

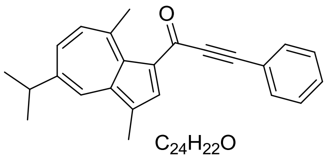

$C_{24}H_{22}O$
326.43

358 mg (1.10 mmol, 55%) as a brown oil. A brown solid was obtained after further purification by suspension in *n*-pentane, followed by sonication in an ultrasound bath, filtration and drying in vacuo overnight. Mp. 111 °C. ^{1}H NMR ($CDCl_3$, 300 MHz): δ 1.39 (d, J = 6.9 Hz, 6 H), 2.61 (s, 3 H), 3.05 (s, 3 H), 3.16 (sept, J = 6.9 Hz, 1 H), 7.37-7.48 (m, 4 H), 7.63 (dd, J = 11.0 Hz, J = 2.1 Hz, 1 H), 7.66-7.72 (m, 2 H), 8.31 (d, J = 2.1 Hz, 1 H), 8.37 (s, 1 H). ^{13}C NMR ($CDCl_3$, 75 MHz): δ 12.9 (CH_3), 24.5 (CH_3), 28.8 (CH_3), 38.1 (CH), 88.6 (C_{quat}), 90.5 (C_{quat}), 121.3 (C_{quat}), 124.8 (C_{quat}), 126.6 (C_{quat}), 128.5 (CH), 129.8 (CH), 132.7 (CH), 133.5 (CH), 134.6 (CH), 136.6 (CH), 137.5 (C_{quat}), 143.4 (C_{quat}), 143.9 (CH), 146.5 (C_{quat}), 150.0 (C_{quat}), 172.4 (C_{quat}). EI + MS (*m/z* (%)): 327 (23), 326 (M^+, 94), 325 ($(M\text{-}H)^+$, 100), 311 ($(M\text{-}CH_3)^+$, 35) 309 (14), 298 ($(M\text{-}CO)^+$, 5), 295 (14), 283 ($(M\text{-}C_3H_7)^+$, 48), 268 (24), 267 (17), 255 (18), 253 ($C_{19}H_9O^+$, 21), 252 (26), 240 (14), 239 (28), 225 ($C_{16}H_{17}O^+$, 4), 191 (10), 189 (12) 165 (27), 149 (19), 141 (19), 134 (10), 131 (10), 129 ($C_9H_5O^+$, 20), 128 (28), 127 (12), 126 (22), 120 (23), 115 (11), 113 (12), 91 (13), 77 ($C_6H_5^+$, 6). IR (solid): $\tilde{\nu}$ 2965 (w) [cm^{-1}], 2922 (w), 2868 (w), 2197 (m), 2139 (w), 1582 (s), 1530 (m), 1487 (w), 1462 (w), 1404 (s), 1377 (w), 1364 (m), 1335 (w), 1306 (w), 1263 (w), 1165 (m), 1103 (w), 1086 (w), 970 (w), 953 (w), 874 (w), 829 (w), 764 (s), 750 (w), 737 (w), 694 (m). Anal. calcd. for $C_{24}H_{22}O$ (326.4): C 88.31, H 6.79. Found: C 88.09, H 6.77.

2.2.4 1-[3,8-Dimethyl-5-(propan-2-yl)azulen-1-yl]-3-(4-methylphenyl)prop-2-yn-1-one (3d)

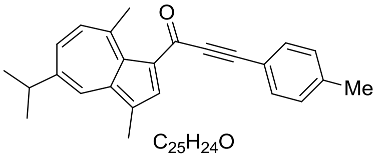

$C_{25}H_{24}O$
340.46

385 mg (1.13 mmol, 57%) as a brown solid. Mp. 122 °C. ^1H NMR ($CDCl_3$, 300 MHz): δ 1.39 (d, J = 6.9 Hz, 6 H), 2.40 (s, 3 H), 2.61 (s, 3 H), 3.04 (s, 3 H), 3.15 (sept, J = 6.9 Hz, 1 H), 7.21 (d, J = 6.9 Hz, 2 H), 7.43 (d, J = 11.0 Hz, 1 H), 7.57–7.64 (m, 3 H), 8.27 (d, J = 2.1 Hz, 1 H), 8.37 (s, 1 H). ^{13}C NMR ($CDCl_3$, 75 MHz): δ 12.9 (CH_3), 21.7 (CH_3), 24.5 (CH_3), 28.8 (CH_3), 38.1 (CH), 89.2 (C_{quat}), 90.3 (C_{quat}), 118.2 (C_{quat}), 124.7 (C_{quat}), 126.7 (C_{quat}), 129.3 (CH), 132.7 (CH), 133.4 (CH), 134.5 (CH), 136.5 (CH), 137.4 (C_{quat}), 140.4 (C_{quat}), 143.3 (C_{quat}), 143.9 (CH), 146.3 (C_{quat}), 149.9 (C_{quat}), 172.7 (C_{quat}). EI + MS (*m/z* (%)): 341 (18), 340 (M^+, 78), 339 ($(M-H)^+$, 100), 325 ($(M-CH_3)^+$, 28) 323 (11), 312 ($(M-CO)^+$, 4), 297 ($(M-C_3H_7)^+$, 30), 282 ($C_{21}H_{14}O^+$, 16), 281 (10), 267 ($C_{20}H_{11}O^+$, 9), 253 (10), 252 (11), 239 (12), 225 ($C_{16}H_{17}O^+$, 3), 165 (13), 149 (10), 143 ($C_{10}H_7O^+$, 4) 58 (11), 57 (10), 43 ($C_3H_7^+$, 37). IR (solid): $\tilde{\nu}$ 2961 (w) [cm^{-1}], 2922 (w), 2866 (w), 2198 (m), 2139 (w), 1587 (s), 1522 (m), 1508 (m), 1458 (w), 1396 (s), 1377 (s), 1364 (s), 1302 (w), 1265 (m), 1244 (w), 1219 (w), 1165 (s), 1126 (w), 1101 (m), 1086 (m), 1057 (w), 1029 (w), 1001 (m), 972 (m), 951 (m), 916 (w), 878 (m), 820 (s), 797 (m), 762 (m), 718 (s), 687 (m), 640 (m). Anal. calcd. for $C_{25}H_{24}O$ (340.5): C 88.20, H 7.11. Found: C 87.89, H 7.31.

2.2.5 4-{3-[3,8-Dimethyl-5-(propan-2-yl)azulen-1-yl]-3-oxoprop-1-yn-1-yl}benzonitrile (3e)

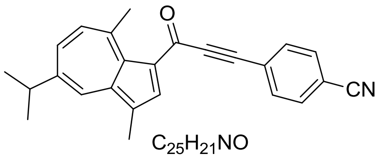

$C_{25}H_{21}NO$
351.44

418 mg (1.19 mmol, 60%) as a brown solid. Mp. 157 °C. ^{1}H NMR ($CDCl_3$, 300 MHz): δ 1.39 (d, *J* = 6.9 Hz, 6 H), 2.60 (s, 3 H), 3.04 (s, 3 H), 3.17 (sept, *J* = 6.9 Hz, 1 H), 7.48 (d, *J* = 11.1 Hz, 1 H), 7.62–7.69 (m, 3 H), 7.73–7.76 (m, 2 H), 8.29 (d, *J* = 2.0 Hz, 1 H), 8.31 (s, 1 H). ^{13}C NMR ($CDCl_3$, 75 MHz): δ 12.9 (CH_3), 24.5 (CH_3), 28.9 (CH_3), 38.1 (CH), 85.5 (C_{quat}), 93.5 (C_{quat}), 113.0 (C_{quat}), 118.2 (C_{quat}), 125.1 (C_{quat}), 126.0 (C_{quat}), 126.3 (C_{quat}), 132.1 (CH), 132.9 (CH), 134.0 (CH), 134.8 (CH), 136.9 (CH), 138.0 (C_{quat}), 143.8 (CH), 143.9 (C_{quat}), 147.3 (C_{quat}), 150.4 (C_{quat}), 171.0 (C_{quat}). EI + MS (*m/z* (%)): 352 (28), 351 (M^+, 100), 350 ($(M-H)^+$, 51), 337 (18), 336 ($(M-CH_3)^+$,63), 334 (15), 323 ($(M-CO)^+$, 8), 309 (14), 308 ($(M-C_3H_7)^+$, 52), 293 ($C_{21}H_{11}NO^+$, 18), 292 (13), 290 (11), 280 ($C_{21}H_{14}N^+$, 20), 278 ($C_{20}H_8NO^+$, 17), 277 (23), 264 (19), 253 (17), 252 (64), 225 ($C_{16}H_{17}O^+$, 6), 191 (12), 189 (10), 166 (10), 165 (31), 154 ($C_{10}H_4NO^+$, 17), 153 (16), 152 (21), 141 (16), 140 (16), 139 (15), 133 (10), 132 (11), 127 (10), 126 ($C_9H_4N^+$, 17), 125 (10), 119 (10). IR (solid): $\tilde{\nu}$ 3088 (w) [cm^{-1}], 2963 (w), 2932 (w), 2895 (w), 2872 (w), 2805 (w), 2722 (w), 2577 (w), 2490 (w), 2409 (w), 2313 (w), 2278 (w), 2228 (w), 2199 (w), 2135 (w), 1830 (w), 1576 (s), 1530 (m), 1497 (m), 1466 (w), 1452 (w), 1393 (s), 1377 (s), 1358 (s), 1296 (m), 1262 (m), 1209 (m), 1177 (m), 1163 (s), 1136 (m), 1099 (m), 1088 (m), 1057 (m), 1026 (w), 999 (m), 972 (m), 959 (m), 912 (m), 878 (m), 868 (m), 839 (s), 822 (s), 800 (m), 787 (m), 760 (m), 731 (m), 687 (s), 640 (m). Anal. calcd. for $C_{25}H_{21}NO$ (351.4): C 85.44, H 6.02, N 3.99. Found: C 85.41, H 6.09, N 3.99.

2.2.6 1-[3,8-Dimethyl-5-(propan-2-yl)azulen-1-yl]-3-(4-nitrophenyl)prop-2-yn-1-one (3f)

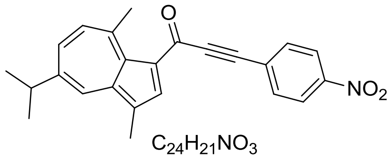

$C_{24}H_{21}NO_3$
371.43

563 mg (1.51 mmol, 76%) as a brown solid. Mp. 189 °C.^{1}H NMR ($CDCl_3$, 300 MHz): δ 1.40 (d, *J* = 6.9 Hz, 6 H), 2.61 (d, *J* = 0.6 Hz, 3 H), 3.05 (s, 3 H), 3.17 (sept, *J* = 6.9 Hz, 1 H), 7.50 (d, *J* = 11.1 Hz, 1 H), 7.67 (dd, *J* = 11.0 Hz, *J* = 2.1 Hz, 1 H), 7.80–7.84 (m, 2 H), 8.25–8.26 (m, 1 H), 8.28–8.30 (m, 2 H), 8.32 (s, 1 H). ^{13}C NMR ($CDCl_3$, 75 MHz): δ 13.0 (CH_3), 24.5 (CH_3), 28.9 (CH_3), 38.2 (CH), 85.1 (C_{quat}), 94.1 (C_{quat}), 123.7 (CH), 125.2 (C_{quat}), 126.0 (C_{quat}), 128.3 (C_{quat}), 133.3 (CH), 134.2 (CH), 134.8 (CH), 137.0 (CH), 138.1 (C_{quat}), 143.9 (CH), 144.1 (C_{quat}), 147.4 (C_{quat}), 147.9 (C_{quat}), 150.5 (C_{quat}), 170.9 (C_{quat}). EI + MS (*m/z* (%)): 372 (24), 371 (M^+, 100), 370 ($(M-H)^+$, 37), 357 (14), 356 ($(M-CH_3)^+$, 56), 354 (11), 343 ($(M-CO)^+$, 6), 328 ($(M-C_3H_7)^+$, 38), 324 (15), 313 ($C_{20}H_{11}NO_3^+$, 7), 310 (11), 309 (14), 293 (15), 292 (64), 282 (22), 267 (11), 266 (14), 265 (20), 263 (15), 262 (17), 254 (10), 253 (17), 252 (28), 250 (12), 240 (10), 240 (11), 239 (27), 226 (15), 225 ($C_{16}H_{17}O^+$, 5), 200 (25), 199 (15), 198 (10), 188 (11), 187 (18), 166 (10), 165 (31), 153 (11), 152 (19), 141 (13), 132 (12), 128 (17), 126 (23), 120 (32), 113 (14), 100 (10). IR (solid): $\tilde{\nu}$ 3109 (w) [cm^{-1}], 2965 (m), 2924 (m), 2855 (w), 2442 (w), 2407 (w), 2355 (w), 2201 (w), 2133 (w), 2097 (w), 1595 (m), 1578 (s), 1532 (m), 1518 (s), 1462 (m), 1393 (s), 1375 (s), 1360 (s), 1339 (s), 1308 (s), 1296 (m), 1260 (m), 1209 (m), 1167 (s), 1136 (m), 1103 (m), 1088 (m), 1057 (m), 1057 (m), 1024 (w), 1001 (m), 974 (m), 959 (m), 912 (m), 880 (m), 855 (s), 820 (m), 766 (s), 746 (s), 731 (m), 683 (s), 658 (m), 640 (m). Anal. calcd. for $C_{24}H_{21}NO_3$ (371.4): C 77.61, H 5.70, N 3.77. Found: C 77.37, H 6.00, N 3.63.

2.2.7 1-[3,8-Dimethyl-5-(propan-2-yl)azulen-1-yl]-3-(3-fluorophenyl)prop-2-yn-1-one (3g)

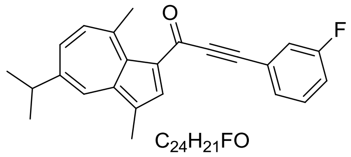

$C_{24}H_{21}FO$
344.42

349 mg (1.01 mmol, 51%) as a brown oil. A brown solid was obtained after further purification by suspension in *n*-pentane, followed sonication in an ultrasound bath, filtration and drying in vacuo overnight. Mp: 71 °C. ^{1}H NMR ($CDCl_3$, 300 MHz): δ 1.39 (d, *J* = 6.9 Hz, 6 H), 2.61 (s, 3 H), 3.04 (s, 3 H), 3.16 (sept, *J* = 6.9 Hz, 1 H), 7.14 (tdd, *J* = 8.4 Hz, *J* = 2.4 Hz, *J* = 1.1 Hz, 1 H), 7.34–7.41 (m, 2 H), 7.44–7.46 (m, 1 H), 7.48 (s, 1 H), 7.64 (dd, *J* = 11.0 Hz, *J* = 2.1 Hz, 1 H), 8.28 (d, *J* = 2.1 Hz, 1 H), 8.35 (s, 1 H). ^{13}C NMR ($CDCl_3$, 75 MHz): δ 12.9 (CH_3), 24.5 (CH_3), 28.8 (CH_3), 38.1 (CH), 86.7 (d, *J* = 3.4 Hz, C_{quat}), 90.1 (C_{quat}), 117.2 (d, *J* = 21.2 Hz, CH), 119.3 (d, *J* = 23.0 Hz, CH), 123.2 (d, *J* = 9.4 Hz, C_{quat}), 124.9 (C_{quat}), 126.4 (C_{quat}), 128.6 (d, *J* = 3.2 Hz, CH), 130.2 (d, *J* = 8.6 Hz, CH), 133.7 (CH), 134.7 (CH), 136.7 (CH), 137.7 (C_{quat}), 143.7 (C_{quat}), 143.9 (CH), 148.4 (d, *J* = 254.6 Hz, C_{quat}), 160.7 (C_{quat}), 164.0 (C_{quat}), 171.8 (C_{quat}). EI + MS (*m/z* (%)): 345 (25), 344 (M^+, 100), 343 ($(M-H)^+$, 51), 329 ($(M-CH_3)^+$, 56), 327 (15), 316 ($(M-CO)^+$, 8), 302 (12), 301 ($(M-C_3H_7)^+$, 53), 286 ($C_{20}H_{11}FO^+$, 24), 285 (17), 283 (12), 273 (20), 271 ($C_{19}H_8FO^+$, 19), 270 (25), 257 (25), 225 ($C_{16}H_{17}O^+$, 3), 191 (13), 165 (33), 152 (20), 147 ($C_9H_4FO^+$, 18), 141 (18), 137 (18), 135 (14), 129 (13), 128 (14), 126 (13), 119 ($M-C_{16}H_{17}O^+$, 8), 115 (10), 109 (12). IR (solid): $\tilde{\nu}$ 3065 (w) [cm^{-1}], 2959 (w), 2926 (w), 2866 (w), 2725 (w), 2199 (w), 1580 (s), 1485 (m), 1429 (m), 1395 (s), 1360 (s), 1337 (m), 1283 (m), 1263 (m), 1184 (s), 1163 (m), 1148 (m), 1126 (w), 1088 (m), 1057 (w), 1024 (w), 999 (m), 982 (m), 959 (w), 936 (w), 920 (w), 866 (w), 839 (w), 783 (m), 760 (m), 721 (s), 685 (m), 642 (m), 633 (m). Anal. calcd. for $C_{24}H_{21}FO$ (344.4): C 83.69, H 6.15. Found: C 83.50, H 6.31.

2.2.8 3-(3,5-Dimethoxyphenyl)-1-[3,8-dimethyl-5-(propan-2-yl)azulen-1-yl]prop-2-yn-1-one (3h)

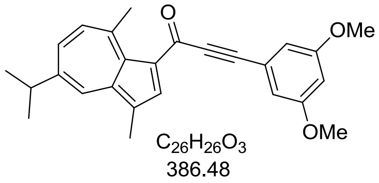

362 mg (0.94 mmol, 47%) as a brown oil. A brown solid was obtained after further purification by suspension in *n*-pentane, followed by sonication in an ultrasound bath, filtration and drying in vacuo overnight. Mp: 82 °C. ^{1}H NMR ($CDCl_3$, 300 MHz): δ 1.39 (d, *J* = 6.9 Hz, 6 H), 2.61 (d, *J* = 0.6 Hz, 3 H), 3.04 (s, 3 H), 3.16 (sept, *J* = 6.9 Hz, 1 H), 3.82 (s, 6 H), 6.55 (t, *J* = 2.3 Hz, 1 H), 6.83 (d, *J* = 2.3 Hz, 2 H), 7.44 (d, *J* = 11.0 Hz, 1 H), 7.63 (dd, *J* = 11.0 Hz, *J* = 2.1 Hz, 1 H), 8.28 (d, *J* = 2.1 Hz, 1 H), 8.35 (s, 1 H). ^{13}C NMR ($CDCl_3$, 75 MHz): δ 12.9 (CH_3), 24.5 (CH_3), 28.8 (CH_3), 38.1 (CH), 55.5 (CH_3), 88.6 (C_{quat}), 89.9 (C_{quat}), 103.4 (CH), 110.3 (CH), 122.6 (C_{quat}), 124.8 (C_{quat}), 126.6 (C_{quat}), 133.5 (CH), 134.6 (CH), 136.6 (CH), 137.6 (C_{quat}), 143.5 (C_{quat}), 143.9 (CH), 146.6 (C_{quat}), 150.0 (C_{quat}), 160.6 (C_{quat}), 172.3 (C_{quat}). EI + MS (*m/z* (%)): 387 (25), 386 (M^+, 100), 385 ($(M-H)^+$, 26), 372 (10), 371 ($(M-CH_3)^+$, 35), 369 (11), 358 ($(M-CO)^+$, 4), 356 ($C_{24}H_{22}O_3^+$, 11), 355 ($(M-OCH_3)^+$, 24), 343 ($(M-C_3H_7)^+$, 27), 339 (10), 328 ($C_{22}H_{16}O_3^+$, 10), 325 (10), 313 ($C_{21}H_{13}O_3^+$, 10), 285 (10), 239 (10), 235 (16), 226 (10), 225 ($C_{16}H_{17}O^+$, 4), 189 (10), 179 (16), 165 (24), 152 (11), 141 (10), 126 (14), 119 (21), 113 (13). IR (solid): $\tilde{\nu}$ 2999 (w) [cm^{-1}], 2959 (w), 2928 (w), 2839 (w), 2193 (w), 1585 (s), 1531 (m), 1454 (m), 1398 (s), 1362 (s), 1335 (m), 1302 (m), 1252 (w), 1202 (s), 1180 (s), 1153 (s), 1088 (m), 1055 (s), 1026 (w), 1007 (m), 989 (w), 939 (w), 926 (m), 845 (m), 824 (s), 758 (m), 735 (w), 679 (m), 660 (s), 640 (m). Anal. calcd. for $C_{26}H_{26}O_3$ (386.5): C 80.80, H 6.78. Found: C 80.54, H 6.80.

2.2.9 1-[3,8-Dimethyl-5-(propan-2-yl)azulen-1-yl]-3-(thiophen-2-yl)prop-2-yn-1-one (3i)

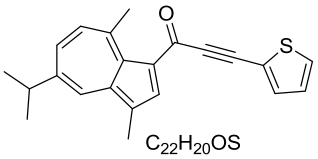

$C_{22}H_{20}OS$
332.46

364 mg (1.10 mmol, 55%) as a brown solid. Mp. 95 °C. ^{1}H NMR ($CDCl_3$, 300 MHz): δ 1.39 (d, J = 6.9 Hz, 6 H), 2.16 (s, 3 H), 3.04 (s, 3 H), 3.15 (sept, J = 6.9 Hz, 1 H), 7.08 (dd, J = 5.1 Hz, J = 3.7 Hz, 1 H), 7.42–7.46 (m, 2 H), 7.52 (dd, J = 3.7 Hz, J = 1.1 Hz, 1 H), 7.62 (dd, J = 11.0 Hz, J = 2.1 Hz, 1 H), 8.28 (d, J = 2.1 Hz, 1 H), 8.31 (s, 1 H). ^{13}C NMR ($CDCl_3$, 75 MHz): δ 12.9 (CH_3), 24.5 (CH_3), 28.8 (CH_3), 38.1 (CH), 82.4 (C_{quat}), 94.8 (C_{quat}), 121.2 (C_{quat}), 124.9 (C_{quat}), 126.3 (C_{quat}), 127.5 (CH), 130.1 (CH), 133.5 (CH), 134.6 (CH), 135.3 (CH), 136.6 (CH), 137.5 (C_{quat}), 143.4 (C_{quat}), 143.7 (CH), 146.5 (C_{quat}), 150.0 (C_{quat}), 172.0 (C_{quat}). EI + MS (*m/z* (%)): 333 (26), 332 (M^+, 100), 331 ($(M-H)^+$, 16), 318 (14), 317 ($(M-CH_3)^+$, 57), 304 ($(M-CO)^+$, 11), 290 (18), 289 ($(M-C_3H_7)^+$, 75), 274 ($C_{18}H_{10}OS^+$, 26), 273 (22), 261 (30), 259 ($C_{18}H_7OS^+$, 12), 258 (13), 247 (10), 245 (13), 239 (11), 189 (10), 165 (22), 152 (17). IR (solid): $\tilde{\nu}$ 2978 (w) [cm^{-1}], 2970 (w), 2924 (w), 2866 (w), 2178 (m), 1585 (m), 1522 (w), 1458 (w), 1396 (s), 1369 (s), 1298 (s), 1246 (m), 1246 (m), 1196 (s), 1159 (m), 1134 (w), 1088 (m), 1074 (w), 1001 (m), 966 (m), 952 (m), 934 (w), 858 (m), 839 (m), 822 (w), 758 (m), 736 (m), 714 (m), 700 (s), 683 (m). Anal. calcd. for $C_{22}H_{20}OS$ (332.5): C 79.48, H 6.06. Found: C 79.39, H 6.22.

2.2.10 1-[3,8-Dimethyl-5-(propan-2-yl)azulen-1-yl]-3-(pyridin-3-yl)prop-2-yn-1-one (3j)

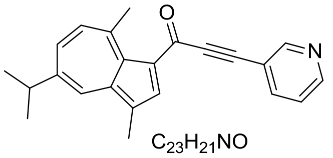

$C_{23}H_{21}NO$
327.42

202 mg (0.62 mmol, 31%) as a brown oil. A brown solid was obtained after further purification by suspension in *n*-pentane, followed by sonication in an ultrasound bath, filtration and drying in vacuo overnight. Mp. 64 °C. ^{1}H NMR ($CDCl_3$, 300 MHz): δ 1.39 (d, *J* = 6.9 Hz, 6 H), 2.61 (d, *J* = 0.6 Hz, 3 H), 3.05 (s, 3 H), 3.16 (sept, *J* = 6.9 Hz, 1 H), 7.35 (ddd, *J* = 7.9 Hz, *J* = 4.9 Hz, *J* = 0.8 Hz, 1 H), 7.49 (d, *J* = 11.0 Hz, 1 H), 7.65 (dd, *J* = 11.0 Hz, *J* = 2.1 Hz, 1 H), 7.96 (dt, *J* = 7.9 Hz, *J* = 2.0 Hz, 1 H), 8.29 (d, *J* = 2.1 Hz, 1 H), 8.35 (s, 1 H), 8.64 (dd, *J* = 4.8 Hz, *J* = 1.4 Hz, 1 H), 8.90 (d, *J* = 1.2 Hz, 1 H). ^{13}C NMR ($CDCl_3$, 75 MHz): δ 12.9 (CH_3), 24.5 (CH_3), 28.8 (CH_3), 38.1 (CH), 84.5 (C_{quat}), 93.2 (C_{quat}), 118.7 (C_{quat}), 123.2 (CH), 125.0 (C_{quat}), 126.1 (C_{quat}), 133.9 (CH), 134.7 (CH), 136.8 (CH), 137.9 (C_{quat}), 139.5 (CH), 143.8 (C_{quat}), 143.9 (CH), 147.0 (C_{quat}), 149.9 (CH), 150.3 (C_{quat}), 153.0 (CH), 171.4 (C_{quat}). EI + MS (*m/z* (%)): 328 (24), 327 (M^+, 100), 326 ($(M-H)^+$, 55), 312 ($(M-CH_3)^+$, 53), 310 (24), 299 ($(M-CO)^+$, 4), 297 ($C_{21}H_{15}NO^+$, 8), 296 (14), 284 ($(M-C_3H_7)^+$, 52), 277 (15), 269 ($C_{11}H_{19}NO^+$, 21), 268 (26), 267 (13), 256 ($C_{19}H_{14}N^+$, 15), 254 (18), 242 (16), 241 (14), 226 (10), 225 ($C_{17}H_{16}O^+$, 11), 189 (11), 183 (13), 165 (32), 153 (13), 152 (22), 149 (19), 141 (13), 130 ($(M-C_{15}H_{17})^+$, 6), 129 (14), 128 (25), 127 (14), 121 (10), 119 (11), 115 (12), 113 (12), 102 ($C_7H_4N^+$, 6), 101 (11), 43 (10). IR (solid): $\tilde{\nu}$ 2961 (w) [cm^{-1}], 2928 (w), 2868 (w), 2722 (w), 2398 (w), 2201 (w), 1601 (s), 1578 (m), 1532 (m), 1520 (m), 1472 (m), 1395 (s), 1358 (s), 1325 (m), 1300 (m), 1262 (m), 1190 (m), 1167 (s), 1088 (m), 1055 (w), 1018 (m), 999 (m), 972 (m), 955 (m), 918 (m), 897 (m), 878 (m), 828 (m), 812 (m), 797 (w), 768 (s), 756 (m), 733 (m), 696 (m), 642 (s), 623 (m). Anal. calcd. for $C_{23}H_{21}NO$ (327.4): C 84.37, H 6.46, N 4.28. Found: C 84.18, H 6.67, N 4.00.

2.2.11 1-[3,8-Dimethyl-5-(propan-2-yl)azulen-1-yl]hept-2-yn-1-one (3k)

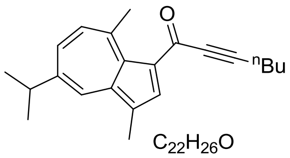

$C_{22}H_{26}O$
306.44

344 mg (1.12 mmol, 56%) as a brown oil. ^{1}H NMR ($CDCl_3$, 300 MHz): δ 0.98 (t, *J* = 7.3 Hz, 3 H), 1.37 (d, *J* = 6.9 Hz, 6 H), 1.45–1.59 (m, 2 H), 1.62–1.73 (m, 2 H), 2.50 (t, *J* = 7.0 Hz, 2 H), 2.59 (d, *J* = 0.6 Hz, 3 H), 2.99 (s, 3 H), 3.13 (sept, *J* = 6.9 Hz, 1 H), 7.38 (d, *J* = 11.0 Hz, 1 H), 7.58 (dd, *J* = 11.0 Hz, *J* = 2.0 Hz, 1 H), 8.25 (d, *J* = 2.1 Hz, 1 H), 8.26 (s, 1 H). ^{13}C NMR ($CDCl_3$, 75 MHz): δ 12.9 (CH_3), 13.5 (CH_3), 18.9 (CH_2), 22.1 (CH_2), 24.4 (CH_3), 28.7 (CH_3), 30.1 (CH_2), 38.0 (CH), 83.0 (C_{quat}), 91.8 (C_{quat}), 124.4 (C_{quat}), 126.6 (C_{quat}), 133.1 (CH), 134.4 (CH), 136.4 (CH), 137.1 (C_{quat}), 143.0 (C_{quat}), 143.9 (C_{quat}), 146.0 (CH), 150.0 (C_{quat}), 173.1 (C_{quat}). GC-MS (*m/z* (%)): 307 (21), 306 (M^+, 85), 291 ($(M-CH_3)^+$, 30), 289 (14), 278 ($(M-CO)^+$, 2), 277 ($(M-C_2H_5)^+$, 9), 264 (16), 263 ($(M-C_3H_7)^+$, 36), 249 ($(M-C_4H_9)^+$, 32), 248 ($C_{16}H_{18}O^+$, 10), 247 (12), 238 (19), 237 ($C_{17}H_{17}O^+$, 100), 236 (12), 235 (38), 233 (13), 225 ($C_{16}H_{17}O^+$, 4), 222 (30), 221 (57), 220 (21), 219 (23), 207 (22), 206 (15), 205 (26), 204 (14), 203 (23), 202 (23), 193 (14), 192 (17), 191 (25), 190 (17), 189 (28), 179 (20), 178 (25), 167 (10), 166 (13), 165 (41), 153 (12), 152 (21). IR (oil): $\tilde{\nu}$ 2957 (m) [cm^{-1}], 2928 (w), 2868 (w), 2207 (w), 1605 (m), 1522 (m), 1462 (w), 1398 (s), 1371 (s), 1325 (m), 1300 (w), 1248 (w), 1219 (m), 1192 (s), 1136 (w), 1092 (w), 1055 (w), 1026 (w), 997 (m), 958 (w), 931 (w), 866 (w), 847 (w), 818 (w), 781 (s), 760 (m), 736 (m), 689 (w), 640 (m). Anal. calcd. for $C_{22}H_{26}O$ (306.4): C 86.23, H 8.55. Found: C 86.13, H 8.29.

2.2.12 3-Cyclopropyl-1-[3,8-dimethyl-5-(propan-2-yl)azulen-1-yl]prop-2-yn-1-one (3l)

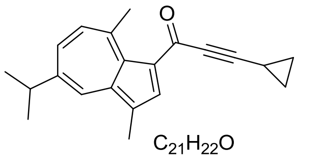

$C_{21}H_{22}O$
290.40

245 mg (0.85 mmol, 42%) as a brown solid. Mp. 110. °C. ^{1}H NMR ($CDCl_3$, 300 MHz): δ 0.97–1.00 (m, 2 H), 1.01 (s, 2 H), 1.37 (d, *J* = 6.9 Hz, 6 H), 1.48–1.57 (m, 1 H), 2.58 (d, *J* = 0.6 Hz, 3 H), 2.97 (s, 3 H), 3.13 (sept, *J* = 6.9 Hz, 1 H), 7.37 (d, *J* = 11.0 Hz, 1 H), 7.58 (dd, *J* = 11.0 Hz, *J* = 2.1 Hz, 1 H), 8.22 (s, 1 H), 8.24 (d, *J* = 2.1 Hz, 1 H). ^{13}C NMR ($CDCl_3$, 75 MHz): δ 0.0 (CH), 9.4 (CH_2), 13.0 (CH_3), 24.5 (CH_3), 28.7 (CH_3), 38.0 (CH), 78.7 (C_{quat}), 95.8 (C_{quat}), 124.4 (C_{quat}), 126.7 (C_{quat}), 133.0 (CH), 134.4 (CH), 136.4 (CH), 137.0 (C_{quat}), 142.9 (C_{quat}), 143.7 (CH), 145.9 (C_{quat}), 149.6 (C_{quat}), 173.1 (C_{quat}). EI + MS (*m/z* (%)): 291 (19), 290 (M^+, 82), 175 ($(M-CH_3)^+$, 18), 273 (12), 262 ($(M-CO)^+$ 12), 261 (23), 248 (15), 247 ($(M-C_3H_7)^+$, 80), 245 (19), 238 (18), 237 (100), 232 (27), 231 (19), 225 ($C_{17}H_{16}O^+$, 2), 222 (15), 220 (15), 219 (85), 217 ($C_{16}H_9O^+$, 12), 215 (18), 205 (16), 204 (14), 203 (29), 202 (37), 191 (18), 190 (18), 188 (38), 179 (12), 178 (18), 176 (11), 166 (14), 165 (47), 153 (12), 152 (28), 128 (10), 115 (14), 110 (11), 101 (17), 94 (14), 89 (13), 65 ($C_5H_5O^+$, 5). IR (solid): $\tilde{\nu}$ 3015 (w) [cm^{-1}], 2965 (m), 2930 (w), 2903 (w), 2855 (w), 2778 (w), 2579 (w), 2220 (w), 2191(s), 2593 (s), 1522 (m), 1460 (w), 1400 (s), 1366 (s), 1327 (m), 1300 (m), 1225 (s), 1209 (m), 1171 (s), 1132 (m), 1092 (m), 1059 (m), 1026 (m), 1013 (m), 989 (m), 934 (m), 918 (m), 853 (s), 829 (m), 812 (m), 791 (s), 758 (s), 739 (s), 739 (s), 712 (m), 685 (m), 642 (m). Anal. calcd. for $C_{21}H_{22}O$ (290.4): C 86.85, H 7.64. Found: C 86.95, H 7.62.

2.2.13 1-[3,8-Dimethyl-5-(propan-2-yl)azulen-1-yl]-4,4-diethoxybut-2-yn-1-one (3m)

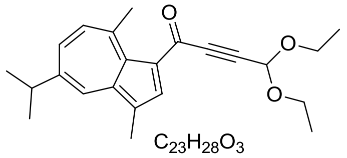

$C_{23}H_{28}O_3$
352.47

212 mg (0.60 mmol, 30%) as a brown oil. ^{1}H NMR ($CDCl_3$, 300 MHz): δ 1.30 (t, *J* = 7.1 Hz, 6 H), 1.38 (d, *J* = 6.9 Hz, 6 H), 2.57 (d, *J* = 0.6 Hz, 3 H), 3.00 (s, 3 H), 3.14 (sept, *J* = 6.9 Hz, 1 H), 3.72 (dq, *J* = 9.5 Hz, *J* = 7.1 Hz, 2 H), 3.87 (dq, *J* = 9.5 Hz, *J* = 7.1 Hz, 2 H), 5.54 (s, 1 H), 7.43 (d, *J* = 11.1 Hz, 1 H), 7.62 (dd, *J* = 11.0 Hz, *J* = 2.1 Hz, 1 H), 8.26 (d, *J* = 2.1 Hz, 1 H), 8.28 (s, 1 H). ^{13}C NMR ($CDCl_3$, 75 MHz): δ 12.9 (CH_3), 15.1 (CH_3), 24.5 (CH_3), 28.8 (CH_3), 38.1 (CH), 61.4 (CH_2), 82.9 (CH), 85.3 (C_{quat}), 91.5 (C_{quat}), 124.9 (C_{quat}), 125.9 (C_{quat}), 133.8 (CH), 134.6 (CH), 136.7 (CH), 137.8 (C_{quat}), 143.7 (C_{quat}), 144.3 (CH), 146.9 (C_{quat}), 150.2 (C_{quat}), 171.0 (C_{quat}). GC-MS (*m/z* (%)): 353 (23), 352 (M^+, 100), 337 ($(M-CH_3)^+$, 1), 309 ($(M-C_3H_7)^+$, 1), 307 ($(M-C_2H_5O)^+$, 3), 280 (17), 279 (83), 278 (22), 277 (52), 263 (12), 262 (11), 261 (11), 251 (16), 250 (30), 249 ($(M-C_5H_{11}O_2)^+$, 20), 247 (11), 236 (19), 235 (64), 234 (14), 233 (16), 225 ($C_{16}H_{17}O^+$, 31), 222 (10), 221 (34), 220 (10), 219 (21), 209 (10), 207 (30), 206 (13), 205 (15), 202 (12), 192 (24), 191 (33), 190 (15), 189 (23), 181 (13), 179 (23), 178 (26), 167 (19), 166 (19), 165 (47), 153 (13), 152 (27). IR (oil): $\tilde{\nu}$ 2963 (w) [cm^{-1}], 2926 (w), 2887 (w), 2872 (w), 2176 (w), 1728 (w), 1608 (s), 1533 (m), 1460 (w), 1442 (w), 1402 (s), 1364 (s), 1325 (m), 1302 (w), 1219 (w), 1184 (s), 1134 (m), 1094 (s), 1051 (s), 1014 (s), 947 (w), 920 (w), 897 (w), 876 (w), 860 (w), 847 (w), 814 (m), 760 (w), 710 (w), 689 (w), 664 (w), 640 (w), 619 (w). Anal. calcd. for $C_{23}H_{28}O_3$ (352.5): C 78.38, H 8.01. Found: C 78.46, H 8.12.

2.2.14 1-[3,8-Dimethyl-5-(propan-2-yl)azulen-1-yl]-3-[tris(propan-2-yl)silyl]prop-2-yn-1-one (3n)

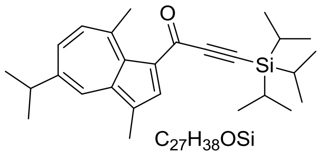

$C_{27}H_{38}OSi$
406.68

213 mg (0.52 mmol, 26%) as a brown oil. A brown solid was obtained after further purification by suspension in *n*-pentane, followed by sonication in an ultrasound bath, filtration and drying in vacuo overnight. Mp. 87 °C. ^{1}H NMR ($CDCl_3$, 300 MHz): δ 1.17–1.21 (m, 21 H), 1.39 (d, *J* = 6.9 Hz, 6 H), 2.58 (s, 3 H), 3.01 (s, 3 H), 3.14 (sept, *J* = 6.9 Hz, 1 H), 7.42 (d, *J* = 11.0 Hz, 1 H), 7.61 (dd, *J* = 11.0 Hz, *J* = 2.1 Hz, 1 H), 8.25 (d, *J* = 2.1 Hz, 1 H), 8.34 (s, 1 H). ^{13}C NMR ($CDCl_3$, 75 MHz): δ 11.2 (CH), 13.0 (CH_3), 18.7 (CH_3), 24.5 (CH_3), 28.9 (CH_3), 38.1 (CH), 91.9 (C_{quat}), 107.0 (C_{quat}), 124.7 (C_{quat}), 126.5 (C_{quat}), 133.5 (CH), 134.5 (CH), 136.6 (CH), 137.5 (C_{quat}), 143.4 (C_{quat}), 144.4 (CH), 146.4 (C_{quat}), 150.1 (C_{quat}), 172.0 (C_{quat}). EI + MS (*m/z* (%)): 407 (34), 406 (M^+, 100), 391 ($(M-CH_3)^+$, 6), 364 (22), 363 ($(M-C_3H_7)^+$, 68), 333 (11), 349 (15), 322 (17), 321 (62), 279 (29), 277 (25), 275 (20), 261 (17), 249 (16), 235 (18), 234 (21), 233 (17), 225 ($C_{16}H_{17}O^+$, 77), 210 (12), 209 ($C_{12}H_{22}OSi^+$, 10), 197 ($C_{15}H_{17}^+$, 3), 182 (21), 181 ($C_{11}H_{21}Si^+$, 14), 167 (26), 166 (12), 165 (29), 155 (10), 154 (71), 152 (17), 146 (23), 132 (16), 131 (15), 125 (30), 118 (14). IR (solid): $\tilde{\nu}$ 2957 (m) [cm^{-1}], 2940 (m), 2926 (m), 2862 (m), 2139 (w), 1732 (w), 1601 (s), 1533 (m), 1520 (m), 1458 (m), 1396 (s), 1369 (s), 1302 (w), 1254 (w), 1242 (w), 1219 (w), 1171 (s), 1130 (w), 1111 (m), 1090 (m), 1072 (w), 1059 (w), 995 (m), 974 (m), 953 (m), 928 (w), 918 (w), 881 (m), 866 (s), 822 (w), 762 (m), 737 (w), 714 (w), 679 (m), 662 (m), 654 (m), 642 (w). Anal. calcd. for $C_{27}H_{38}OSi$ (406.7): C 79.74, H 9.42. Found: C 79.91, H 9.32.

3 Preparation of pyrimidines 5

3.1 General procedure for the synthesis of pyrimidines 5

1.00 mmol of azulene **1** (130 mg azulene (**1a**), 202 mg guaiazulene (**1b**)) in dry 1,4-dioxane (5 mL/mmol) was placed under an argon atmosphere in a screw-cap Schlenk tube and degassed with argon. Then, oxalyl chloride (0.09 mL, 1.00 mmol, 1.00 equiv) was added dropwise to the reaction mixture at rt (water bath). The mixture was stirred for 4 h. Thereafter, $PdCl_2(PPh_3)_2$ (14 mg, 0.02 mmol, 2 mol %), CuI (4 mg, 0.02 mmol, 2 mol %), one equiv of terminal alkyne **2** and dry triethylamine (0.28 mL, 2.00 mmol, 2.00 equiv) were successively added to the reaction mixture and stirring at rt was continued for 1 h. Then, 1 mL of 2-methoxyethanol, potassium carbonate (349 mg, 2.50 mmol, 2.50 equiv) and one equiv of amidine hydrochloride **4** were added, and the reaction mixture was stirred at 100 °C (preheated oil bath). After 24 h the reaction progress was monitored by TLC. Due to incomplete conversion another equiv of amidine hydrochloride was added with 1 mL of 2-methoxyethanol and stirring was continued for 24 h. After removal of the solvents in vacuo the residue was absorbed onto Celite® and purified by chromatography on silica gel with petroleum ether (boiling range 40–60 °C)/ethyl acetate and 1% triethylamine to give the pyrimidines **5**.

Table 2: Experimental details for the one-pot four-component synthesis of pyrimidines **5**.

Entry	Alkyne **2**	Amidine hydrochloride **4**	Pyrimidine **5** (isolated yield)	Chromatographic purification (eluent) R_f (eluent)
1	Phenyl-acetylene (**2a**) 0.11 mL (1.00 mmol) Merck	Benzamidine hydrochloride (**4a**) 254 mg (2.00 mmol) Acros	243 mg (0.68 mmol, 68%) 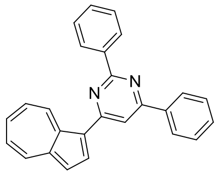 **5a**	(PE/EtOAc) = 15:1, 1% NEt_3 R_f (PE/EtOAc 15:1) = 0.23
2	**2a** 0.11 mL (1.00 mmol)	2-Thienylamidine hydrochloride (**4b**) 651 mg (2.00 mmol)	136 mg (0.37 mmol, 37%) 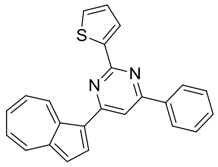 **5b**	(PE/EtOAc) = 15:1, 1% NEt_3 R_f (PE/EtOAc 15:1) = 0.31
3	**2a** 0.11 mL (1.00 mmol)	Acetamidine hydrochloride (**4c**) 195 mg (2.00 mmol) Alfa Aesar	133 mg (0.47 mmol, 47%) 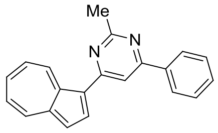 **5c**	(PE/EtOAc) = 15:1, 1% NEt_3 R_f (PE/EtOAc 15:1) = 0.21
4	Hexyne **2b** 0.12 mL (1.00 mmol) Alfa Aesar	(**4a**) 254 mg (2.00 mmol)	162 mg (0.48 mmol, 48%) 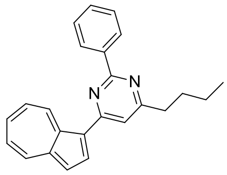 **5d**	(PE/EtOAc) = 15:1, 1% NEt_3 R_f (PE/EtOAc 15:1) = 0.20

Table 2 (continued). Experimental details for the one-pot four-component synthesis of pyrimidines **5**.

Entry	Alkyne **2**	Amidine hydrochloride **4**	Pyrimidine **5** (isolated yield)	Chromatographic purification (eluent) R_f (eluent)
5	**2a**<br>0.11 mL<br>(1.00 mmol)	**4a**<br>254 mg<br>(2.00 mmol)	141 mg<br>(0.33 mmol, 33%)<br>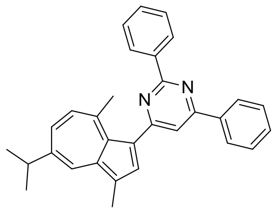<br>**5e**	(PE/EtOAc) = 50:1, 1% NEt_3<br>R_f (PE/EtOAc 50:1) = 0.20
6	**2b**<br>0.12 mL<br>(1.00 mmol)	**4a**<br>254 mg<br>(2.00 mmol)	198 mg<br>(0.48 mmol, 48%)<br>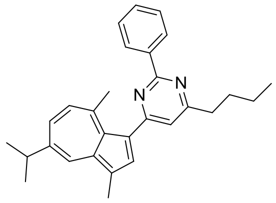<br>**5f**	(PE/EtOAc) = 100:1, 1% NEt_3<br>R_f (PE/EtOAc 100:1) = 0.04

3.2 Spectroscopic data of pyrimidines 5

3.2.1 4-(Azulen-1-yl)-2,6-diphenylpyrimidine (5a)

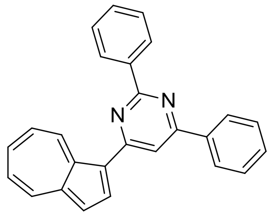

$C_{26}H_{18}N_2$
358.43

243 mg (0.68 mmol, 68%) as a green solid. Mp. 69 °C. ^{1}H NMR ($CDCl_3$, 300 MHz): δ 7.37 (t, *J* = 9.6 Hz, 1 H), 7.48 (d, *J* = 9.7 Hz, 1 H) 7.52–7.62 (m, 7 H), 7.79 (t, *J* = 9.8 Hz, 1 H), 8.04 (s, 1 H), 8.29–8.34 (m, 2 H), 8.45–8.50 (m, 2 H), 8.73–8.77 (m, 2 H), 10.20 (d, *J* = 10.0 Hz, 1 H). ^{13}C NMR ($CDCl_3$, 75 MHz): δ 112.3 (CH), 118.5 (CH), 125.3 (C_{quat}), 125.6 (CH), 126.7 (CH), 127.2 (CH), 128.4 (CH), 128.5 (CH), 128.8 (CH), 130.4 (CH), 130.5 (CH), 137.2 (CH), 137.9 (C_{quat}), 138.0 (CH), 138.7 (C_{quat}), 138.7 (CH), 139.2 (CH), 144.7 (C_{quat}), 163.8 (C_{quat}), 163.8 (C_{quat}), 164.1 (C_{quat}). EI + MS (*m/z* (%)): 359 (25), 358 (M^+, 100), 357 ($(M-H)^+$, 97), 281 ($(M-C_6H_5)^+$, 26), 255 ($(M-C_7H_5N)^+$, 15), 254 (67), 253 (15), 155 (11), 153 (16), 152 (26), 151 (23), 128 (10), 127 ($C_{10}H_7^+$, 29), 126 (19), 125 (16), 111 (17), 109 (11), 103 ($C_7H_5N^+$, 25), 97 (25), 95 (17), 85 (19), 83 (21), 81 (14), 77 ($C_6H_5^+$, 12), 71 (23), 69 (18), 57 (26), 43 (21). IR (solid): $\tilde{\nu}$ 3063 (w) [cm^{-1}], 2980 (w), 2972 (w), 2889 (w), 2417 (w), 2305 (w), 1587 (w), 1564 (m), 1537 (w), 1520 (m), 1493 (m), 1470 (w), 1422 (m), 1395 (m), 1371 (m), 1337 (w), 1302 (w), 1275 (w), 1225 (w), 1207 (w), 1171 (w), 1152 (w), 1069 (w), 1028 (w), 986 (w), 959 (w), 943 (w), 926 (w), 881 (w), 860 (w), 774 (m), 750 (s), 689 (s), 673 (w), 658 (m), 635 (m). Calcd. for $C_{26}H_{18}N_2$ (358.4): C 87.12, H 5.06, N 7.82. Found: C 86.94, H 5.31, N 7.84.

3.2.2 4-(Azulen-1-yl)-6-phenyl-2-(thiophen-2-yl)pyrimidine (5b)

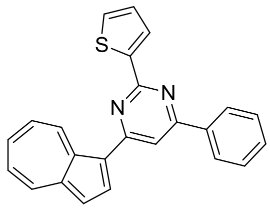

$C_{24}H_{16}N_2S$
364.46

136 mg (0.37 mmol, 37%) as a green-violet solid. Mp. 143 °C. ^{1}H NMR ($CDCl_3$, 300 MHz): δ 7.23 (dd, *J* = 5.0 Hz, *J* = 3.7 Hz, 1 H), 7.36 (t, *J* = 9.6 Hz, 1 H), 7.44 (d, *J* = 4.1 Hz, 1 H), 7.50–7.61 (m, 5 H), 7.78 (t, *J* = 9.8 Hz, 1 H), 7.94 (s, 1 H), 8.22 (dd, *J* = 3.6 Hz, *J* = 1.2 Hz, 1 H), 8.24–8.28 (m, 2 H), 8.42–8.45 (m, 2 H), 10.25 (d, *J* = 10.0 Hz, 1 H). ^{13}C NMR ($CDCl_3$, 75 MHz): δ 111.4 (CH), 118.5 (CH), 124.6 (C_{quat}), 125.7 (CH), 126.8 (CH), 127.1 (CH), 128.1 (CH), 128.4 (CH), 128.8 (CH), 129.2 (CH), 130.5 (CH), 137.0 (CH), 137.6 (C_{quat}), 138.0 (CH), 138.2 (C_{quat}), 139.1 (CH), 139.2 (CH), 144.8 (C_{quat}), 144.9 (C_{quat}), 161.0 (C_{quat}), 163.6 (C_{quat}), 163.7 (C_{quat}). EI + MS (*m/z* (%)): 365 (27), 364 (M^+, 100), 363 ($(M-H)^+$, 68), 287 ($(M-C_6H_5)^+$, 18), 255 ($(M-C_5H_3NS)^+$, 14), 254 (60), 253 (13), 153 (11), 152 (16), 151 (13), 127 ($C_{10}H_7^+$, 12), 126 (10), 77 ($C_6H_5^+$, 4), 57 (12). IR (solid): $\tilde{\nu}$ [cm^{-1}] 3048 (w), 2957 (w), 2922 (w), 2855 (w), 2359 (w), 2330 (w), 1688 (w), 1557 (m), 1537 (m), 1516 (m), 1495 (m), 1466 (w), 1443 (m), 1420 (m), 1396 (s), 1375 (m), 1329 (m), 1277 (w), 1223 (m), 1207 (w), 1150 (w), 1057 (w), 1024 (w), 980 (w), 858 (m), 843 (w), 806 (w), 770 (s), 746 (m), 706 (s). Anal. calcd. for $C_{24}H_{16}N_2S$ (364.5): C 79.09, H 4.42, N 7.69. Found: C 78.87, H 4.66, N 7.41.

3.2.3 4-(Azulen-1-yl)-2-methyl-6-phenylpyrimidine (5c)

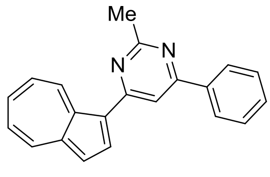

$C_{21}H_{16}N_2$
296.37

133 mg (0.47 mmol, 47%) as a blue-violet solid. Mp. 106 °C. ^{1}H NMR ($CDCl_3$, 300 MHz): δ 2.91 (s, 3 H), 7.33 (t, J = 9.7 Hz, 1 H), 7.43–7.59 (m, 5 H), 7.75 (t, J = 9.8 Hz, 1 H), 7.90 (s, 1 H), 8.13–8.17 (m, 2 H), 8.40–8.44 (m, 2 H), 9.97 (d, J = 10.0 Hz, 1 H). ^{13}C NMR ($CDCl_3$, 75 MHz): δ 26.6 (CH_3), 111.9 (CH), 118.4 (CH), 125.1 (C_{quat}), 125.4 (CH), 126.5 (CH), 127.1 (CH), 128.8 (CH), 130.2 (CH), 137.2 (CH), 137.8 (C_{quat}), 137.9 (CH), 138.0 (C_{quat}), 138.5 (CH), 139.1 (CH), 144.5 (C_{quat}), 163.6 (C_{quat}), 164.0 (C_{quat}), 167.9 (C_{quat}). EI + MS (*m/z* (%)): 297 (21), 296 (M^+, 100), 295 ($(M-H)^+$, 73), 281 ($(M-CH_3)^+$, 2), 255 ($(M-C_2H_3N)^+$, 15), 254 (67), 253 (12), 219 ($(M-C_6H_5)^+$, 13), 152 (11), 127 ($C_{10}H_7^+$, 16), 77 ($C_6H_5^+$, 2). IR (solid): $\tilde{\nu}$ 3055 (w) [cm^{-1}], 2920 (w), 2851 (w), 2365 (w), 1560 (s), 1535 (m), 1522 (s), 1497 (m), 1464 (w), 1441 (w), 1420 (m), 1396 (m), 1337 (w), 1277 (w), 1221 (w), 1198 (w), 1070 (w), 1028 (w), 985 (w), 945 (w), 926 (w), 856 (w), 787 (s), 762 (s), 741 (m), 694 (s), 669 (m), 640 (m), 615 (w). Anal. calcd. for $C_{21}H_{16}N_2$ (296.4): C 85.11, H 5.44, N 9.45. Found: C 84.92, H 5.64, N 9.67.

3.2.4 4-(Azulen-1-yl)-6-butyl-2-phenylpyrimidine (5d)

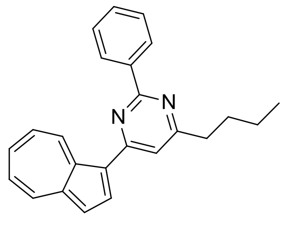

$C_{24}H_{22}N_2$
338.44

162 mg (0.48 mmol, 48%) as a blue-violet solid. Mp. 84 °C. ^{1}H NMR ($CDCl_3$, 300 MHz): δ 1.02 (t, *J* = 7.3 Hz, 3 H), 1.51 (sext, *J* = 7.5 Hz, 2 H), 1.84–1.94 (m, 2 H), 2.89 (t, *J* = 7.7 Hz, 2 H), 7.34 (t, *J* = 9.6 Hz, 1 H), 7.44 (d, *J* = 4.1 Hz, 1 H), 7.49 (d, *J* = 11.0 Hz, 1 H), 7.53–7.59 (m, 4 H), 7.76 (t, *J* = 9.8 Hz, 1 H), 8.40 (d, *J* = 4.2 Hz, 1 H), 8.43 (d, *J* = 9.5 Hz, 1 H), 8.63 (dd, *J* = 7.6 Hz, *J* = 1.4 Hz, 2 H), 10.16 (d, *J* = 10.0 Hz, 1 H). ^{13}C NMR ($CDCl_3$, 75 MHz): δ 14.0 (CH_3), 22.5 (CH_2), 31.1 (CH_2), 38.0 (CH_2), 115.2 (CH), 118.4 (CH), 125.2 (C_{quat}) 125.3 (CH), 126.4 (CH), 128.3 (CH), 128.4 (CH), 130.1 (CH), 137.1 (CH), 137.9 (CH), 138.7 (CH), 138.9 (C_{quat}), 139.0 (CH), 144.5 (C_{quat}), 162.9 (C_{quat}), 163.9 (C_{quat}), 170.6 (C_{quat}). EI + MS (*m/z* (%)): 338 (M^+, 13), 323 ($(M-CH_3)^+$, 3), 309 ($(M-C_2H_5)^+$, 10), 297 (23), 296 (100), 295 ($(M-C_3H_7)^+$, 27), 281 ($(M-C_4H_9)^+$, 3), 233 (10), 152 (12), 103 (11). IR (solid): $\tilde{\nu}$ 3061 (w) [cm^{-1}], 2955 (w), 2926 (w), 2855 (w), 1584 (m), 1566 (s), 1541 (m), 1518 (m), 1495 (s), 1468 (m), 1425 (m), 1396 (s), 1375 (s), 1335 (m), 1310 (s), 1290 (m), 1273 (m), 1209 (w), 1140 (w), 1024 (w), 988 (w), 947 (w), 930 (w), 851 (m), 793 (m), 752 (s), 694 (s), 673 (m), 656 (m). Anal. calcd. for $C_{24}H_{22}N_2$ (338.4): C 85.17, H 6.55, N 8.28. Found: C 85.06, H 6.64, N 8.39.

3.2.5 4-[3,8-Dimethyl-5-(propan-2-yl)azulen-1-yl]-2,6-diphenylpyrimidine (5e)

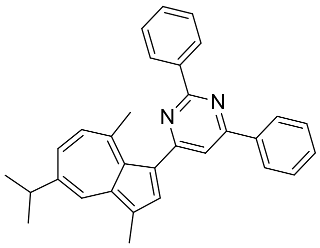

$C_{31}H_{28}N_2$
428.57

141 mg (0.33 mmol, 33%) as a dark green oil. A dark green solid was obtained after recrystallization from ethanol. Mp. 118 °C. ^{1}H NMR ($CDCl_3$, 300 MHz): δ 1.42 (d, J = 6.9 Hz, 6 H), 2.72 (s, 3 H), 2.74 (s, 3 H), 3.16 (sept, J = 6.9 Hz, 1 H), 7.18 (d, J = 10.8 Hz, 1 H), 7.51–7.59 (m, 7 H), 7.84 (s, 1 H), 7.92 (s, 1 H), 8.31–8.34 (m, 3 H), 8.70–8.74 (m, 2 H). ^{13}C NMR ($CDCl_3$, 75 MHz): δ 12.9 (CH_3), 24.6 (CH_3), 28.5 (CH_3), 38.0 (CH), 115.4 (CH), 124.9 (C_{quat}) 126.1 (C_{quat}), 127.2 (CH), 128.3 (CH), 128.4 (CH), 128.8 (CH), 130.4 (CH), 130.5 (CH), 133.6 (C_{quat}), 134.3 (CH), 135.5 (CH), 137.7 (C_{quat}), 138.4 (C_{quat}), 139.7 (C_{quat}), 140.2 (CH), 142.1 (C_{quat}), 146.8 (C_{quat}), 162.4 (C_{quat}), 163.2 (C_{quat}), 166.9 (C_{quat}). EI + MS (*m/z* (%)): 429 (28), 428 (M^+, 100), 427 ($(M-H)^+$, 83), 413 ($(M-CH_3)^+$, 14), 398 ($C_{29}H_{22}N_2^+$, 2), 385 ($(M-C_3H_7)^+$, 7), 351 ($(M-C_6H_5)^+$, 15), 325 ($(M-C_7H_5N)^+$, 2), 310 (15), 309 (22), 308 (14), 233 (18), 149 (11), 134 (10), 133 (53), 132 (44), 105 (15), 104 (20), 103 (51), 77 ($C_6H_5^+$, 13). IR (solid): $\tilde{\nu}$ 3026 (w) [cm^{-1}], 2959 (w), 2924 (w), 2901 (w), 2864 (w), 1709 (w), 1587 (m), 1560 (s), 1518 (s), 1497 (m), 1458 (m), 1445 (m), 1352 (m), 1314 (w), 1285 (w), 1244 (w), 1196 (m), 1173 (w), 1155 (w), 1099 (w), 1074 (w), 1053 (m), 1028 (m), 968 (w), 912 (w), 858 (m), 837 (m), 822 (m), 808 (w), 781 (m), 760 (s), 700 (s), 687 (s), 665 (m), 654 (s), 631 (s). Anal. calcd. for $C_{31}H_{28}N_2$ (428.6): C 86.88, H 6.59, N 6.54. Found: C 86.72, H 6.53, N 6.44.

3.2.6 4-Butyl-6-[3,8-dimethyl-5-(propan-2-yl)azulen-1-yl]-2-phenylpyrimidine (5f)

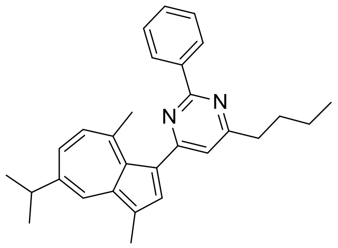

$C_{29}H_{32}N_2$
408.58

198 mg (0.48 mmol, 48%) as a green-blue oil. A dark blue solid was obtained after recrystallization from ethanol. Mp. 88 °C. ^{1}H NMR ($CDCl_3$, 300 MHz): δ 1.02 (d, *J* = 7.4 Hz, 3 H), 1.41 (d, *J* = 6.9 Hz, 6 H), 1.50 (sext, *J* = 7.4 Hz, 2 H), 1.83–1.93 (m, 2 H), 2.67 (s, 3 H), 2.69 (s, 3 H), 2.88 (t, *J* = 7.8 Hz, 2 H), 3.14 (sept, *J* = 6.9 Hz, 1 H), 7.15 (d, *J* = 10.8 Hz, 1 H), 7.24 (s, 1 H), 7.46–7.53 (m, 4 H), 7.83 (s, 1 H), 8.28 (d, *J* = 1.5 Hz, 1 H), 8.57–8.59 (m, 2 H). ^{13}C NMR ($CDCl_3$, 75 MHz): δ 12.9 (CH_3), 14.0 (CH_3), 22.5 (CH_2), 24.6 (CH_3), 28.4 (CH_3), 31.0 (CH_2), 37.8 (CH_2), 37.9 (CH), 118.7 (CH), 124.8 (C_{quat}) 126.1 (C_{quat}), 128.3 (CH), 128.6 (CH), 130.1 (CH), 133.4 (C_{quat}), 134.2 (CH), 135.4 (CH), 138.5 (C_{quat}), 139.6 (C_{quat}), 140.2 (CH), 141.8 (C_{quat}), 146.7 (C_{quat}), 162.9 (C_{quat}), 166.0 (C_{quat}) 169.4 (C_{quat}). EI + MS (*m/z* (%)): 409 (19), 408 (M^+, 72), 407 ($(M\text{-}H)^+$, 100), 393 ($(M\text{-}CH_3)^+$, 8), 366 (11), 365 ($(M\text{-}C_3H_7)^+$, 18), 352 (11), 351 ($C_{25}H_{23}N_2^+$, 4), 335 ($C_{24}H_{21}N_2^+$, 6), 103 (26), 77 ($C_6H_5^+$, 13). IR (solid): $\tilde{\nu}$ 3065 (w) [cm^{-1}], 2955 (m), 2924 (w), 2860 (w), 1709 (w), 1587 (m), 1564 (s), 1541 (m), 1460 (m), 1445 (s), 1427 (m), 1373 (s), 1352 (m), 1288 (w), 1258 (w), 1215 (w), 1163 (m), 1105 (w), 1086 (w), 1055 (w), 1024 (m), 988 (w), 945 (w), 854 (s), 822 (m), 770 (s), 743 (s), 704 (s), 675 (m), 650 (s), 637 (s). Anal. calcd. for $C_{29}H_{32}N_2$ (408.6): C 85.25, H 7.89, N 6.86. Found: C 85.34, H 8.18, N 6.92.

4 Preparation of pyrazoles 7

4.1 General procedure for the synthesis of pyrazoles 7

Azulene (**1a**) (130 mg, 1.00 mmol) in dry 1,4-dioxane (5 mL/mmol) was placed under argon atmosphere in a screw-cap Schlenk tube and degassed with argon. Then, oxalyl chloride (0.09 mL, 1.00 mmol, 1.00 equiv) was added dropwise to the reaction mixture at rt (water bath) and the mixture was stirred for 4 h. Thereafter, $PdCl_2(PPh_3)_2$ (14 mg, 0.02 mmol, 2 mol %), CuI (4 mg, 0.02 mmol, 2 mol %), one equiv of terminal alkyne **2** and dry triethylamine (0.28 mL, 2.00 mmol, 2.00 equiv) were successively added to the reaction mixture, and stirring at rt was continued for 1 h. Then, 1 mL of 2-methoxyethanol and one equiv of methylhydrazine **6** were added, and the reaction mixture was stirred at 100 °C (preheated oil bath). After 24 h the reaction progress was monitored by TLC. Due to incomplete conversion another equiv of methylhydrazine **6** (1.00 mmol, 0.05 mL) was added with 1 mL 2-methoxyethanol and the reaction was continued for 24 h. After removal of the solvent in vacuo the residue was absorbed onto Celite® and purified chromatographically on silica gel with petroleum ether (boiling range 40-60 °C)/ethyl acetate and 1% triethylamine to give the pyrazoles **7**.

Table 3: Experimental details for the one-pot four-component synthesis of pyrazoles **7**.

Entry	Alkyne **2**	Pyrazoles **7** (isolated yield)	Chromatographic purification (eluent) R_f (eluent)
1	Phenylacetylene **2a**<br>0.11 mL, (1.00 mmol)<br>*Merck*	134 mg<br>(0.47 mmol, 47%)<br>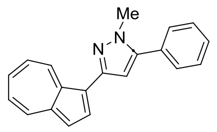<br><br>**7a**	(PE/EtOAc) = 10:1, 1% NEt_3<br>R_f (PE/EtOAc 10:1) = 0.19
2	4-Ethynylbenzonitrile **2d**<br>131 mg, (2.00 mmol)<br>*Sigma Aldrich*	0.127 mg<br>(0.41 mmol, 41%)<br>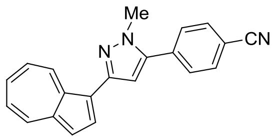<br><br>**7b**	(PE/EtOAc) = 5:1, 1% NEt_3<br>R_f (PE/EtOAc 5:1) = 0.09

4.2 Spectroscopic data of pyrazoles 7

4.2.1 3-(Azulen-1-yl)-1-methyl-5-phenyl-1*H*-pyrazole (7a)

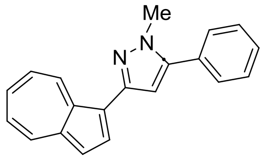

$C_{20}H_{16}N_2$
284.35

134 mg (0.47 mmol, 47%) as a blue solid. Mp. 119 °C. ^{1}H NMR ($CDCl_3$, 300 MHz): δ 4.02 (s, 3 H), 6.71 (s, 1 H), 7.15 (t, J = 9.6 Hz, 1 H), 7.26 (t, J = 9.8 Hz, 1 H), 7.41–7.64 (m, 7 H), 8.21 (d, J = 3.9 Hz, 1 H), 8.32 (d, J = 9.4 Hz, 1 H), 9.33 (d, J = 9.8 Hz, 1 H). ^{13}C NMR ($CDCl_3$, 75 MHz): δ 37.6 (CH_3), 105.2 (CH), 117.6 (CH), 122.2 (C_{quat}), 123.2 (CH), 123.8 (CH), 128.4 (CH), 128.7 (CH), 128.8 (CH), 130.9 (C_{quat}), 135.0 (C_{quat}), 136.3 (CH), 137.0 (CH), 137.1 (CH), 138.1 (CH), 142.3 (C_{quat}), 144.3 (C_{quat}), 148.3 (C_{quat}). EI + MS (*m/z* (%)): 285 (21), 284 (M^+, 100), 283 ($(M\text{-}H)^+$, 25), 269 ($(M\text{-}CH_3)^+$, 3), 242 (17), 241 ($(M\text{-}N_2CH_3)^+$, 8), 127 ($C_{10}H_7^+$, 3), 77 ($C_6H_5^+$, 4). IR (solid): $\tilde{\nu}$ 3057 (w) [cm^{-1}], 3028 (w), 2940 (w), 1954 (w), 1574 (w), 1555 (m), 1485 (w), 1454 (w), 1396 (s), 1339 (w), 1271 (m), 1070 (w), 1005 (m), 988 (w), 907 (w), 856 (m), 787 (s), 768 (s), 739 (s), 696 (s), 669 (m). Anal. calcd. for $C_{20}H_{16}N_2$ (284.4): C 84.48, H 5.67, N 9.85. Found: C 84.28, H 5.63, N 9.80.

4.2.2 3-{(Azulen-1-yl)-1-methyl-1*H*-pyrazol-4-yl}benzonitrile (7b)

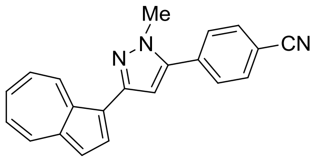

$C_{21}H_{15}N_3$
309.36

127 mg (0.41 mmol, 41%) as a blue solid. Mp. 131 °C. ^{1}H NMR ($CDCl_3$, 300 MHz): δ 4.02 (s, 3 H), 6.75 (s, 1 H), 7.17 (t, *J* = 9.7 Hz, 1 H), 7.26 (t, *J* = 9.7 Hz, 1 H), 7.41 (d, *J* = 4.0 Hz, 1 H), 7.59–7.64 (m, 3 H), 7.76–7.79 (m, 2 H), 8.17 (d, *J* = 4.0 Hz, 1 H), 8.33 (d, *J* = 9.4 Hz, 1 H), 9.38 (d, *J* = 9.8 Hz, 1 H). ^{13}C NMR ($CDCl_3$, 75 MHz): δ 37.9 (CH_3), 106.0 (CH), 112.0 (C_{quat}), 117.7 (CH), 118.4 (C_{quat}), 121.5 (C_{quat}), 123.5 (CH), 124.0 (CH), 129.1 (CH), 132.5 (CH), 135.0 (C_{quat}), 135.2 (C_{quat}), 136.2 (CH), 137.0 (CH), 137.1 (CH), 138.3 (CH), 142.3 (C_{quat}), 142.4 (C_{quat}), 148.7 (C_{quat}). EI + MS (*m/z* (%)): 310 (23), 209 (M^+, 100), 308 ($(M\text{-}H)^+$, 20), 294 ($(M\text{-}CH_3)^+$, 2), 267 (17), 266 ($(M\text{-}N_2CH_3)^+$, 3), 127 ($C_{10}H_7^+$, 3), 77 ($C_6H_5^+$, 4). IR (solid): $\tilde{\nu}$ 2988 (w) [cm^{-1}], 2936 (w), 1790 (w), 1609 (w), 1568 (w), 1551 (w), 1487 (w), 1470 (w), 1447 (w), 1427 (w), 1396 (m), 1339 (w), 1271 (w), 1179 (w), 1128 (w), 1105 (w), 1049 (w), 1005 (m), 970 (w), 943 (w), 897 (w), 844 (s), 835 (m), 773 (s), 741 (s), 681 (w). Anal. calcd. for $C_{21}H_{15}N_3$ (309.4): C 81.53, H 4.89, N 13.58. Found: C 81.31, H 4.94, N 13.46.

5 ^{1}H and ^{13}C NMR Spectra of Ynones 3

5.1 1-(Azulen-1-yl)-3-phenylprop-2-yn-1-one (3a)

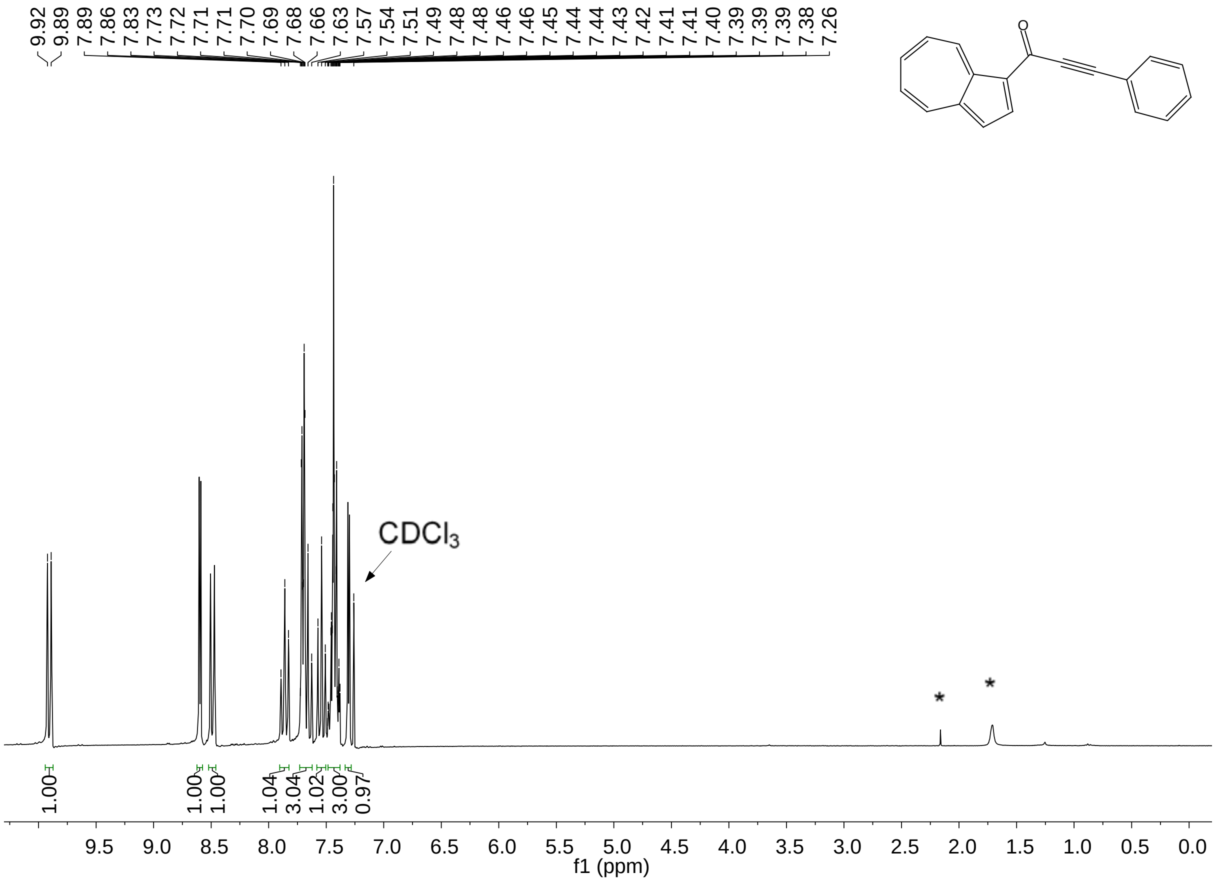

^{1}H NMR of **3a** in $CDCl_3$ at 296 K (δ in ppm). *Impurities from residual solvents.

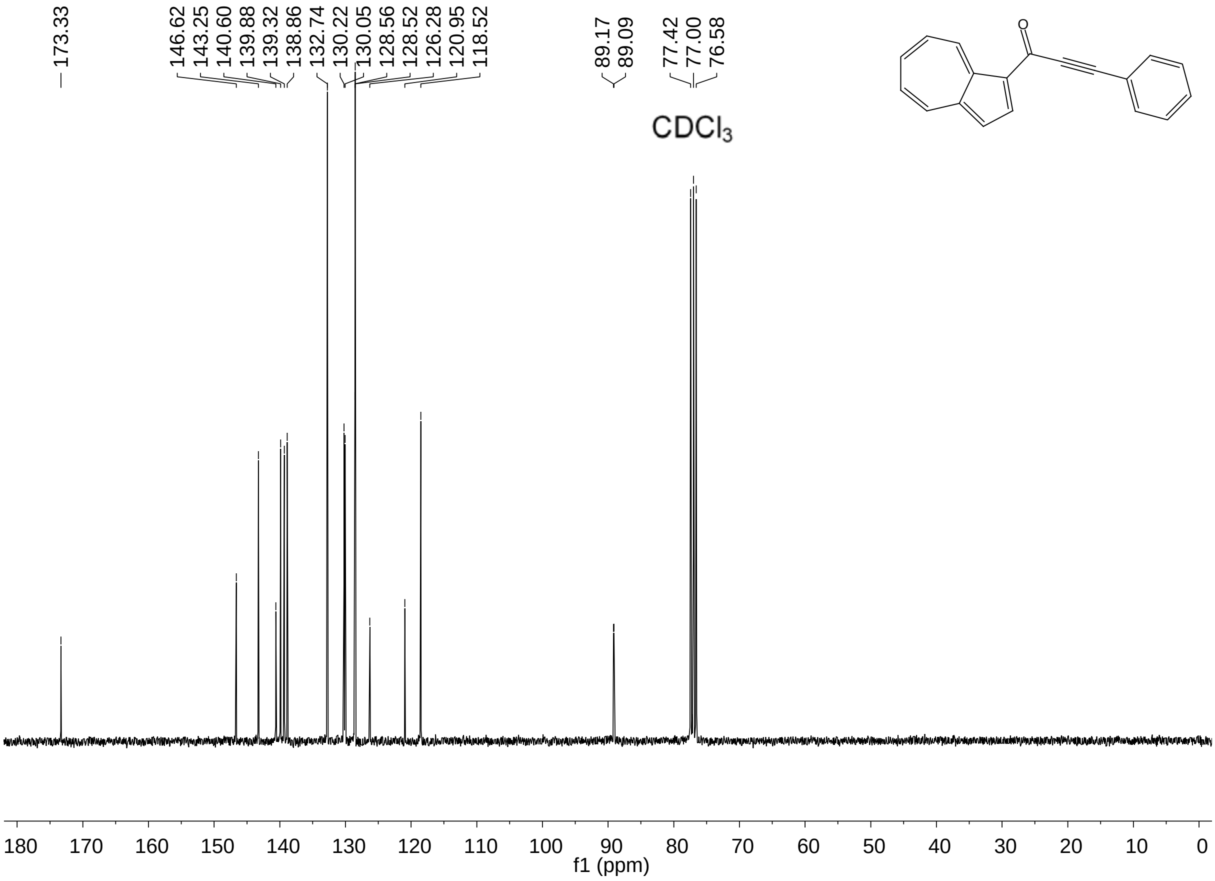

^{13}C NMR of **3a** in $CDCl_3$ at 296 K (δ in ppm).

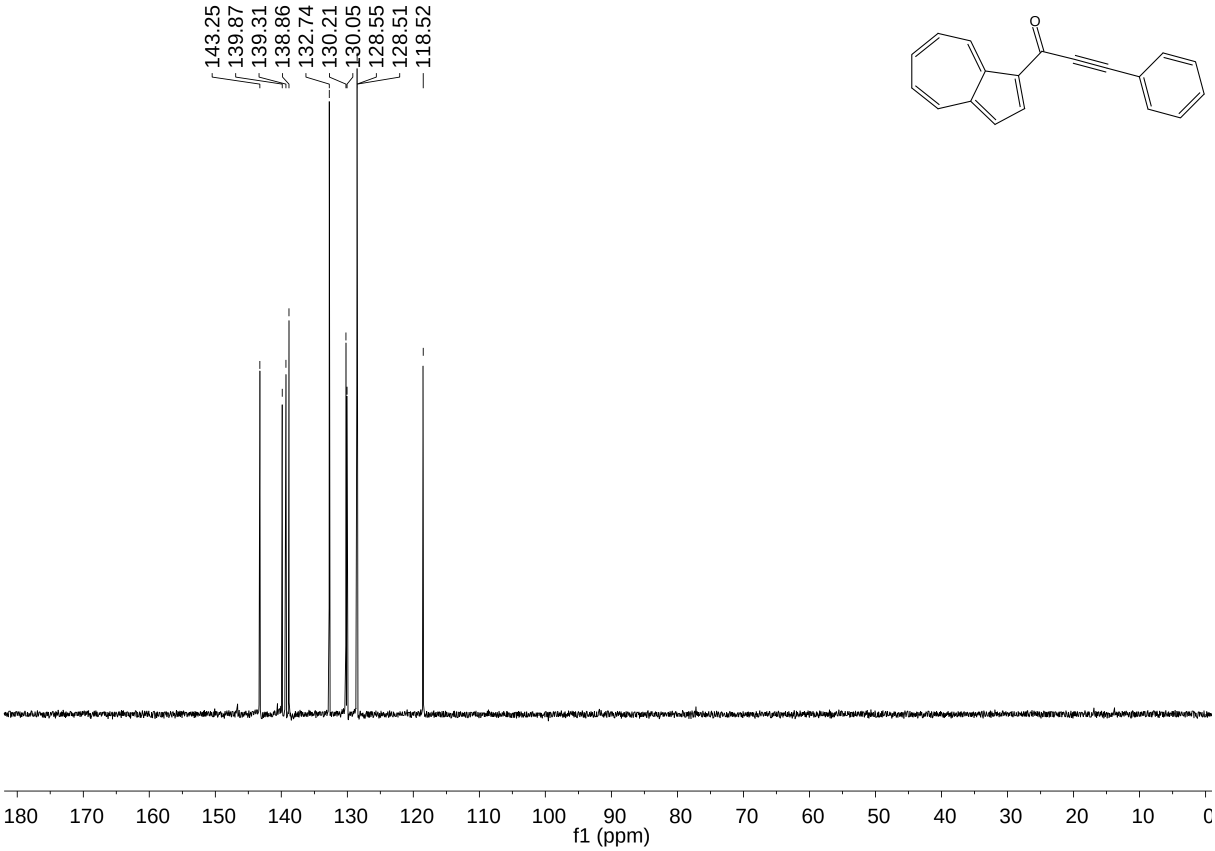

^{13}C DEPT 135-NMR of **3a** at 296 K (δ in ppm).

5.2 1-(Azulen-1-yl)hept-2-yn-1-one (3b)

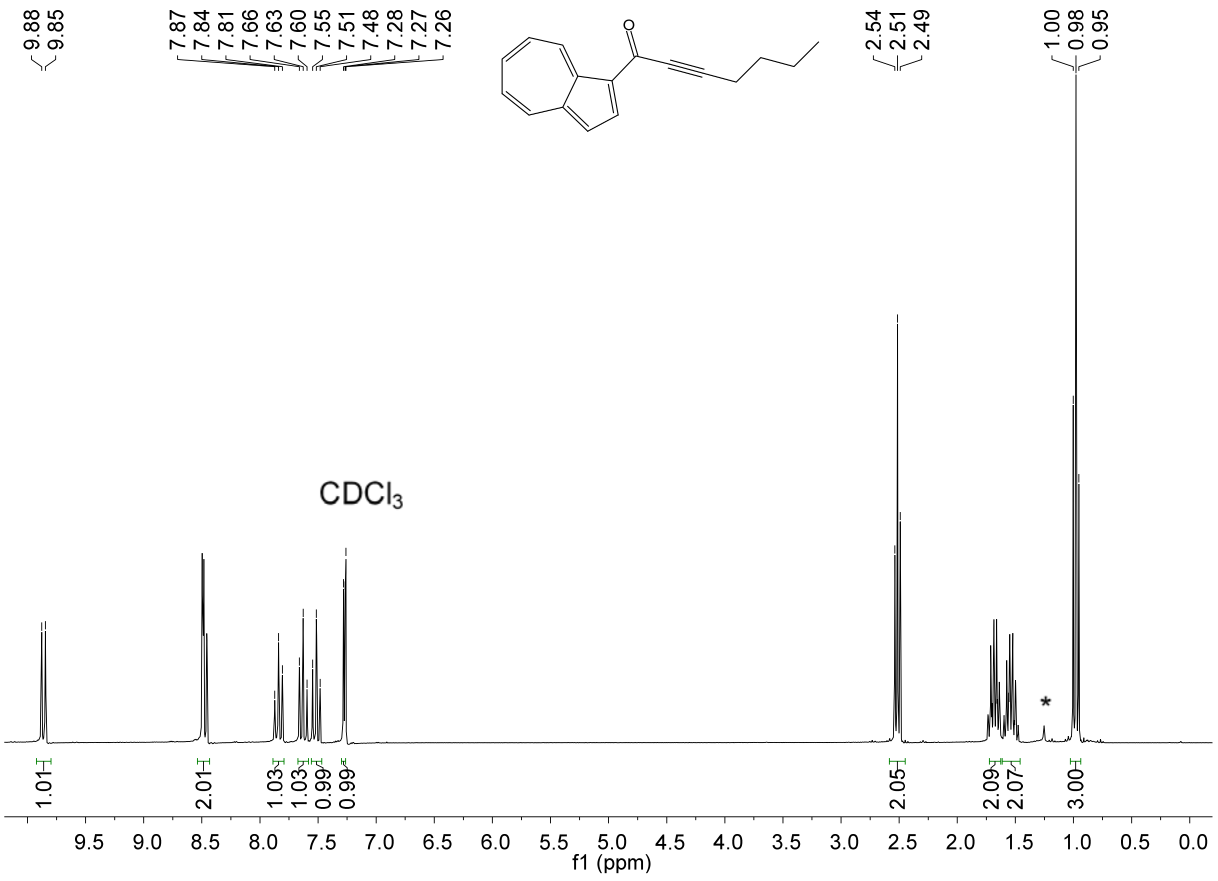

^{1}H NMR of **3b** in $CDCl_3$ at 298 K (δ in ppm). *Impurity from residual solvent.

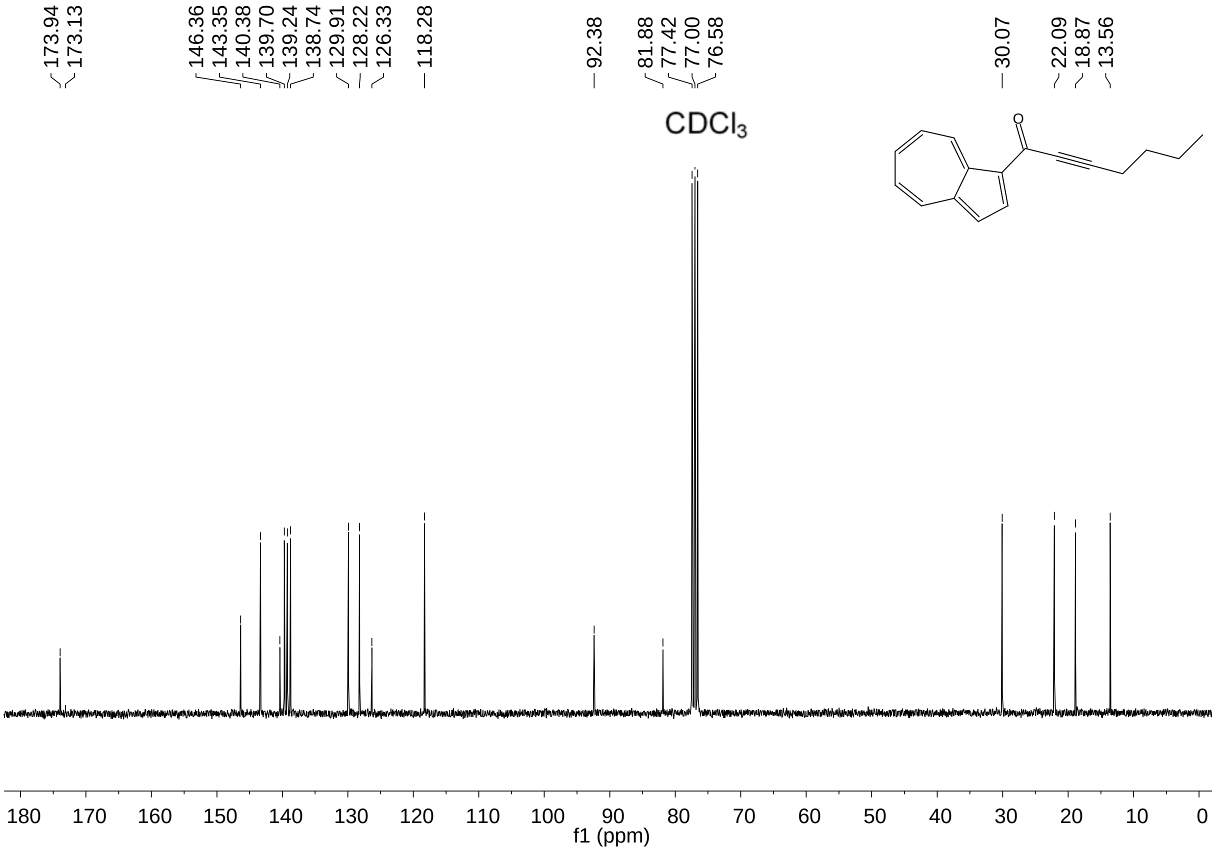

^{13}C NMR of **3b** in $CDCl_3$ at 298 K (δ in ppm).

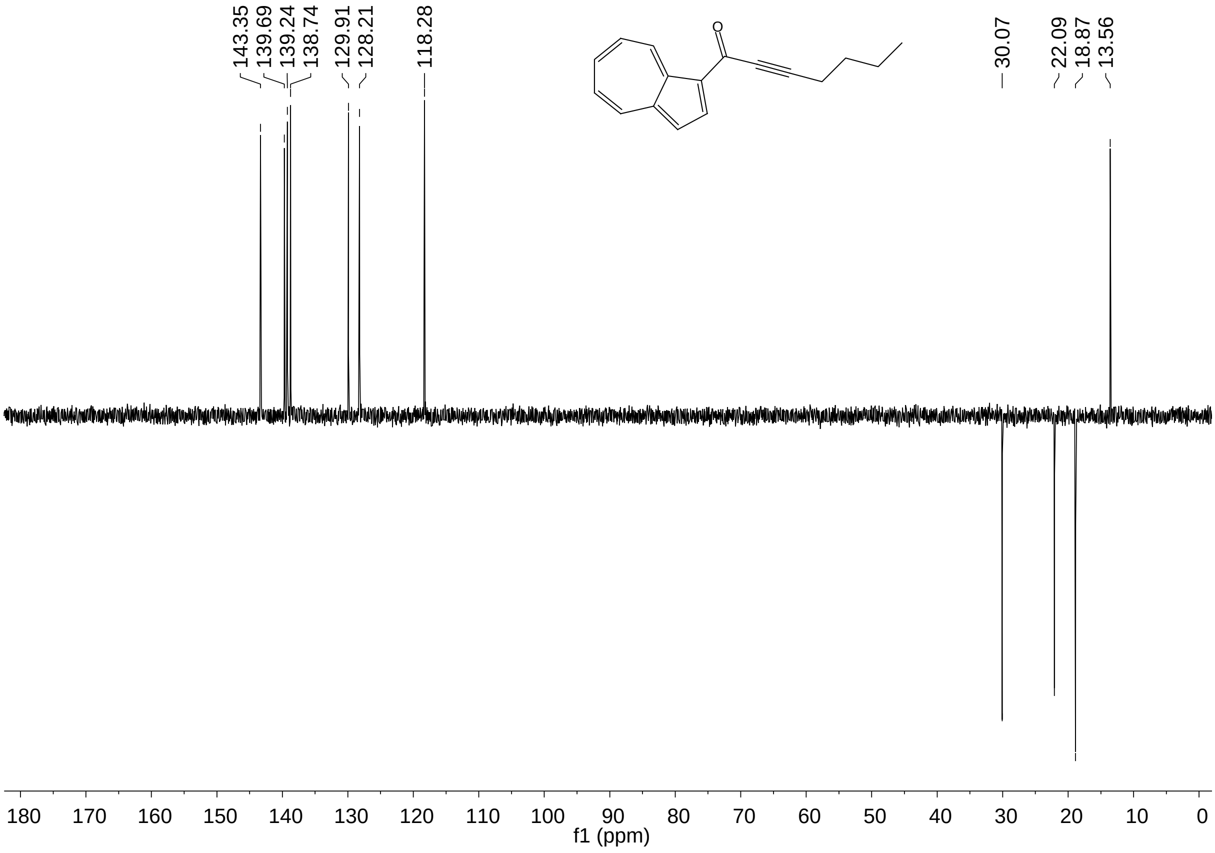

^{13}C DEPT 135-NMR of **3b** in $CDCl_3$ at 298 K (δ in ppm).

5.3 1-[3,8-Dimethyl-5-(propan-2-yl)azulen-1-yl]-3-phenylprop-2-yn-1-one (3c)

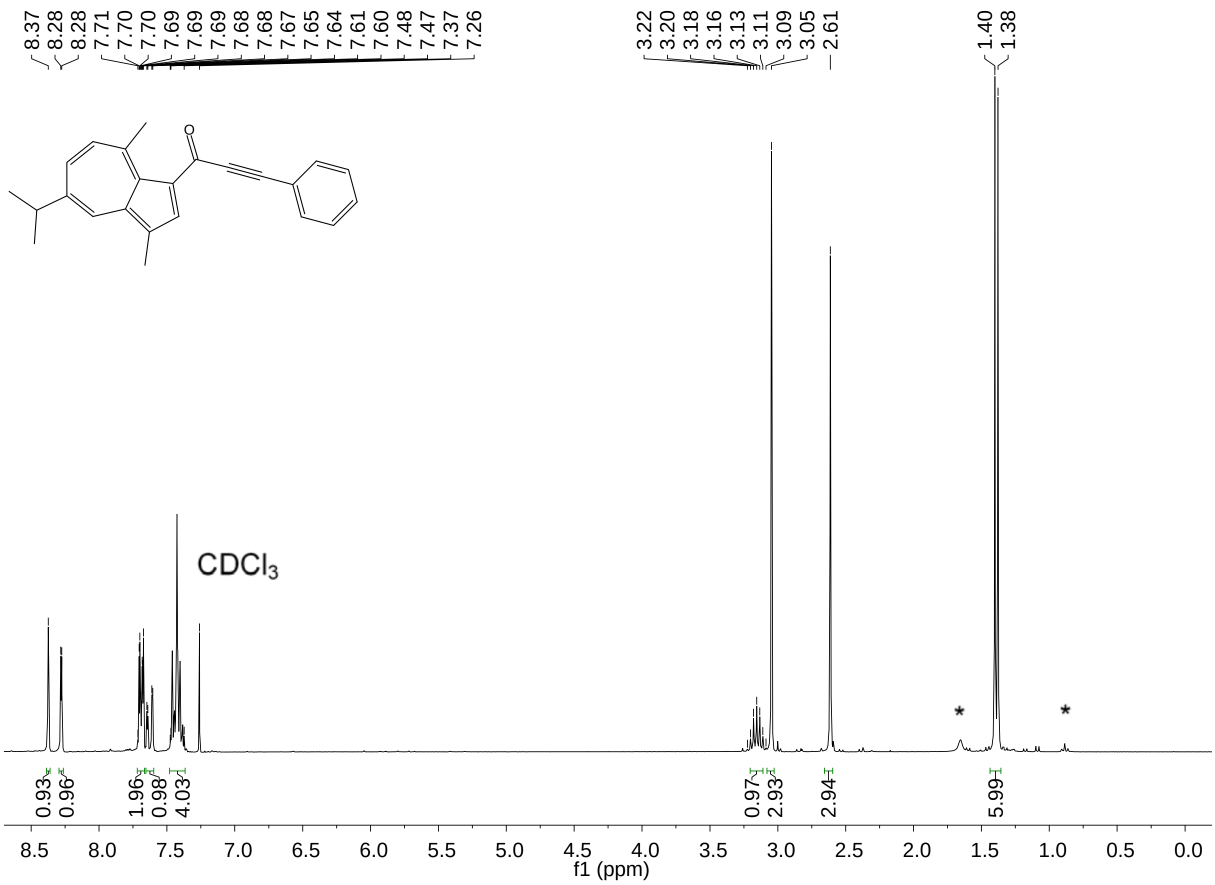

^{1}H NMR of **3c** in $CDCl_3$ at 296 K (δ in ppm). *Impurities from residual solvents.

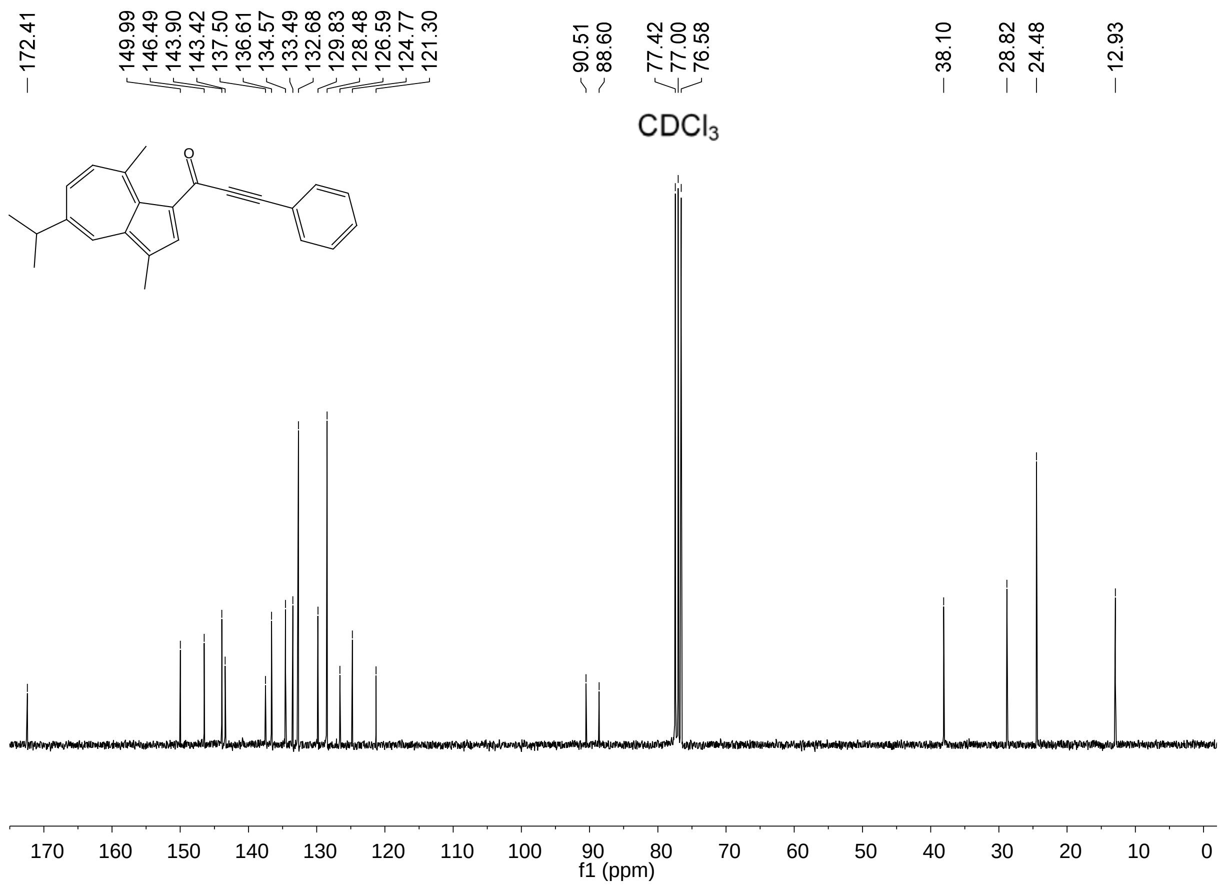

^{13}C NMR of **3c** in $CDCl_3$ at 296 K (δ in ppm).

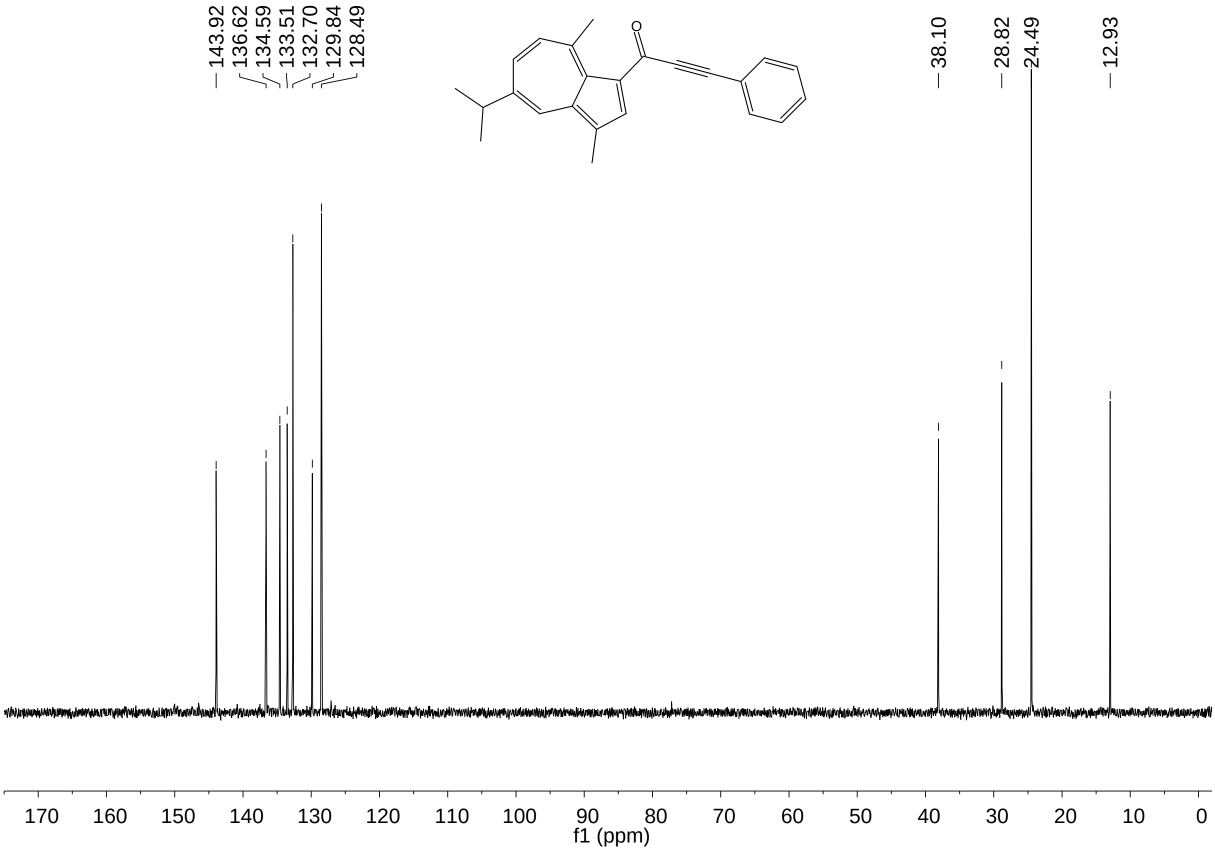

^{13}C DEPT 135-NMR of **3c** in $CDCl_3$ at 296 K (δ in ppm).

5.4 1-[3,8-Dimethyl-5-(propan-2-yl)azulen-1-yl]-3-(4-methylphenyl)prop-2-yn-1-one (3d)

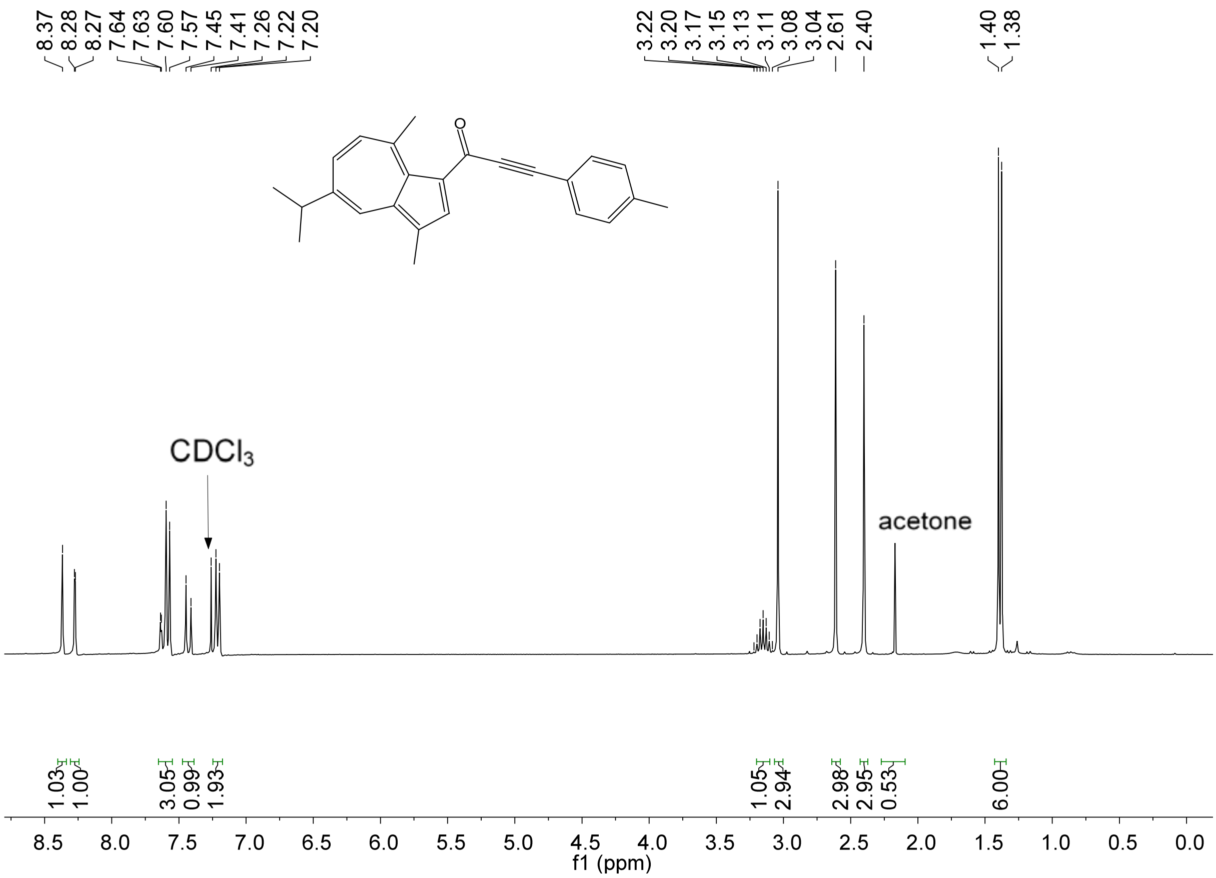

^{1}H NMR of **3d** in $CDCl_3$ at 298 K (δ in ppm).

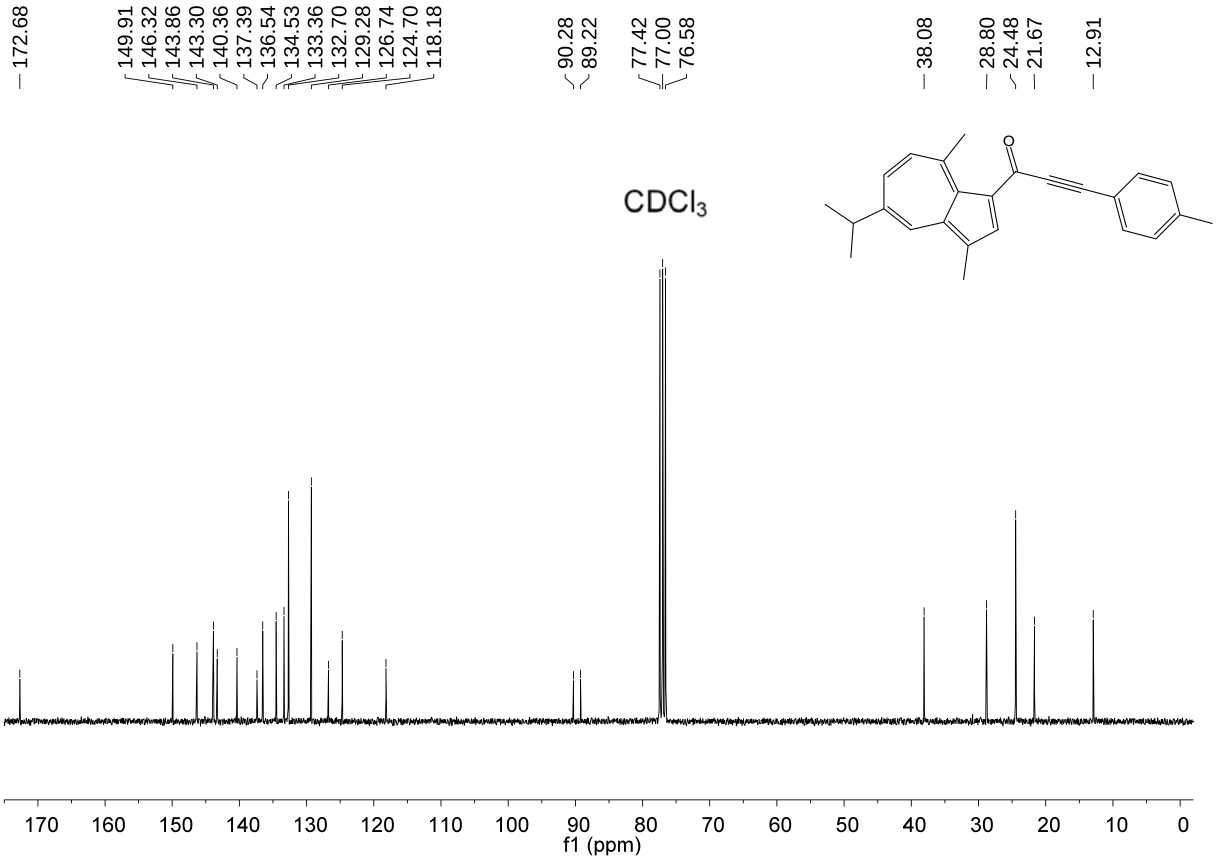

^{13}C NMR of **3d** in $CDCl_3$ at 298 K (δ in ppm).

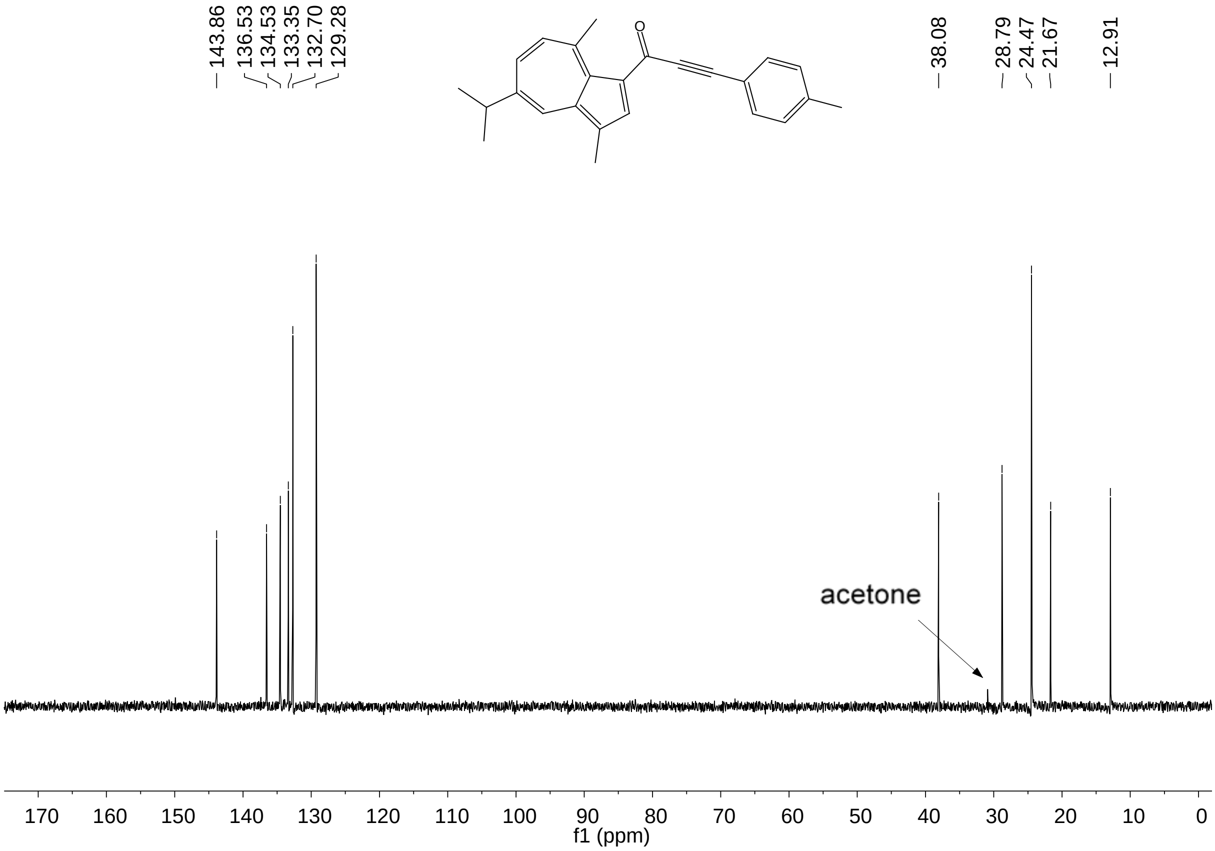

^{13}C DEPT 135-NMR of **3d** in $CDCl_3$ at 298 K (δ in ppm).

5.5 4-{3-[3,8-Dimethyl-5-(propan-2-yl)azulen-1-yl]-3-oxoprop-1-yn-1-yl}benzonitrile (3e)

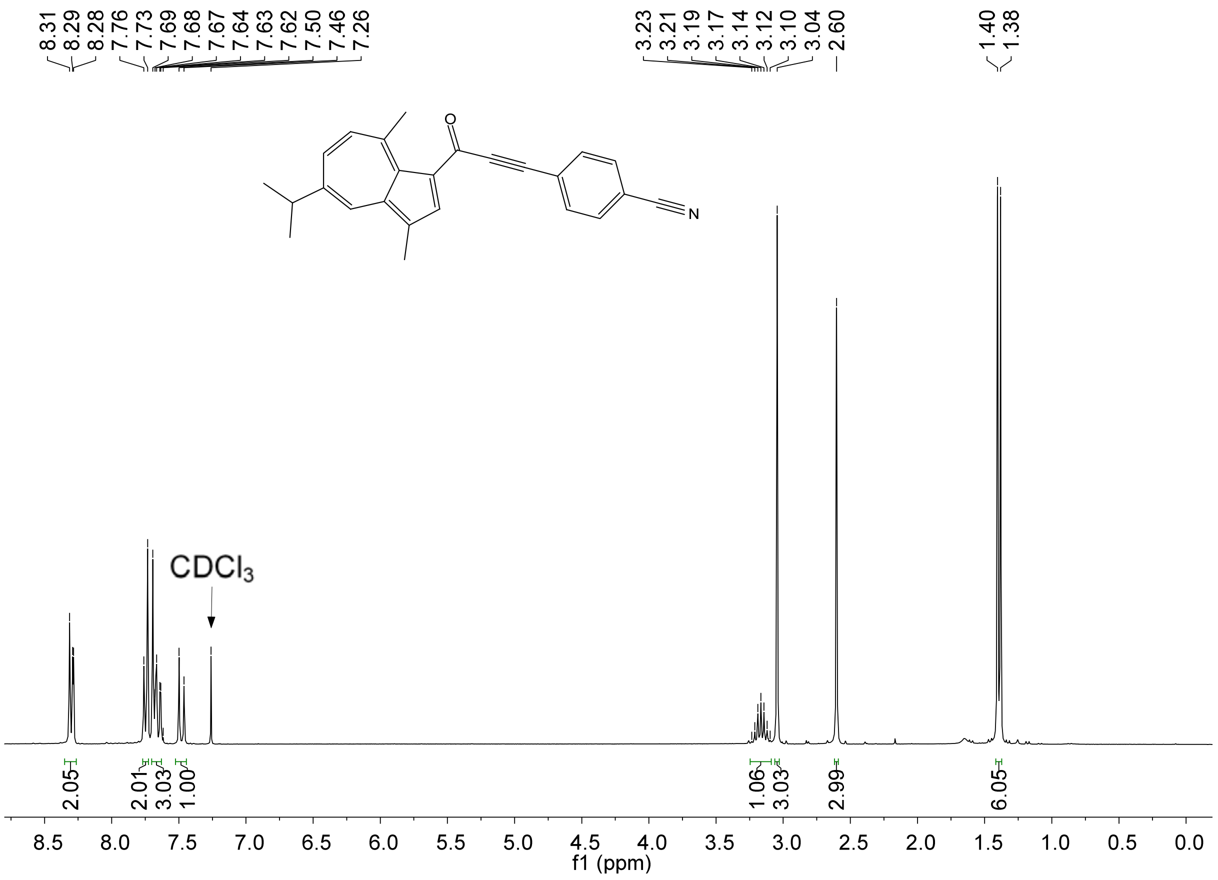

^{1}H NMR of **3e** in $CDCl_3$ at 298 K (δ in ppm).

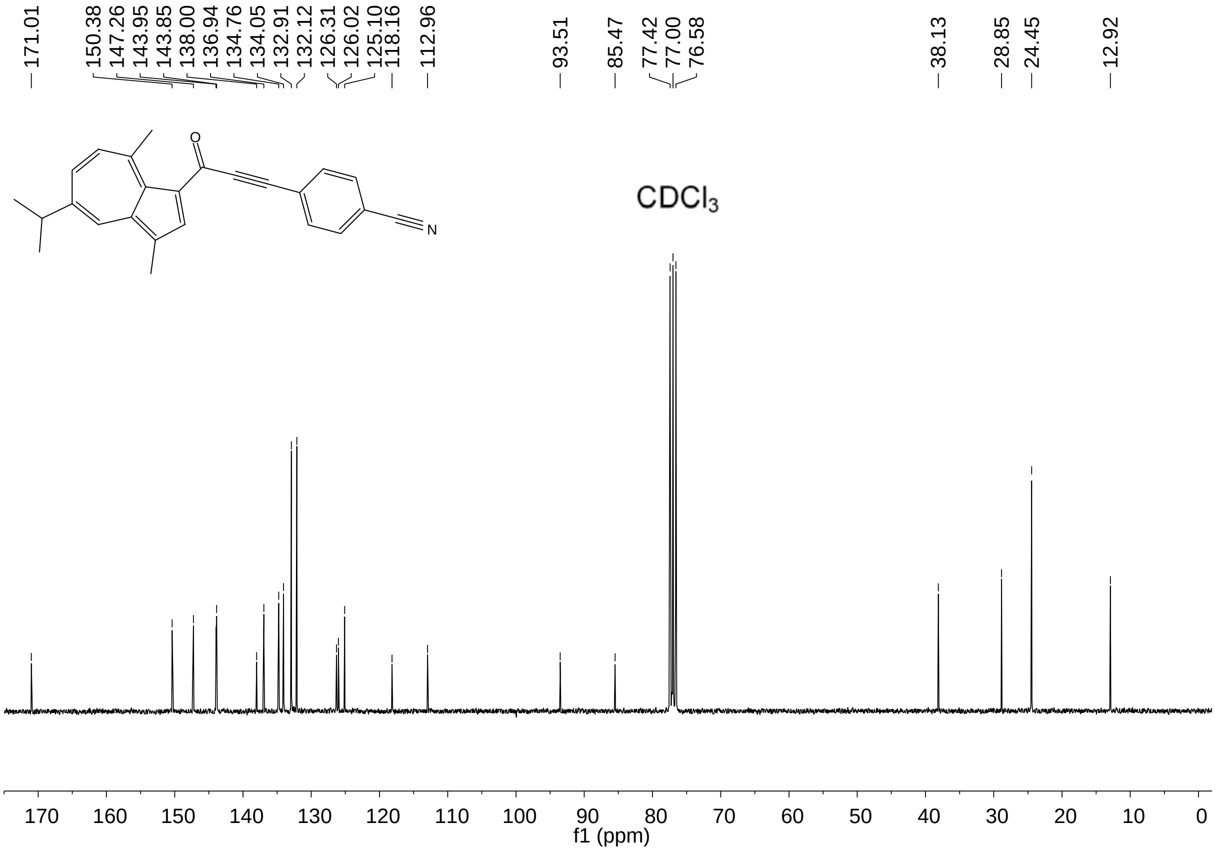

^{13}C NMR of **3e** in $CDCl_3$ at 298 K (δ in ppm).

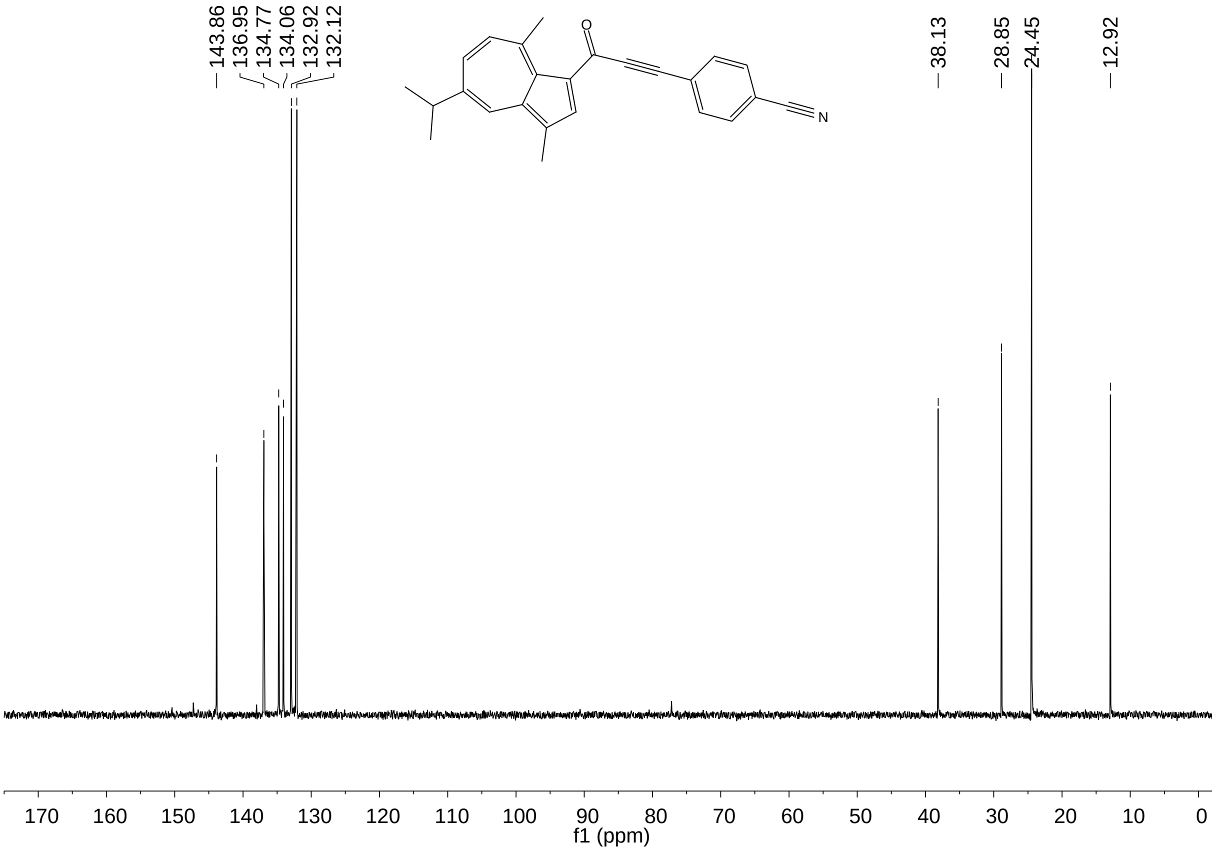

^{13}C DEPT 135-NMR of **3e** in $CDCl_3$ at 298 K (δ in ppm).

5.6 1-[3,8-Dimethyl-5-(propan-2-yl)azulen-1-yl]-3-(4-nitrophenyl)prop-2-yn-1-one (3f)

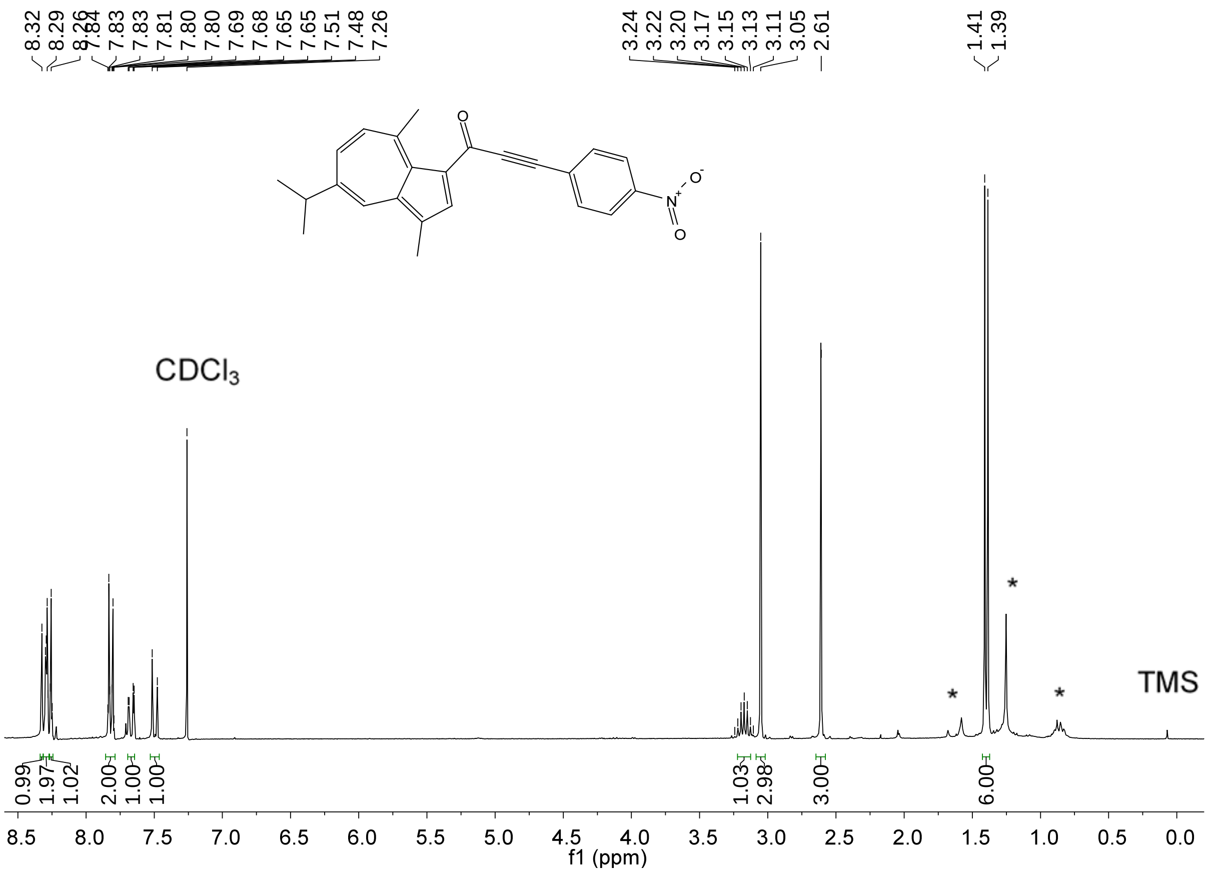

^{1}H NMR of **3f** in $CDCl_3$ at 295 K (δ in ppm). *Impurities from residual solvents.

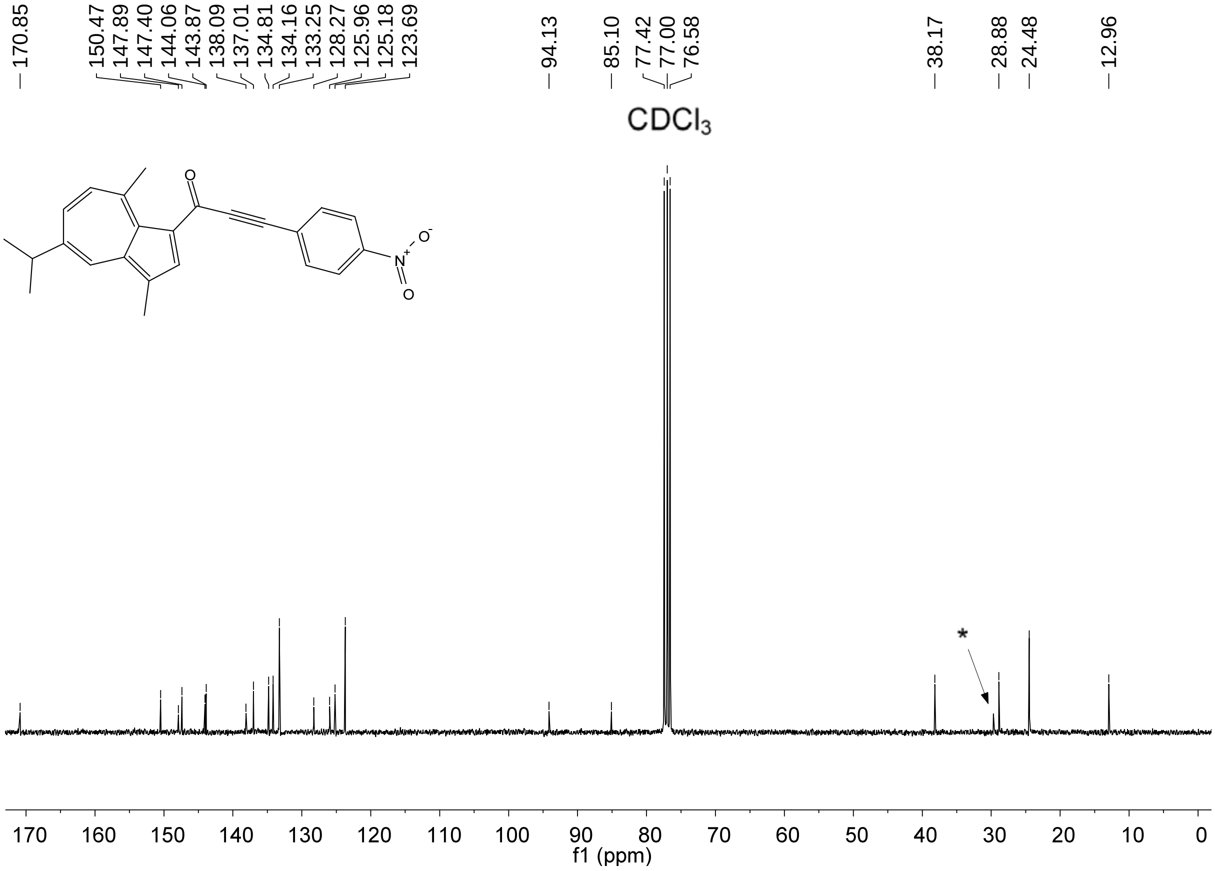

^{13}C NMR of **3f** in $CDCl_3$ at 295 K (δ in ppm). *Impurity from residual solvents.

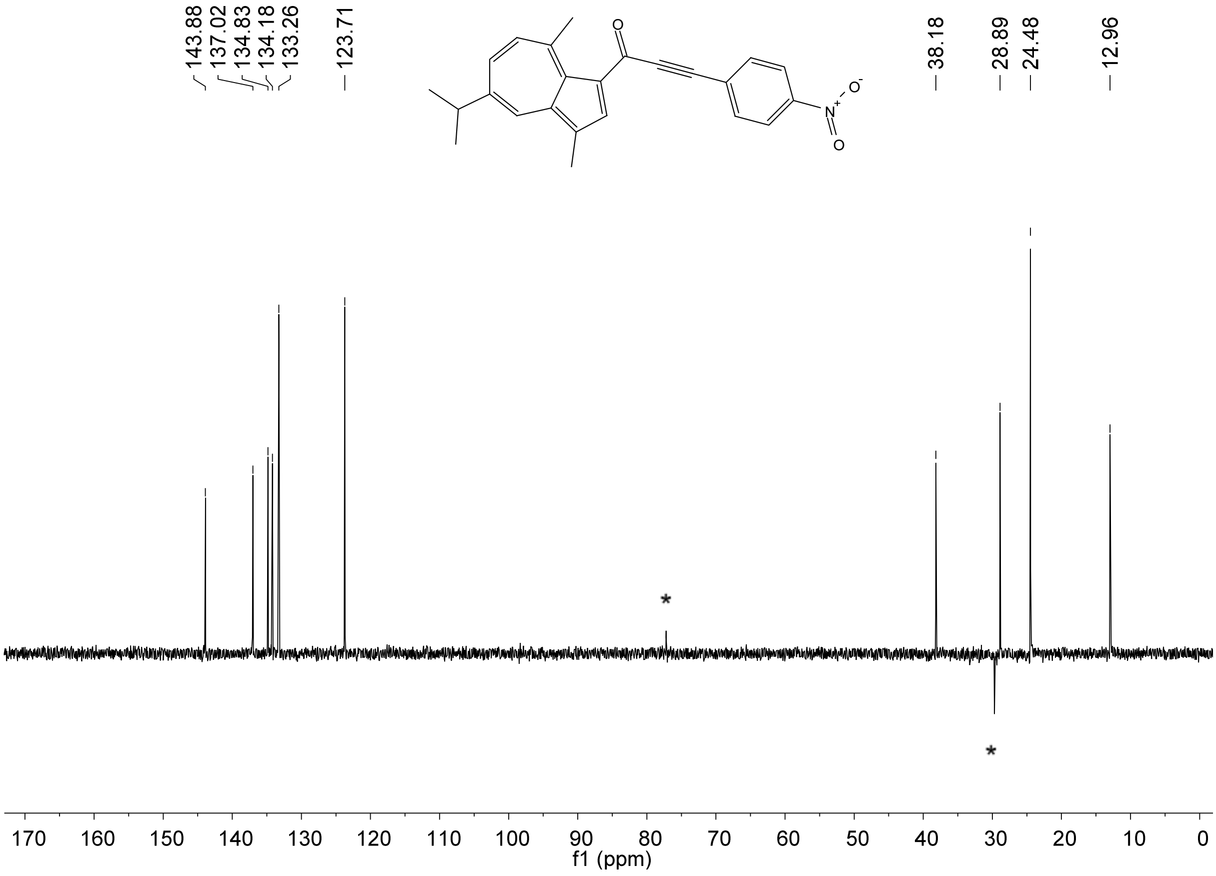

^{13}C DEPT 135-NMR of **3f** in $CDCl_3$ at 295 (δ in ppm). *Impurities from residual solvents.

5.7 1-[3,8-Dimethyl-5-(propan-2-yl)azulen-1-yl]-3-(3-fluorophenyl)prop-2-yn-1-one (3g)

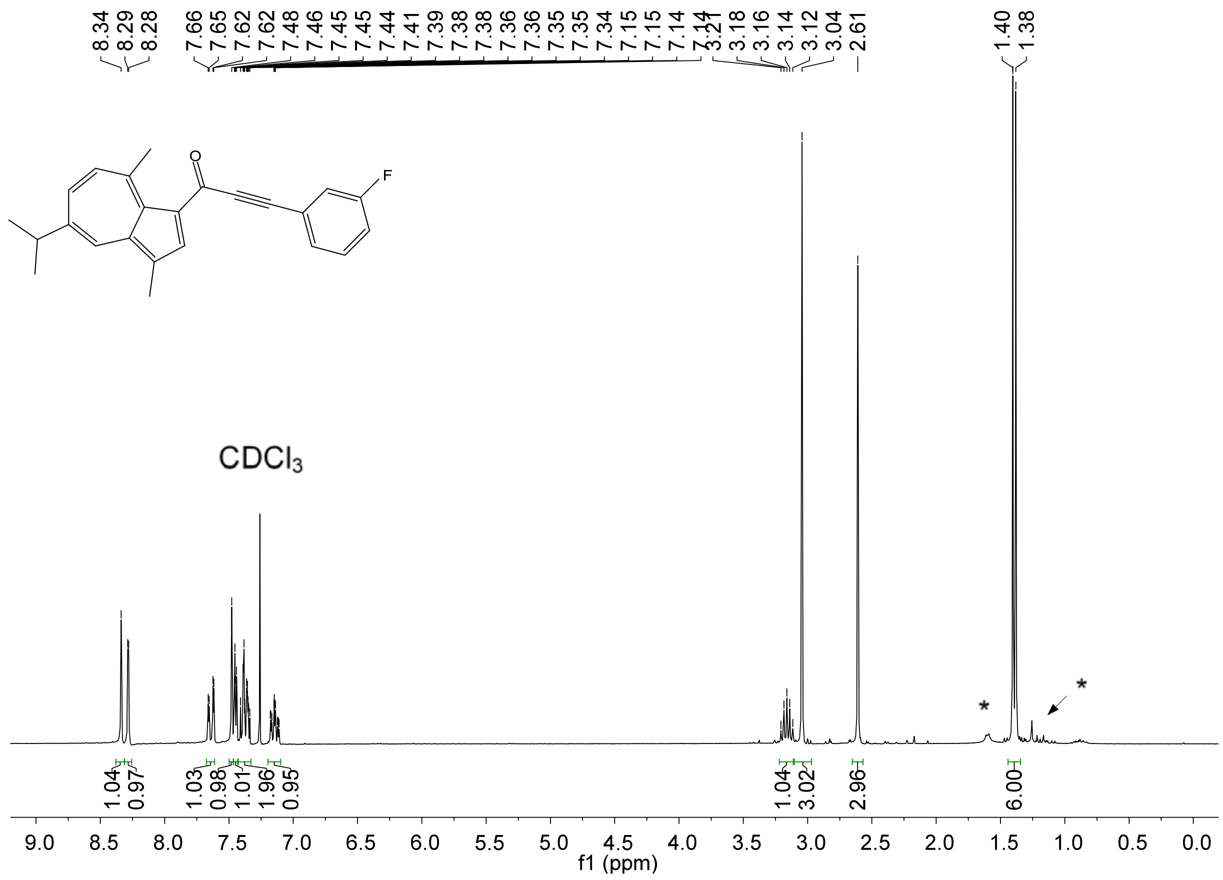

^{1}H NMR of **3g** in $CDCl_3$ at 298 K (δ in ppm). *Impurities from residual solvents.

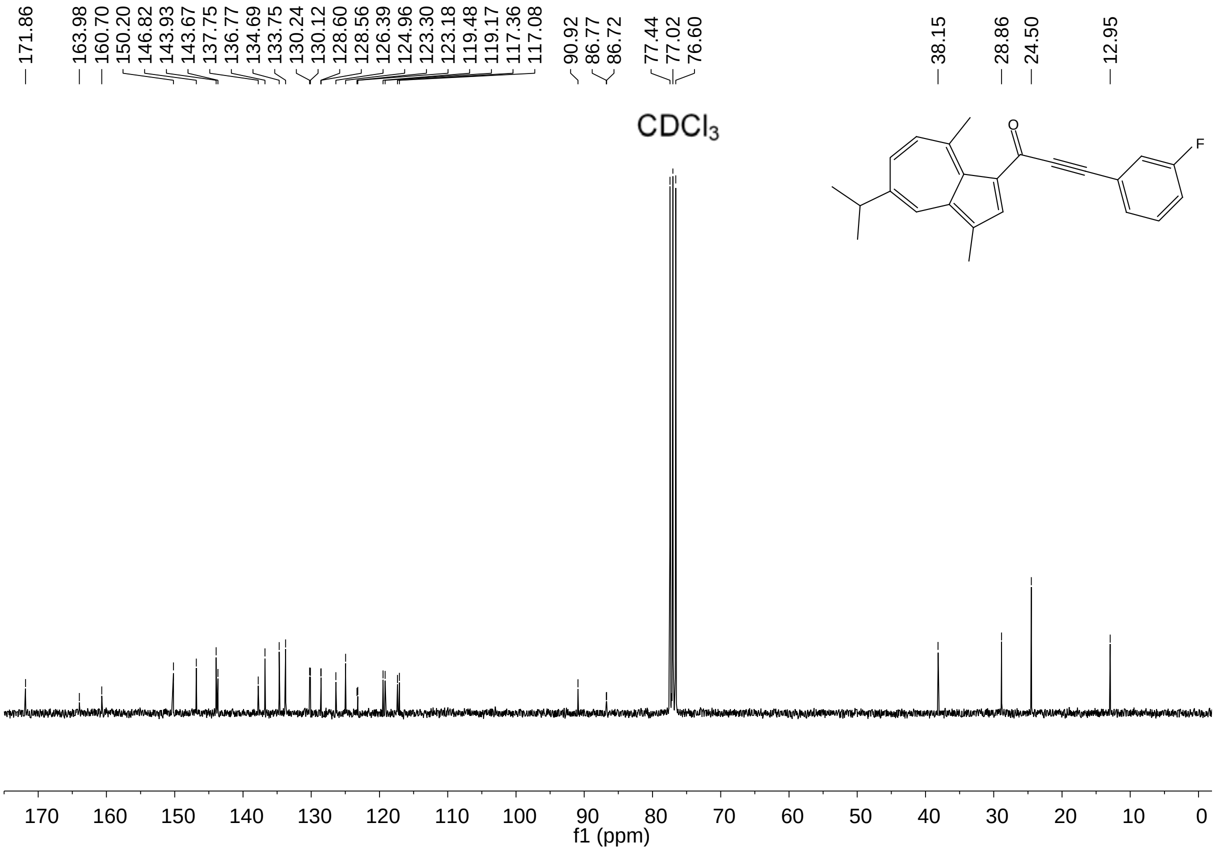

^{13}C NMR of **3g** in $CDCl_3$ at 298 K (δ in ppm).

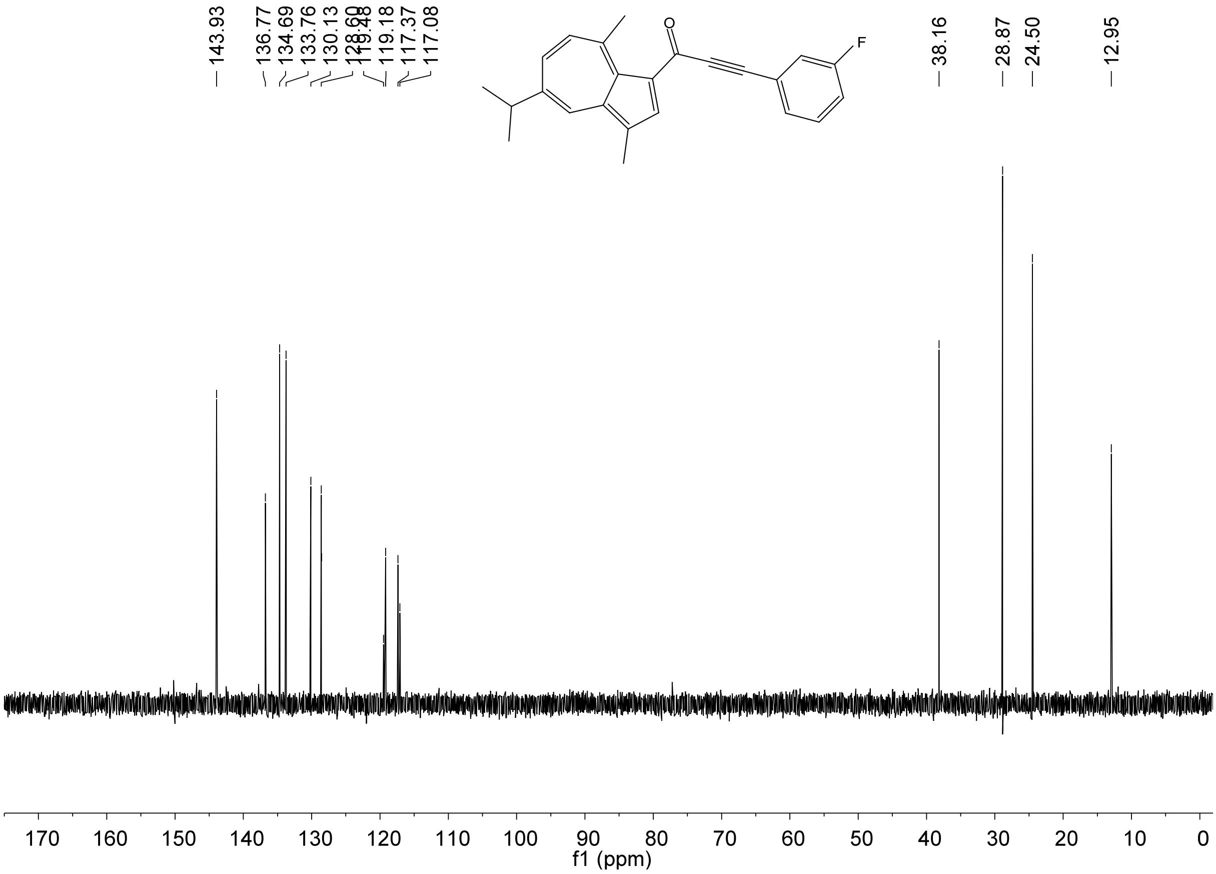

^{13}C DEPT 135-NMR of **3g** in $CDCl_3$ at 298 K (δ in ppm).

5.8 3-(3,5-Dimethoxyphenyl)-1-[3,8-dimethyl-5-(propan-2-yl)azulen-1-yl]prop-2-yn-1-one (3h)

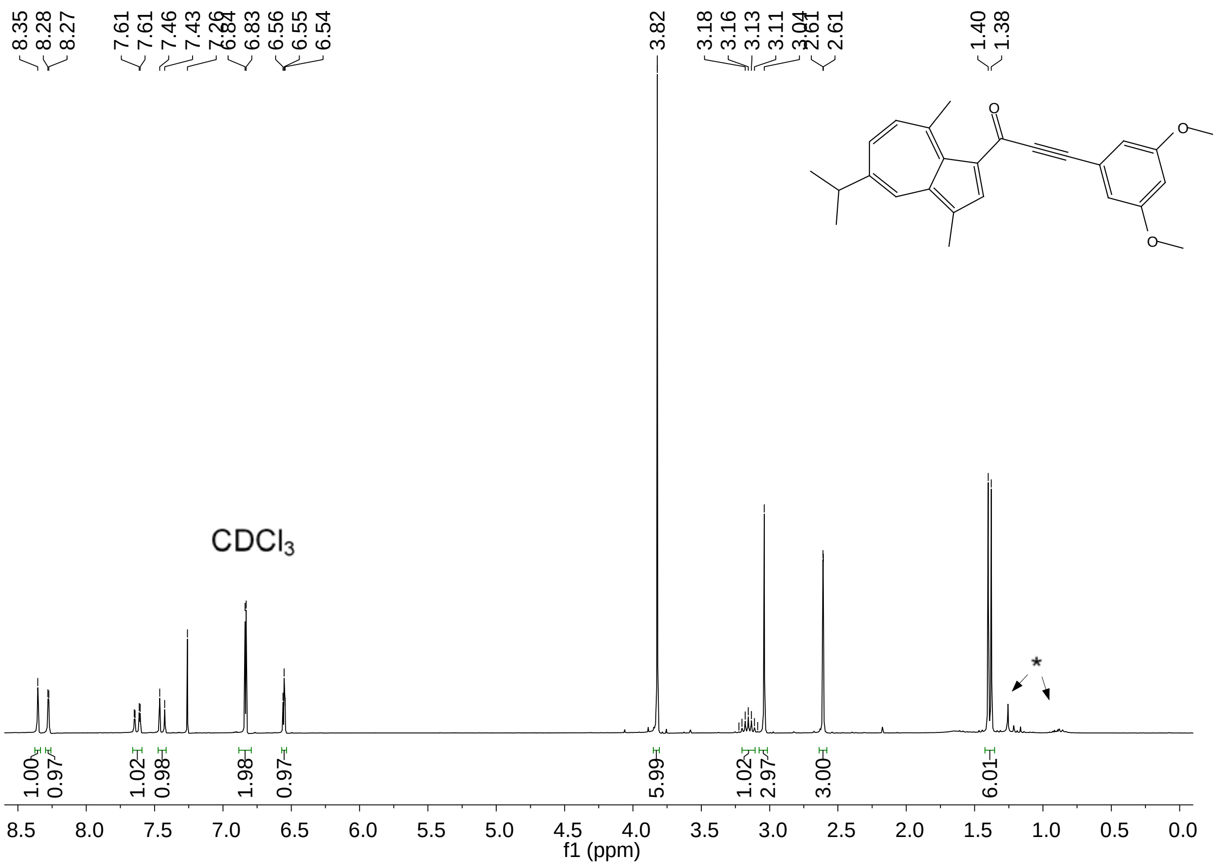

^{1}H NMR of **3h** in $CDCl_3$ at 298 K (δ in ppm). *Impurities from residual solvents.

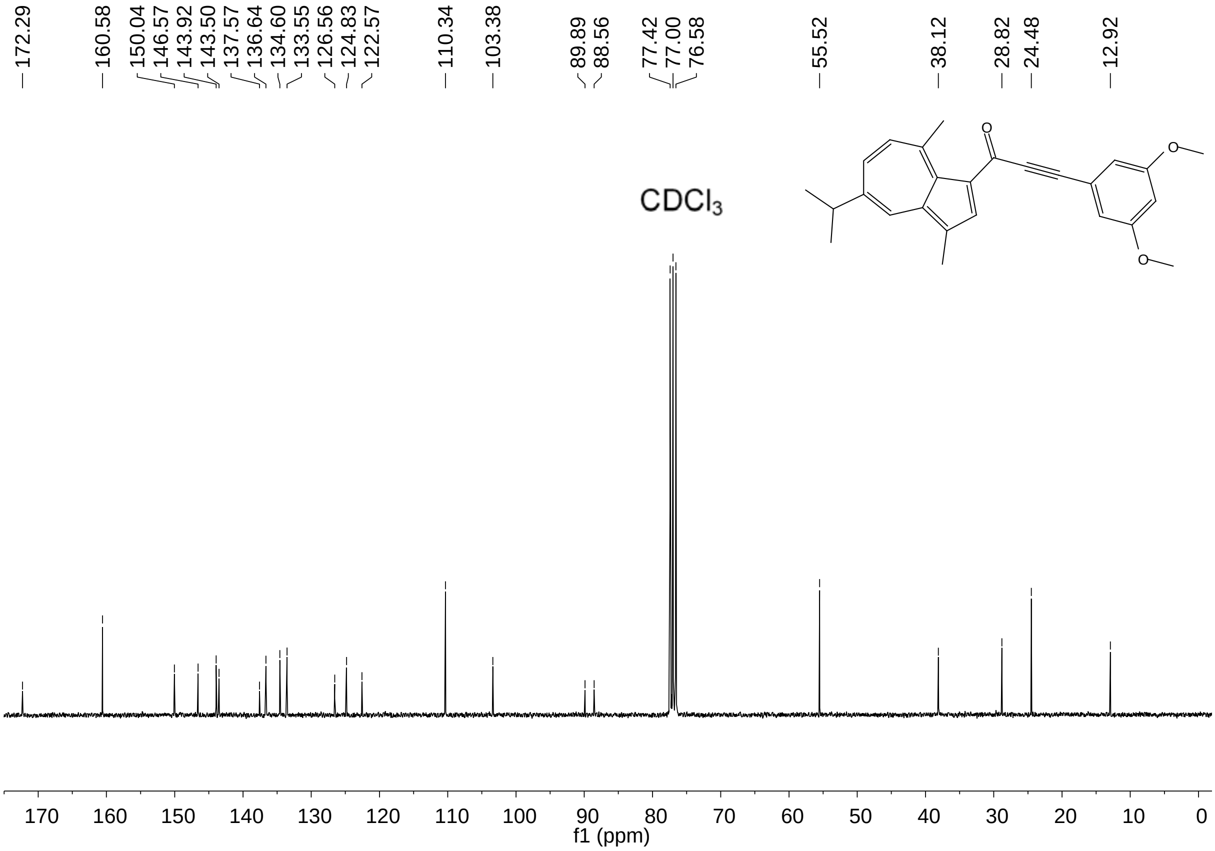

^{13}C NMR of **3h** in $CDCl_3$ at 298 K (δ in ppm).

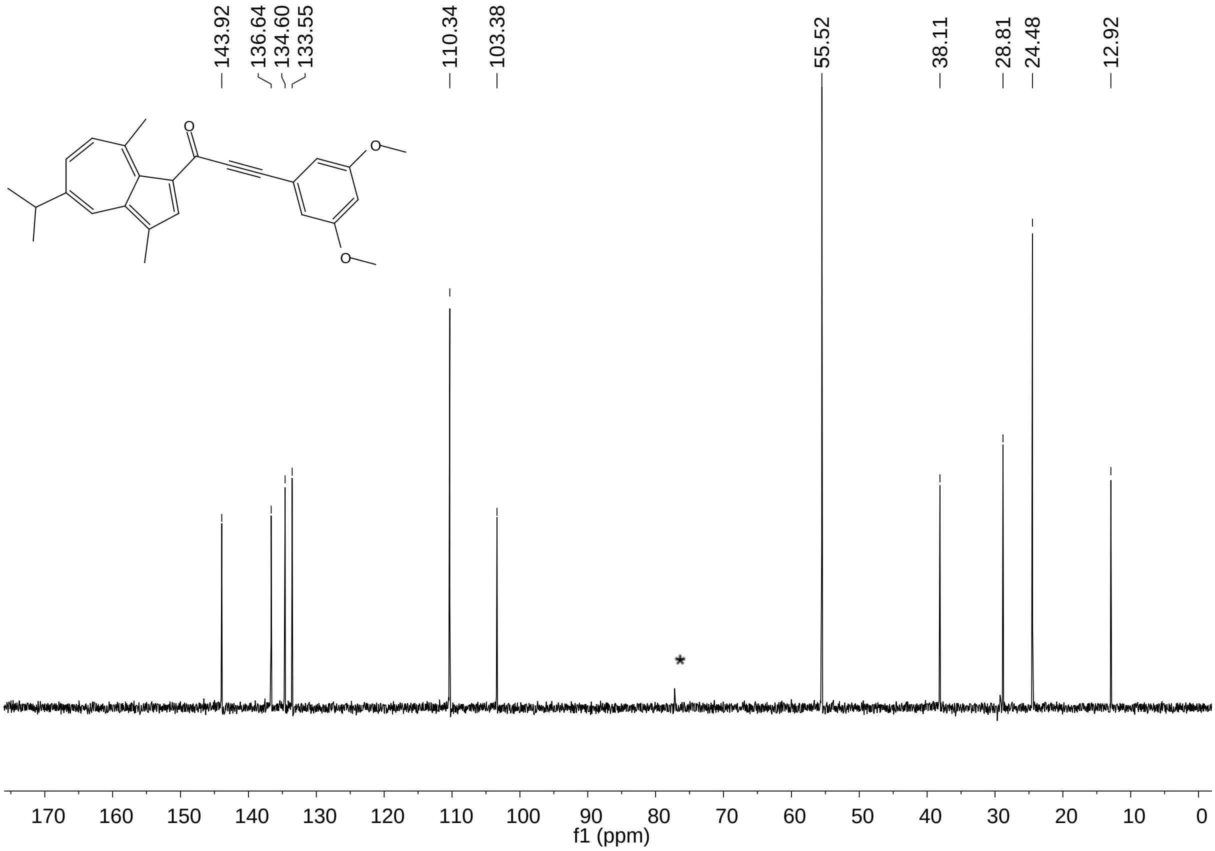

^{13}C DEPT 135-NMR of **3h** in $CDCl_3$ at 298 K (δ in ppm). *Impurities from residual solvents.

5.9 1-[3,8-Dimethyl-5-(propan-2-yl)azulen-1-yl]-3-(thiophen-2-yl)prop-2-yn-1-one (3i)

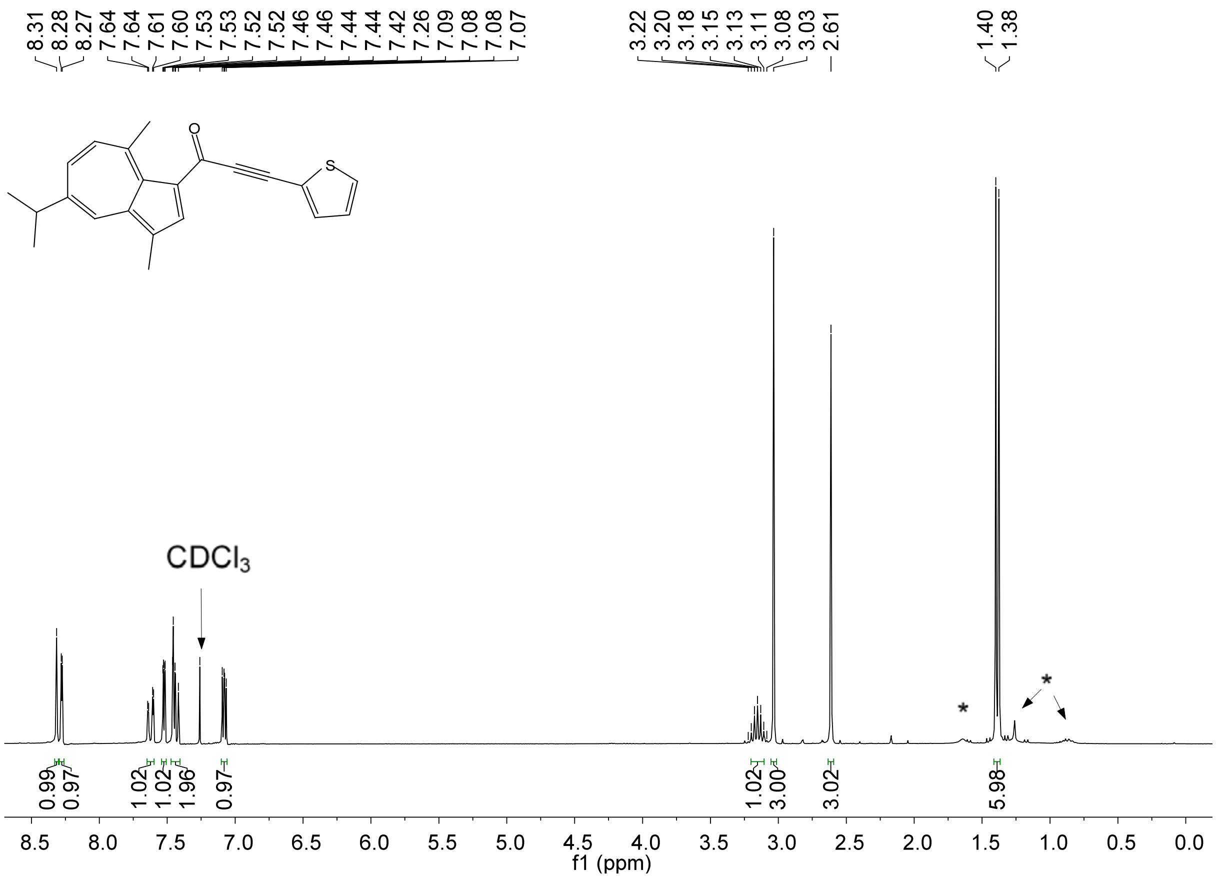

^{1}H NMR of **3i** in $CDCl_3$ at 298 K (δ in ppm). *Impurities from residual solvents.

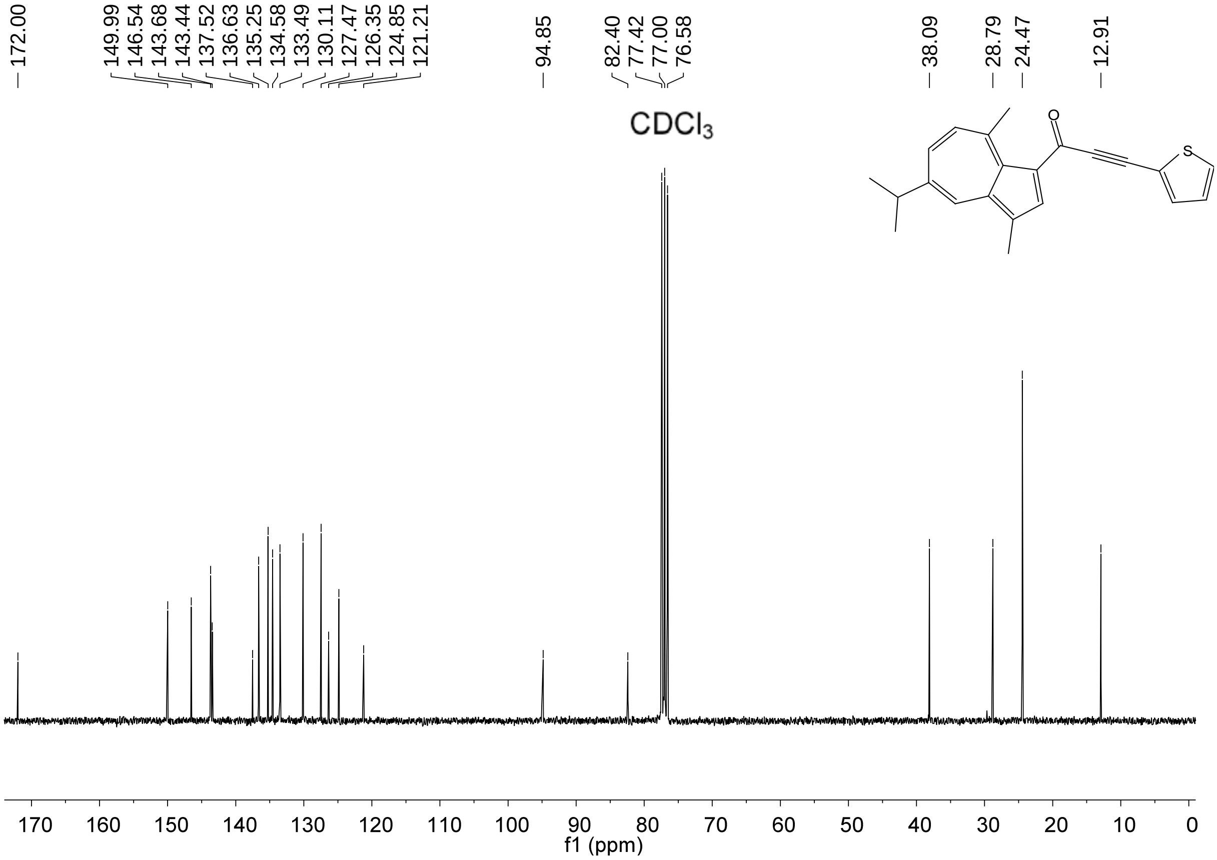

^{13}C NMR of **3i** in $CDCl_3$ at 298 K (δ in ppm).

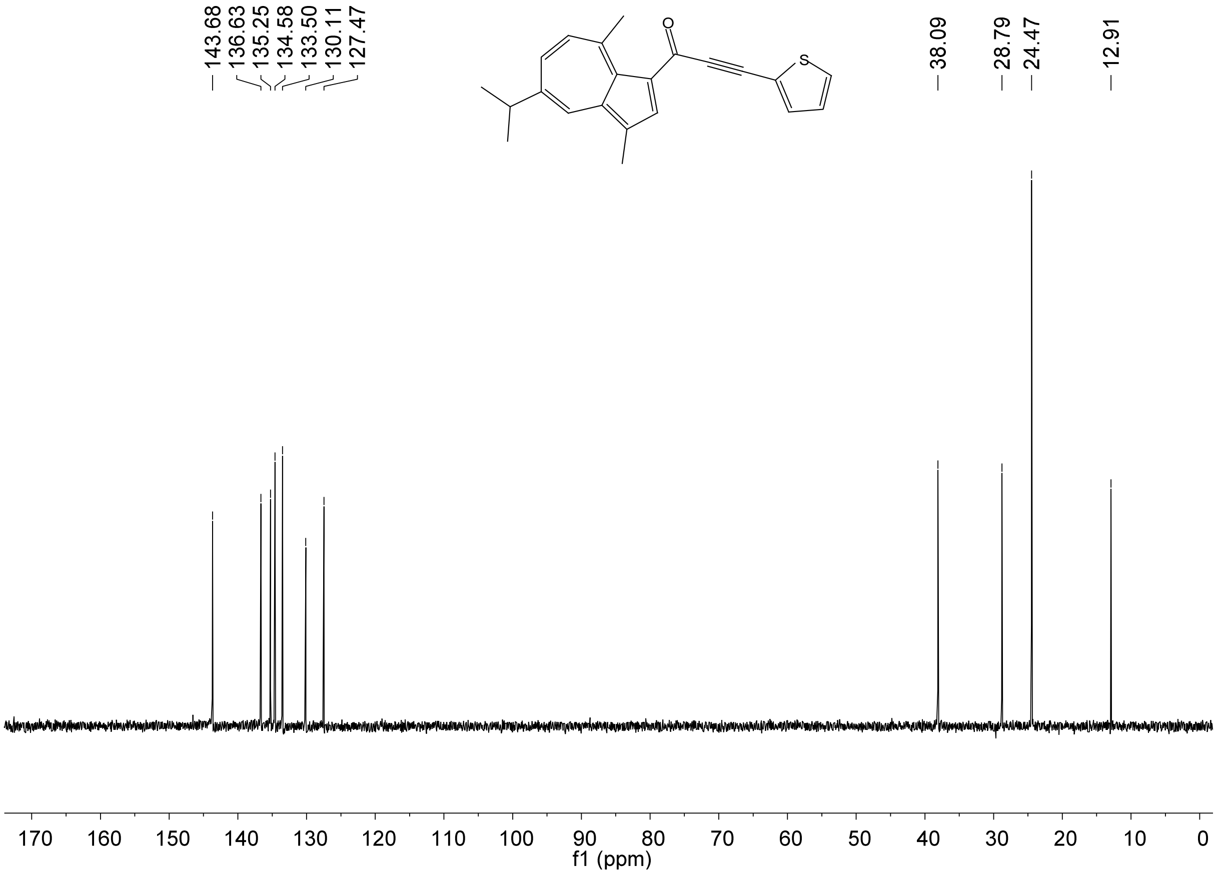

^{13}C DEPT 135-NMR of **3i** in $CDCl_3$ at 298 K (δ in ppm).

5.10 1-[3,8-Dimethyl-5-(propan-2-yl)azulen-1-yl]-3-(pyridin-3-yl)prop-2-yn-1-one (3j)

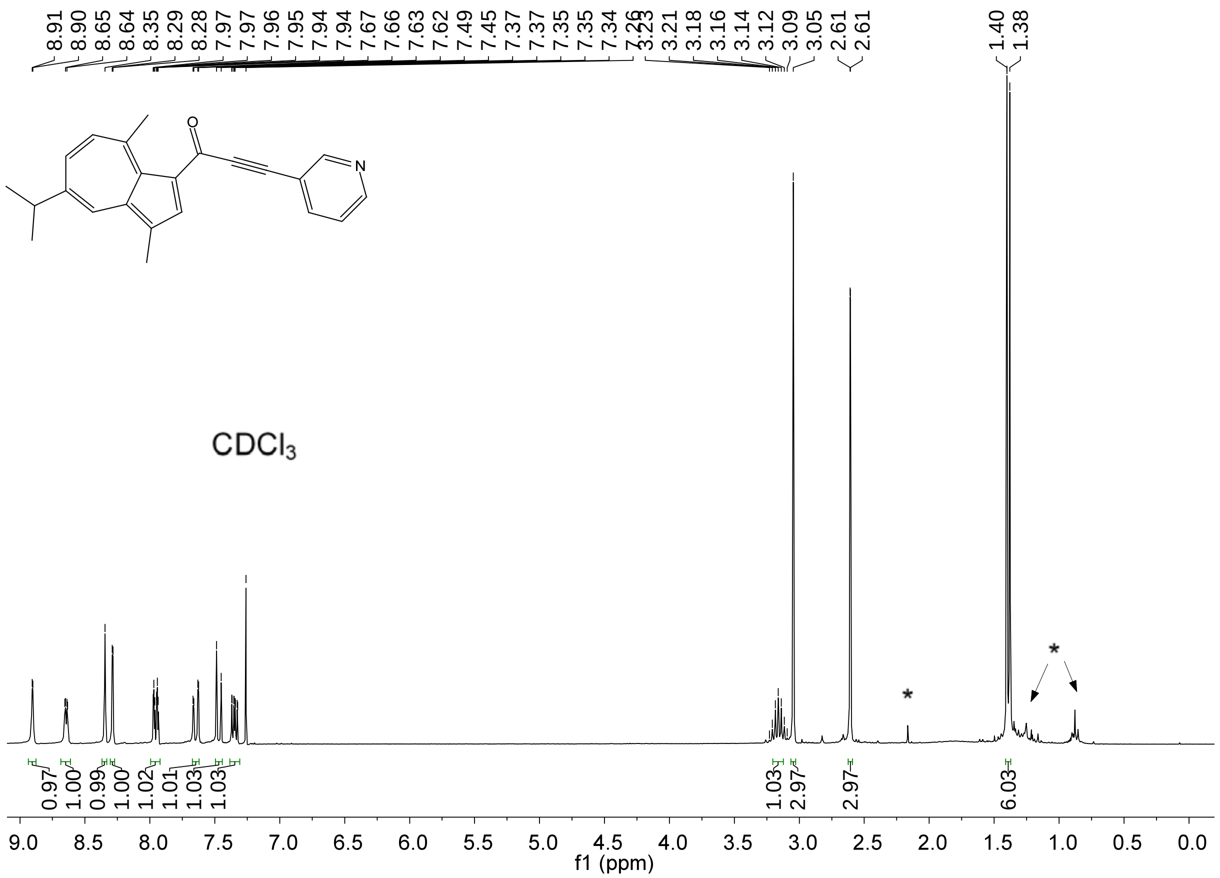

^{1}H NMR of **3j** in $CDCl_3$ at 298 K (δ in ppm). *Impurities from residual solvents.

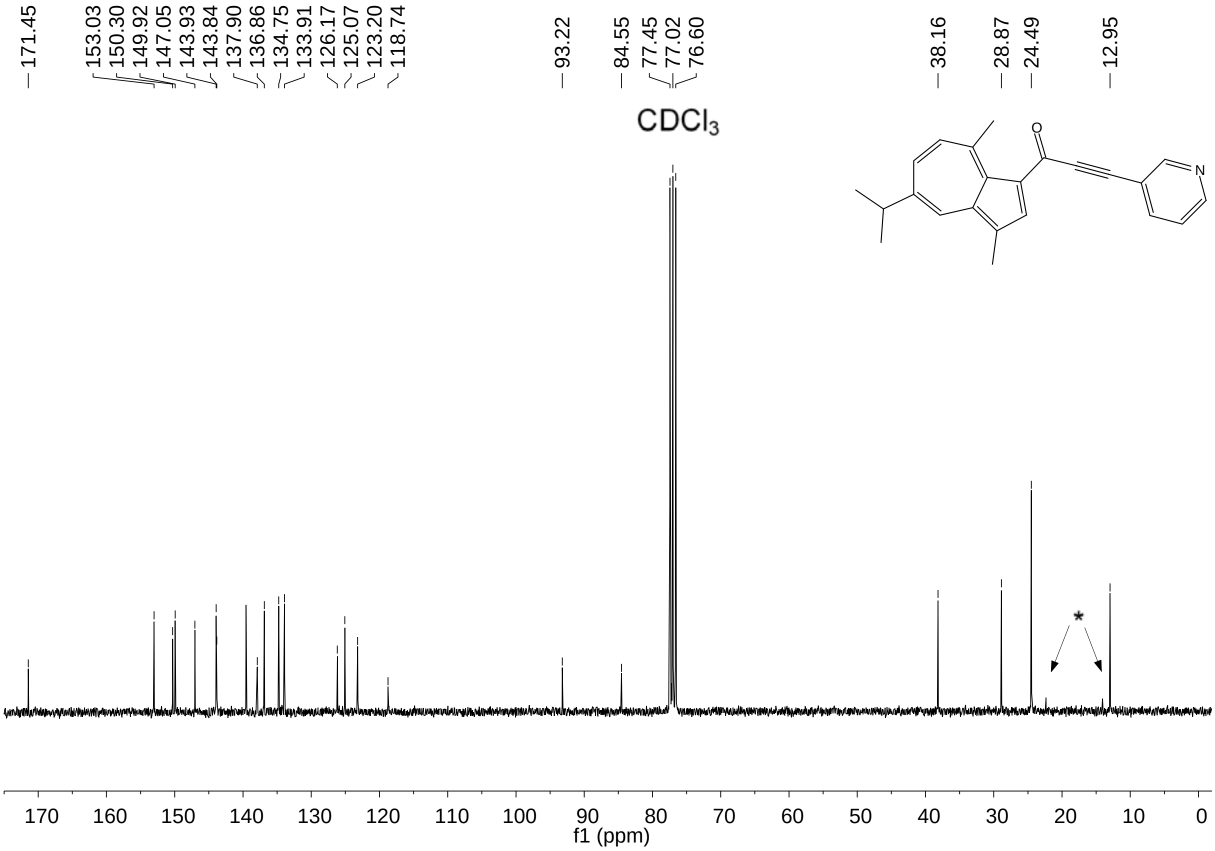

^{13}C NMR of **3j** in $CDCl_3$ at 298 K (δ in ppm). *Impurities from residual solvents.

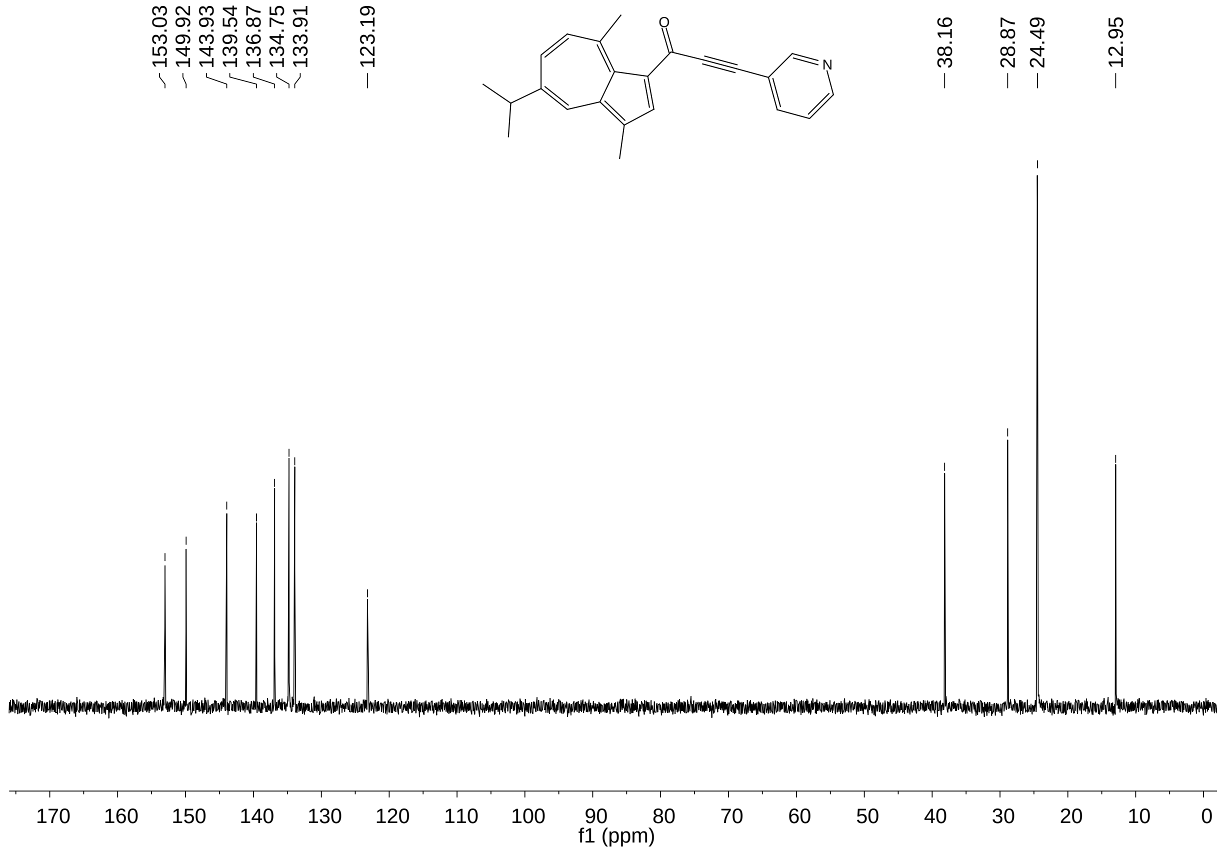

^{13}C DEPT 135-NMR of **3j** in $CDCl_3$ at 298 (δ in ppm).

5.11 1-[3,8-Dimethyl-5-(propan-2-yl)azulen-1-yl]hept-2-yn-1-one (3k)

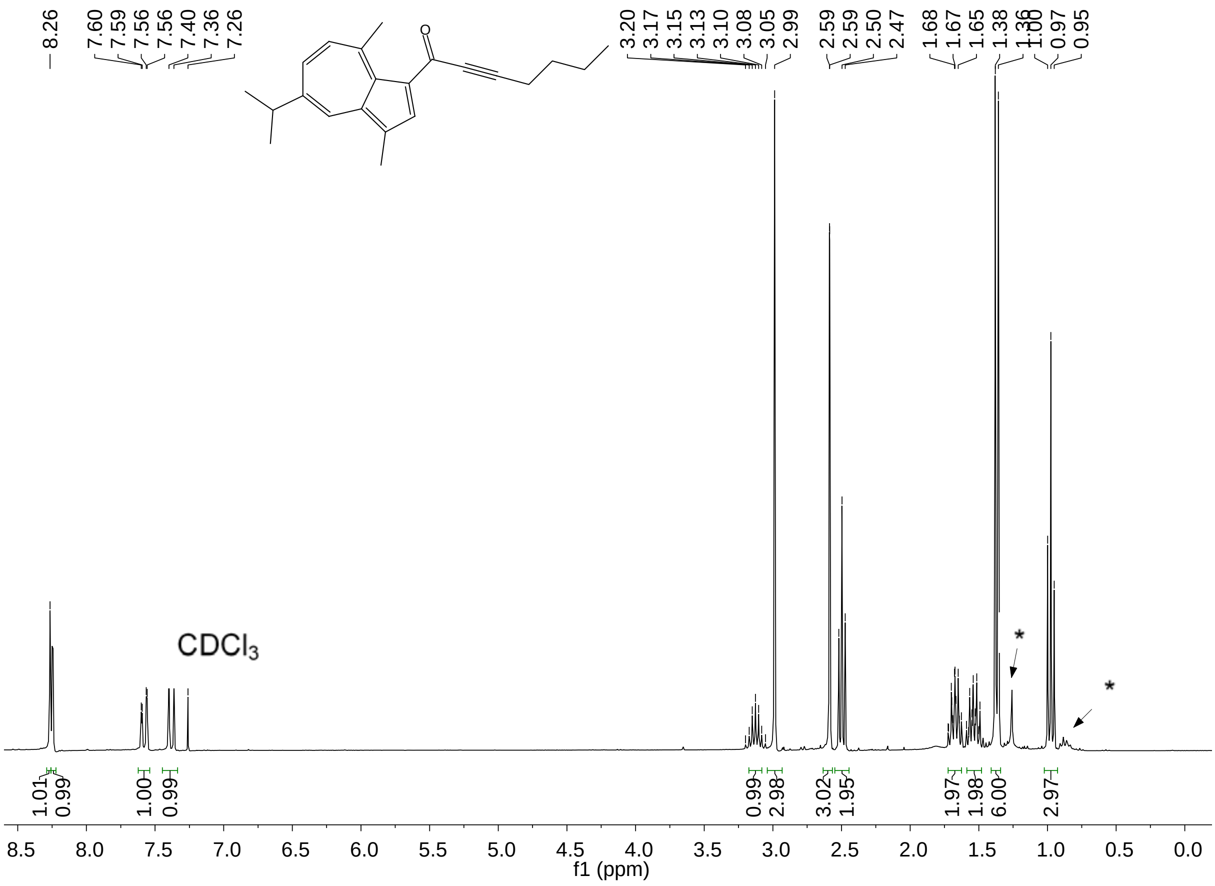

^{1}H NMR of **3k** in $CDCl_3$ at 295 K (δ in ppm). *Impurities from residual solvents.

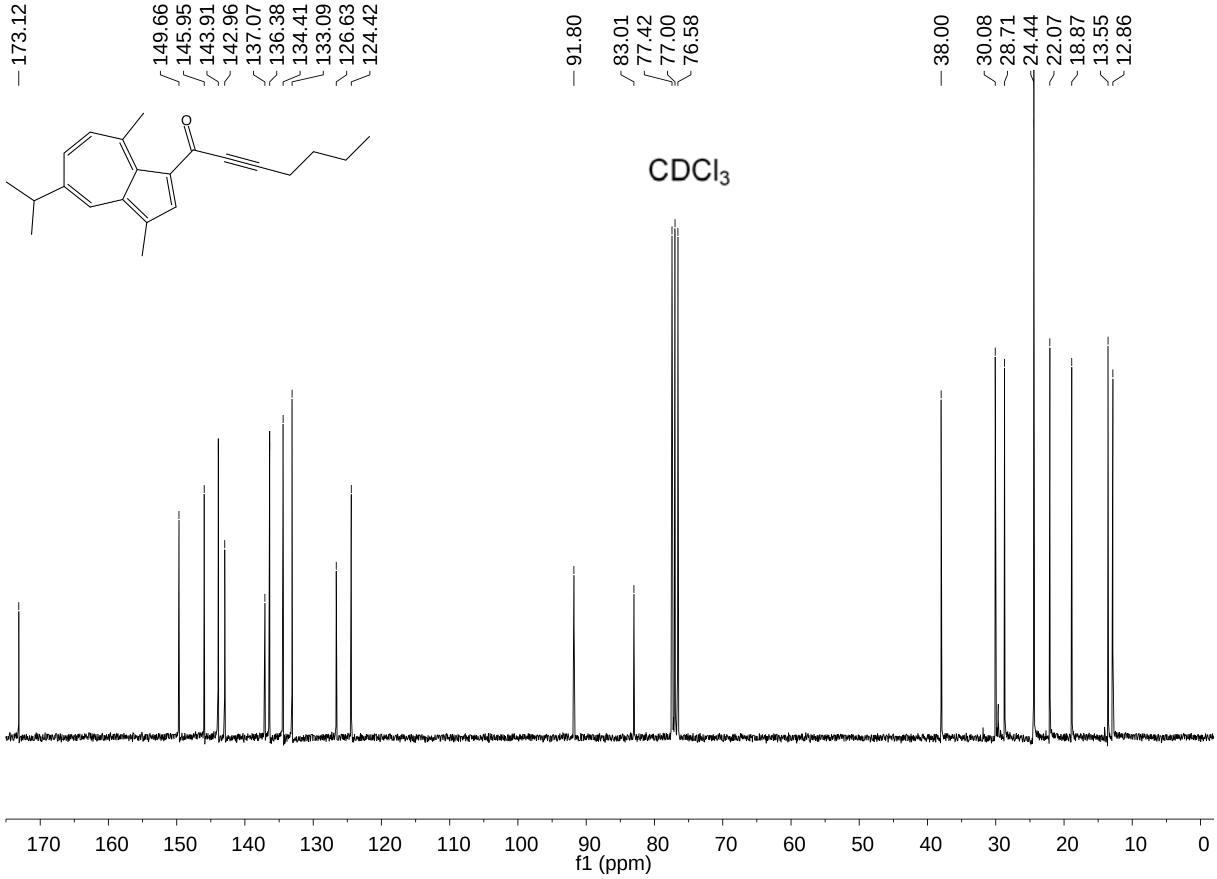

^{13}C NMR of **3k** in $CDCl_3$ at 296 K (δ in ppm).

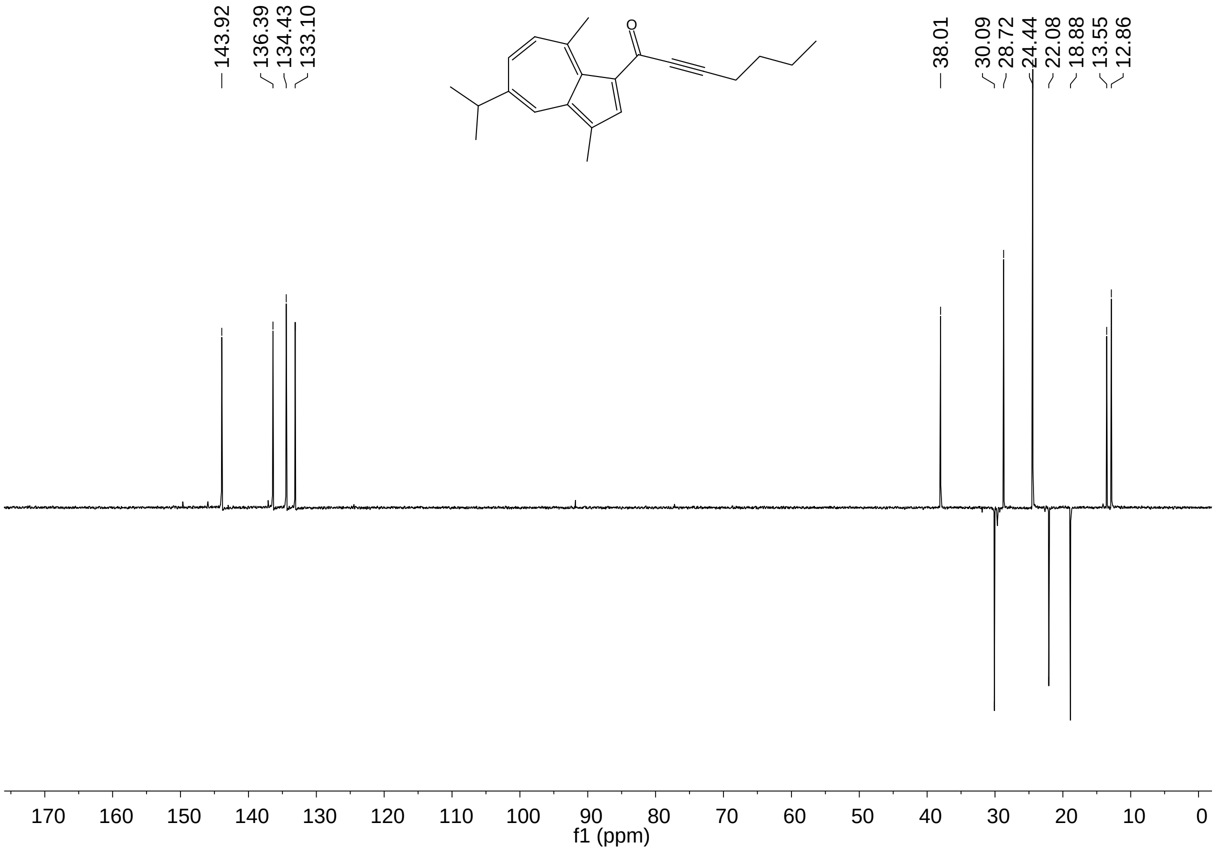

^{13}C DEPT 135-NMR of **3k** in $CDCl_3$ at 295 K (δ in ppm).

5.12 3-Cyclopropyl-1-[3,8-dimethyl-5-(propan-2-yl)azulen-1-yl]prop-2-yn-1-one (3l)

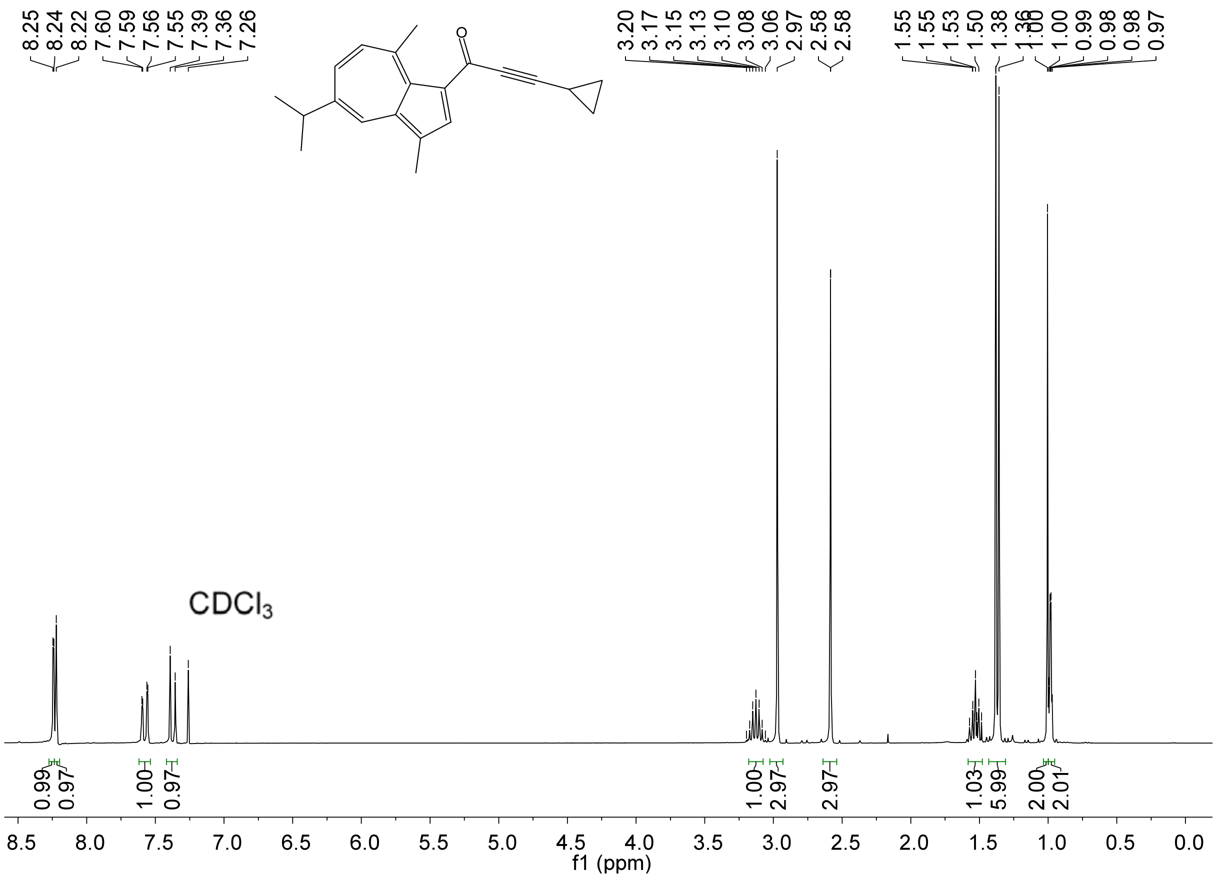

^{1}H NMR of **3l** in $CDCl_3$ at 298 K (δ in ppm).

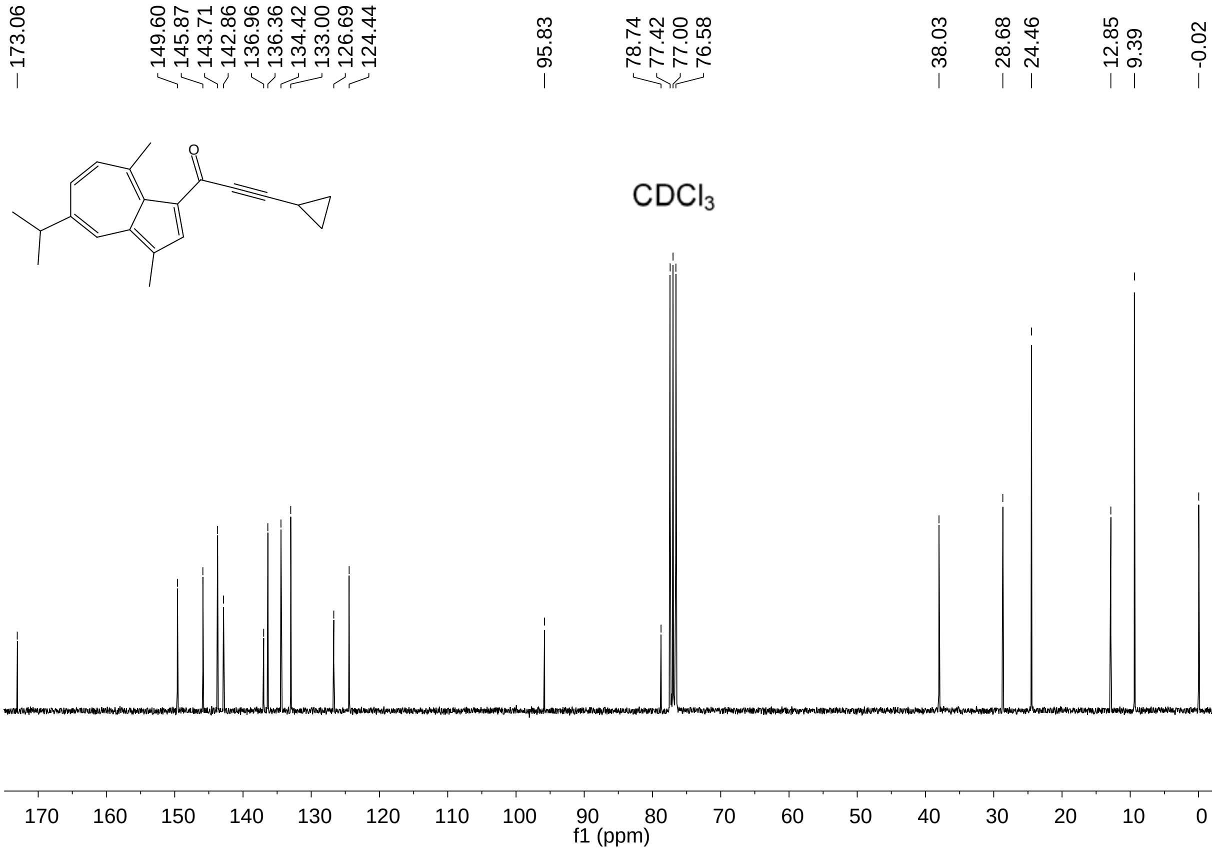

^{13}C NMR of **3l** in $CDCl_3$ at 298 K (δ in ppm).

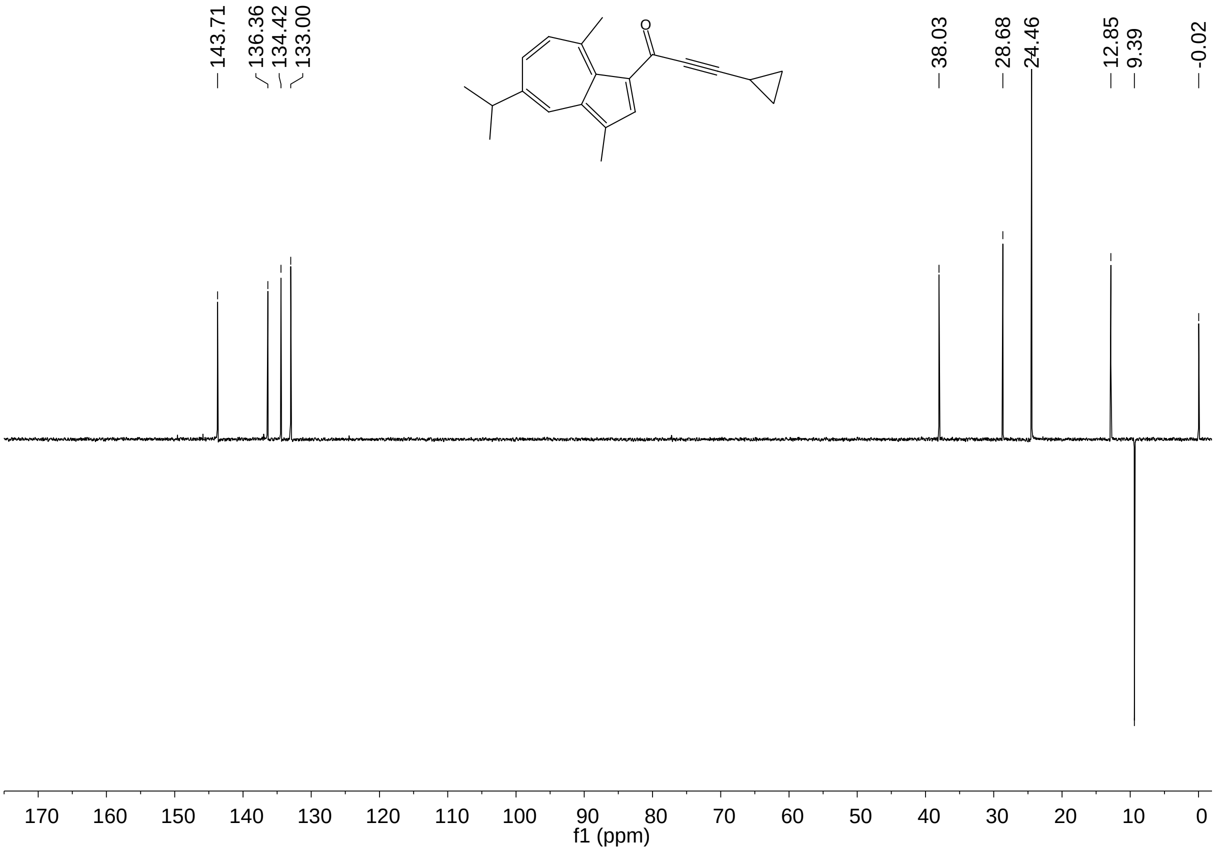

^{13}C DEPT 135-NMR of **3l** at in $CDCl_3$ 298 K (δ in ppm).

5.13 1-[3,8-Dimethyl-5-(propan-2-yl)azulen-1-yl]-4,4-diethoxybut-2-yn-1-one (3m)

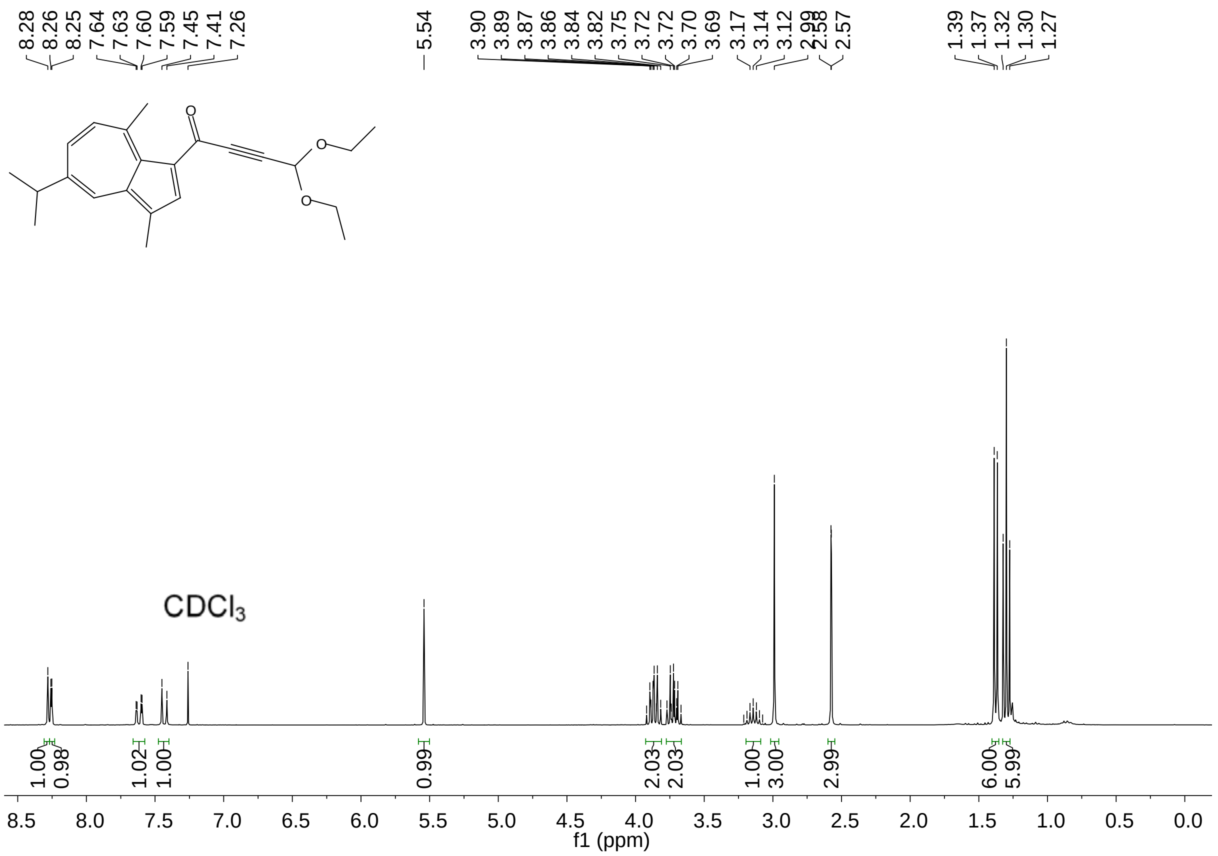

^{1}H NMR of **3m** in $CDCl_3$ at 298 K (δ in ppm).

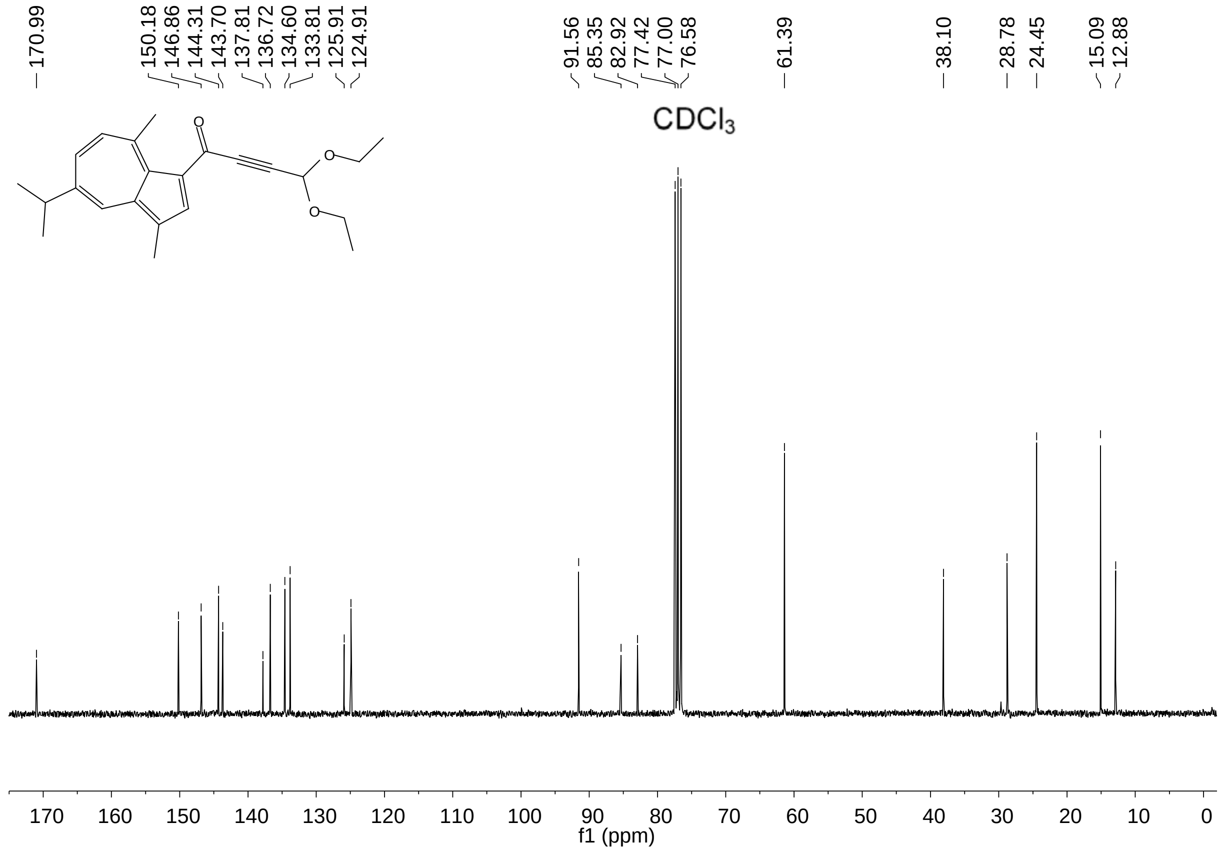

^{13}C NMR of **3m** in $CDCl_3$ at 298 K (δ in ppm).

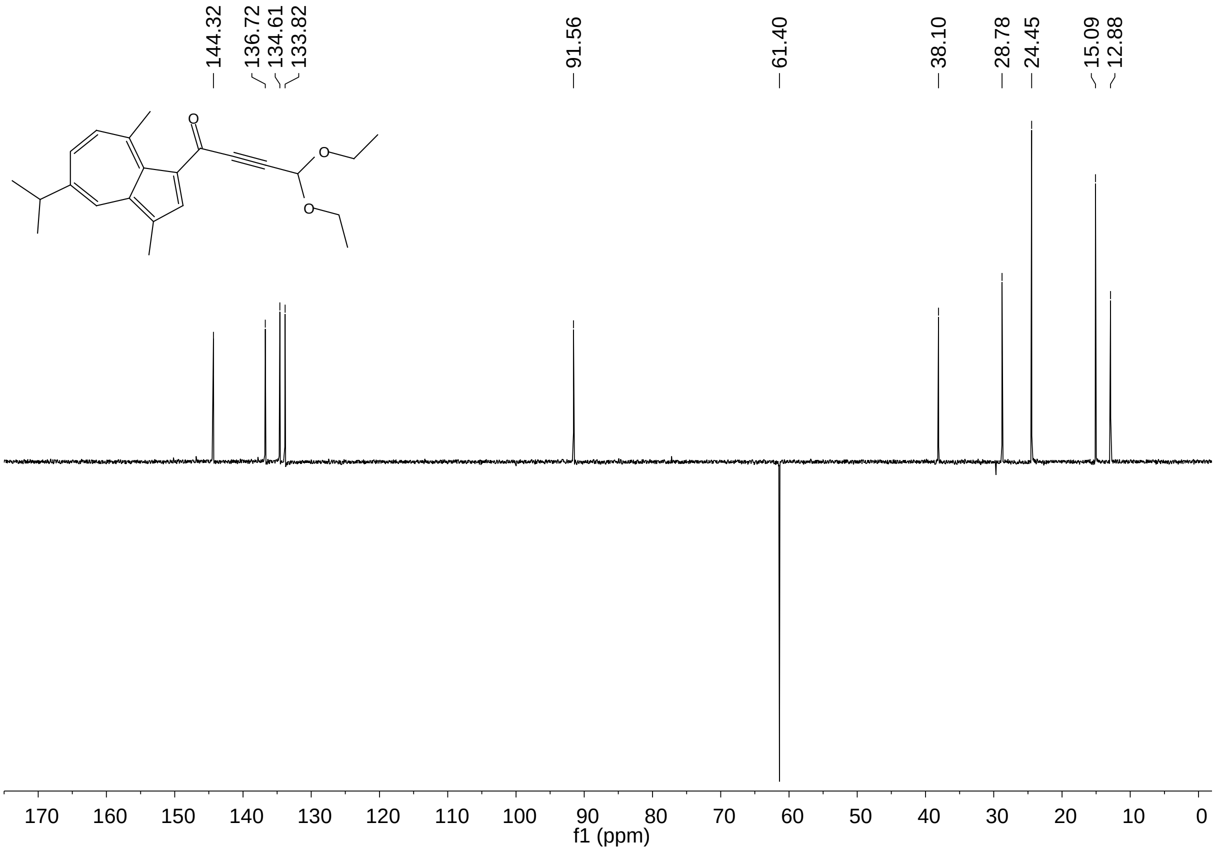

^{13}C DEPT 135-NMR of **3m** in $CDCl_3$ at 298 K (δ in ppm).

5.14 1-[3,8-Dimethyl-5-(propan-2-yl)azulen-1-yl]-3-[tris(propan-2-yl)silyl]prop-2-yn-1-one (3n)

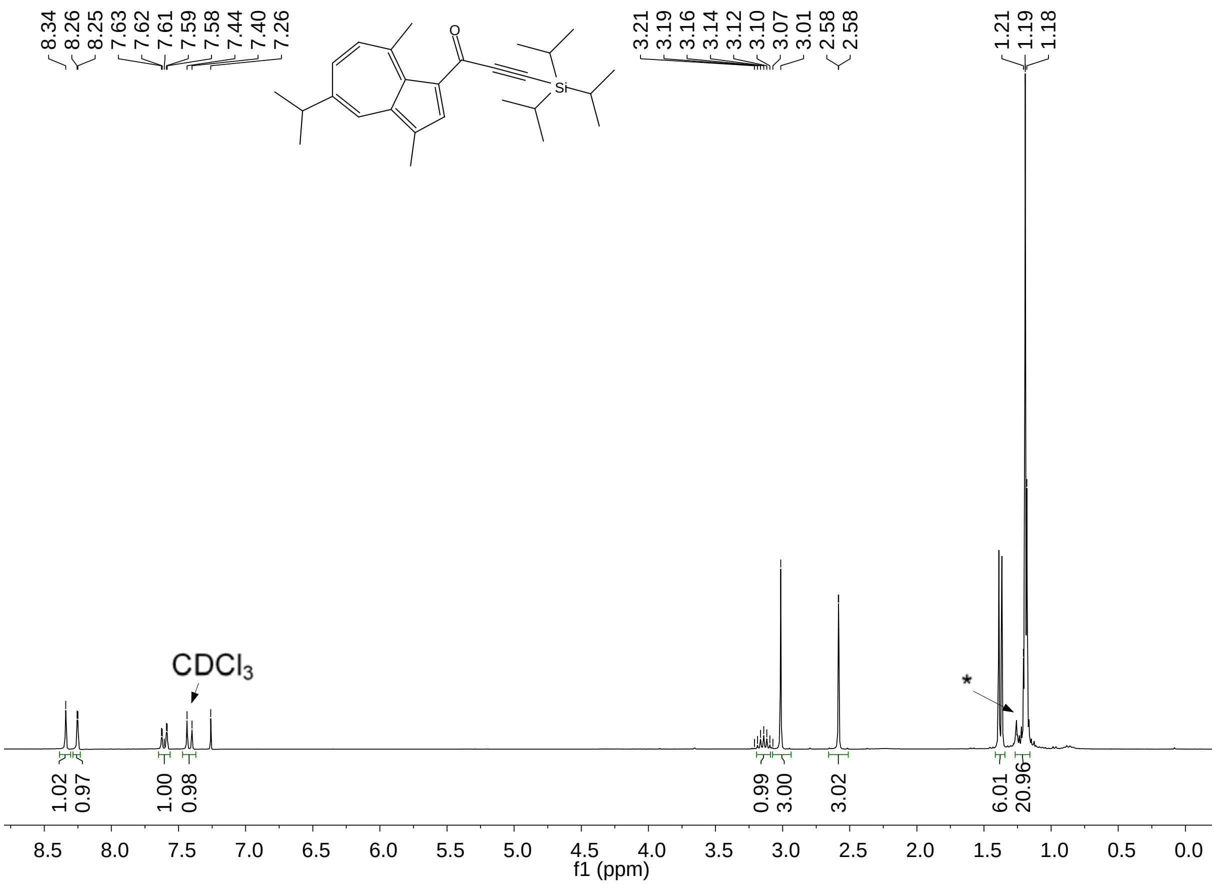

^{1}H NMR of **3n** in $CDCl_3$ at 295 K (δ in ppm). *Impurities from residual solvents.

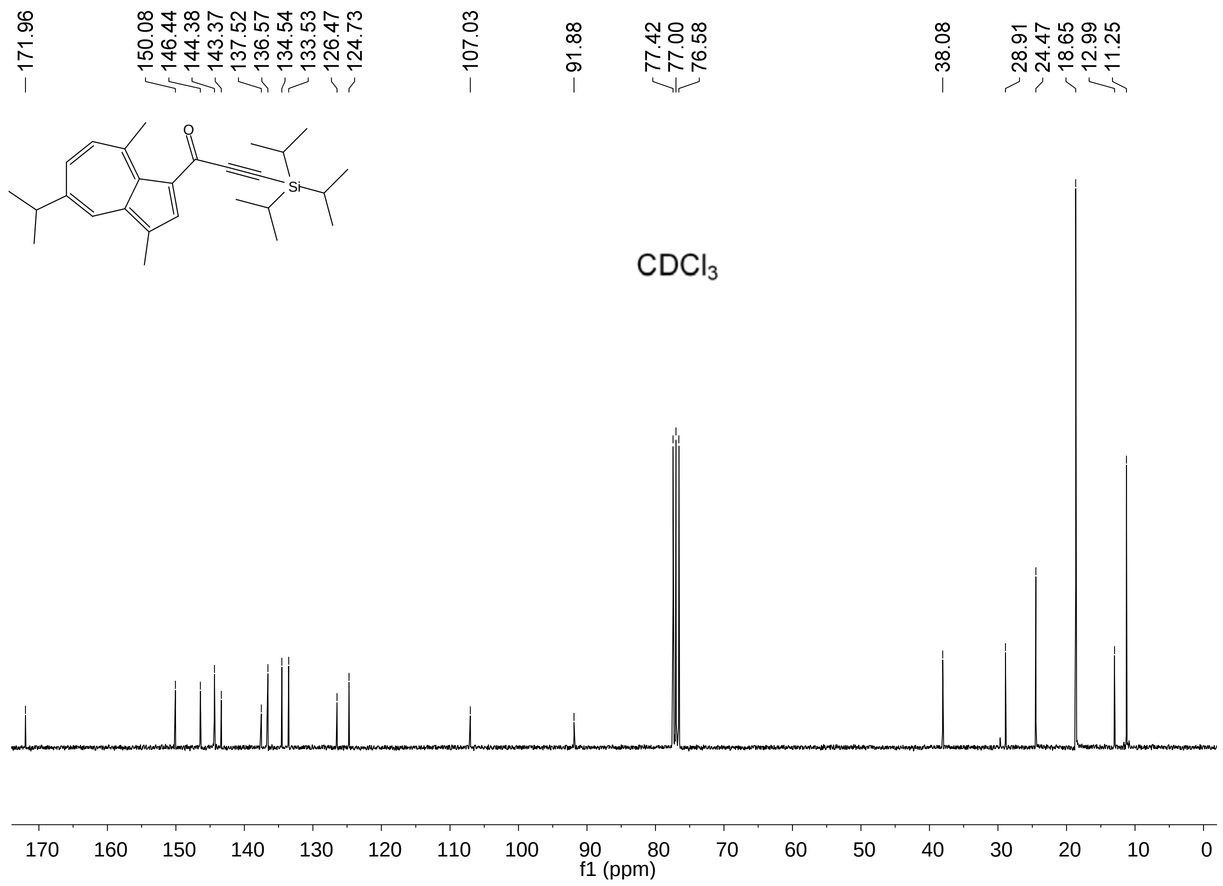

^{13}C NMR of **3n** in $CDCl_3$ at 296 K (δ in ppm).

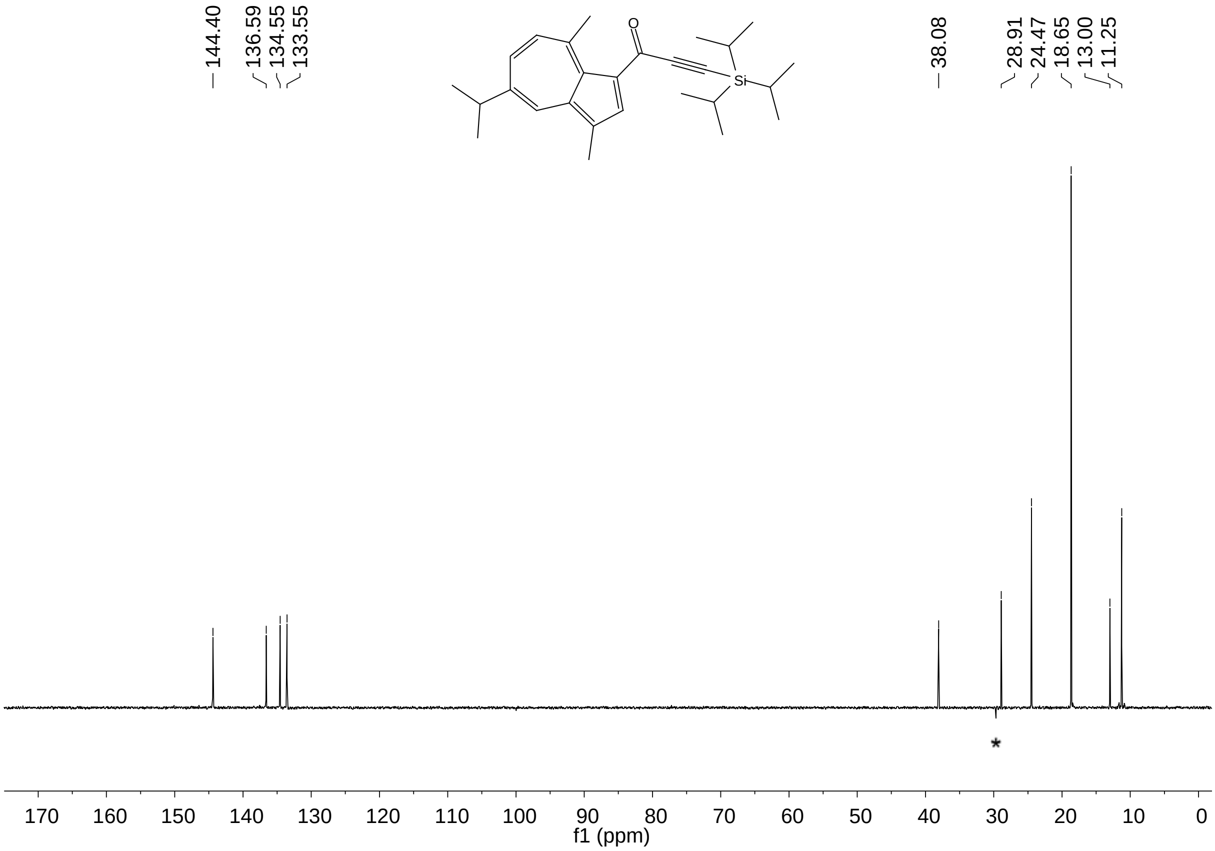

^{13}C DEPT 135-NMR of **3n** in $CDCl_3$ at 296 K (δ in ppm). *Impurities from residual solvents.

6 ^{1}H and ^{13}C NMR Spectra of Pyrimidines 5

6.1 4-(Azulen-1-yl)-2,6-diphenylpyrimidine (5a)

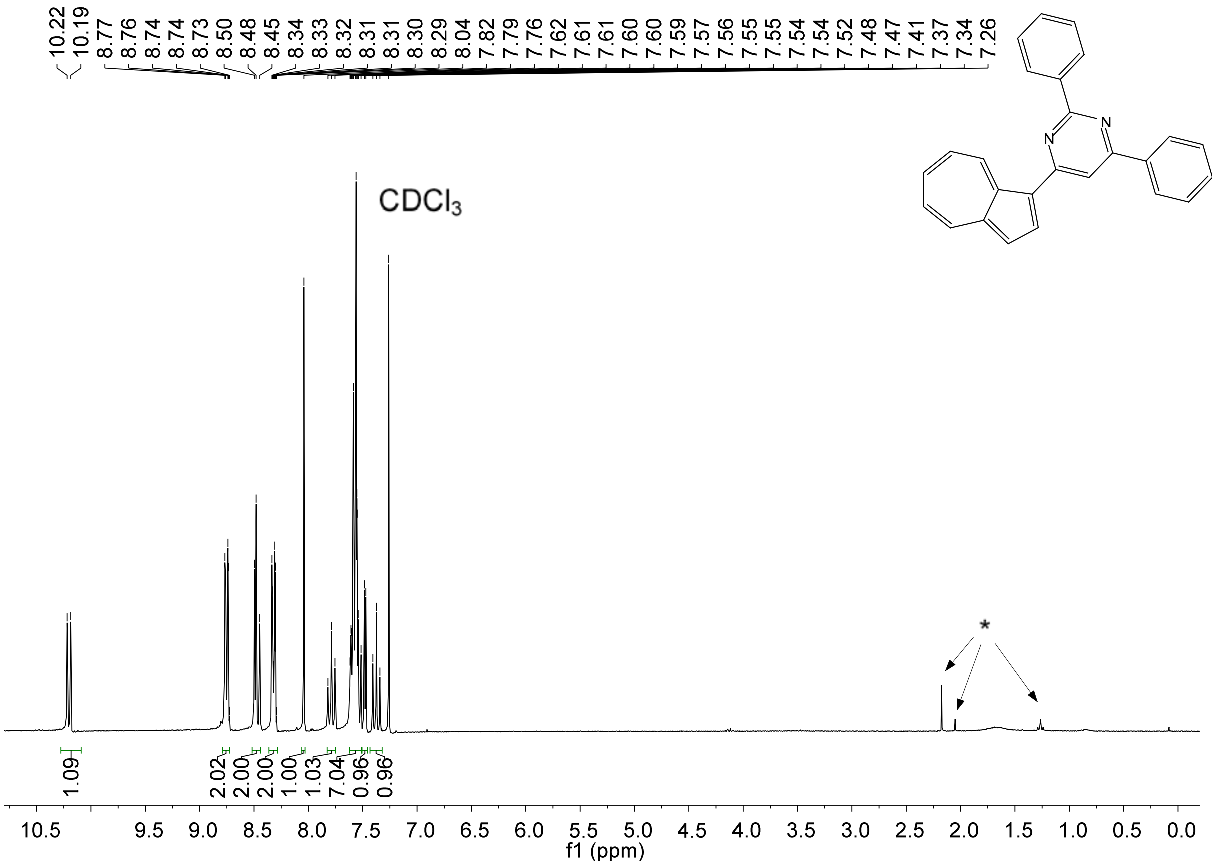

^{1}H NMR of **5a** in $CDCl_3$ at 298 K (δ in ppm). *Impurities from residual solvents.

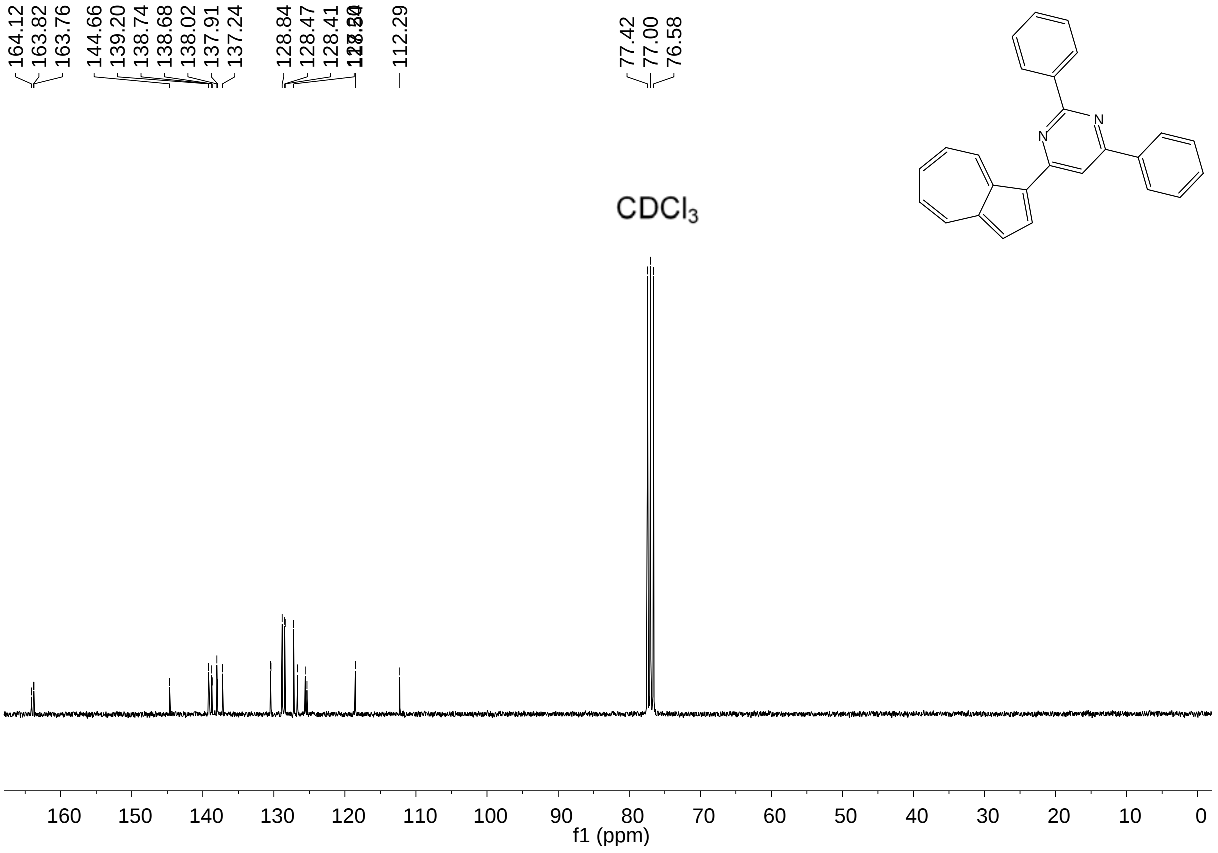

^{13}C NMR of **5a** in $CDCl_3$ at 298 K (δ in ppm).

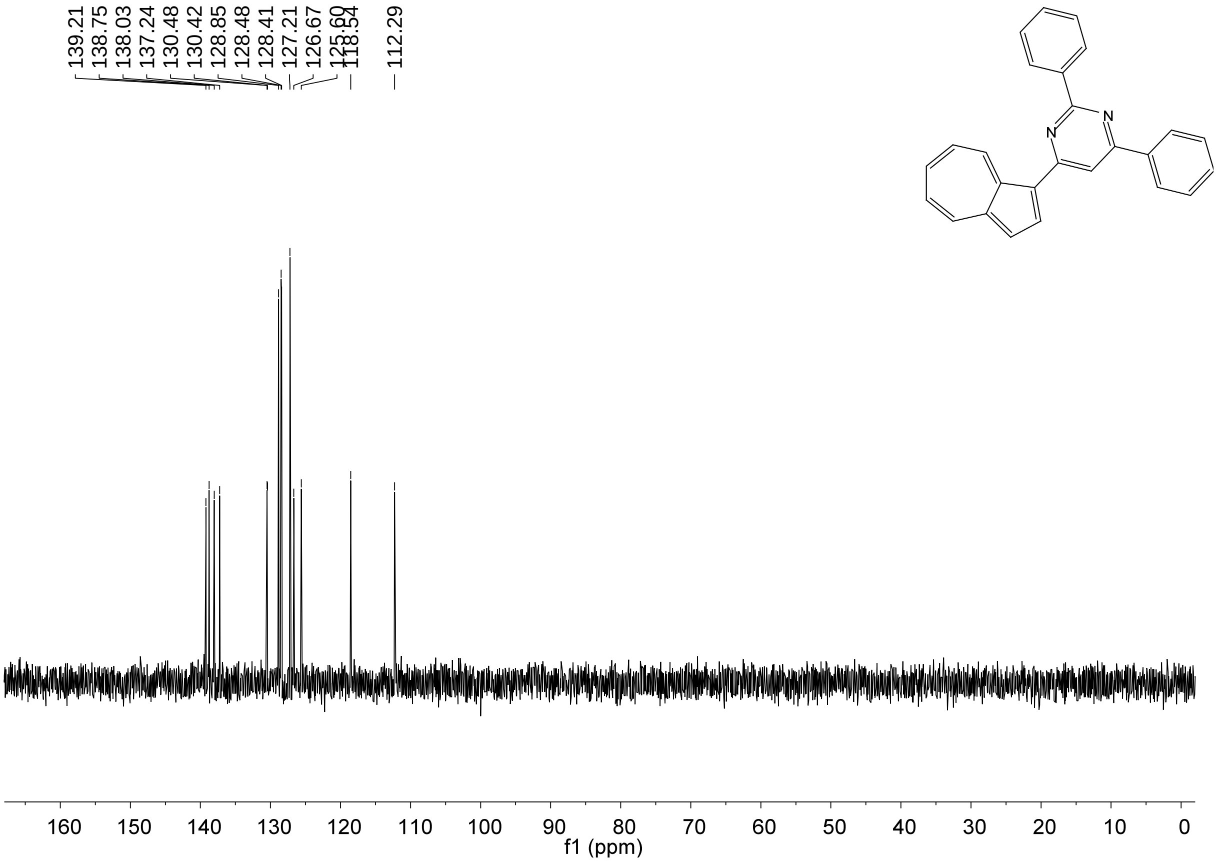

^{13}C DEPT 135-NMR of **5a** in $CDCl_3$ at 298 K (δ in ppm).

6.2 4-(Azulen-1-yl)-6-phenyl-2-(thiophen-2-yl)pyrimidine (5b)

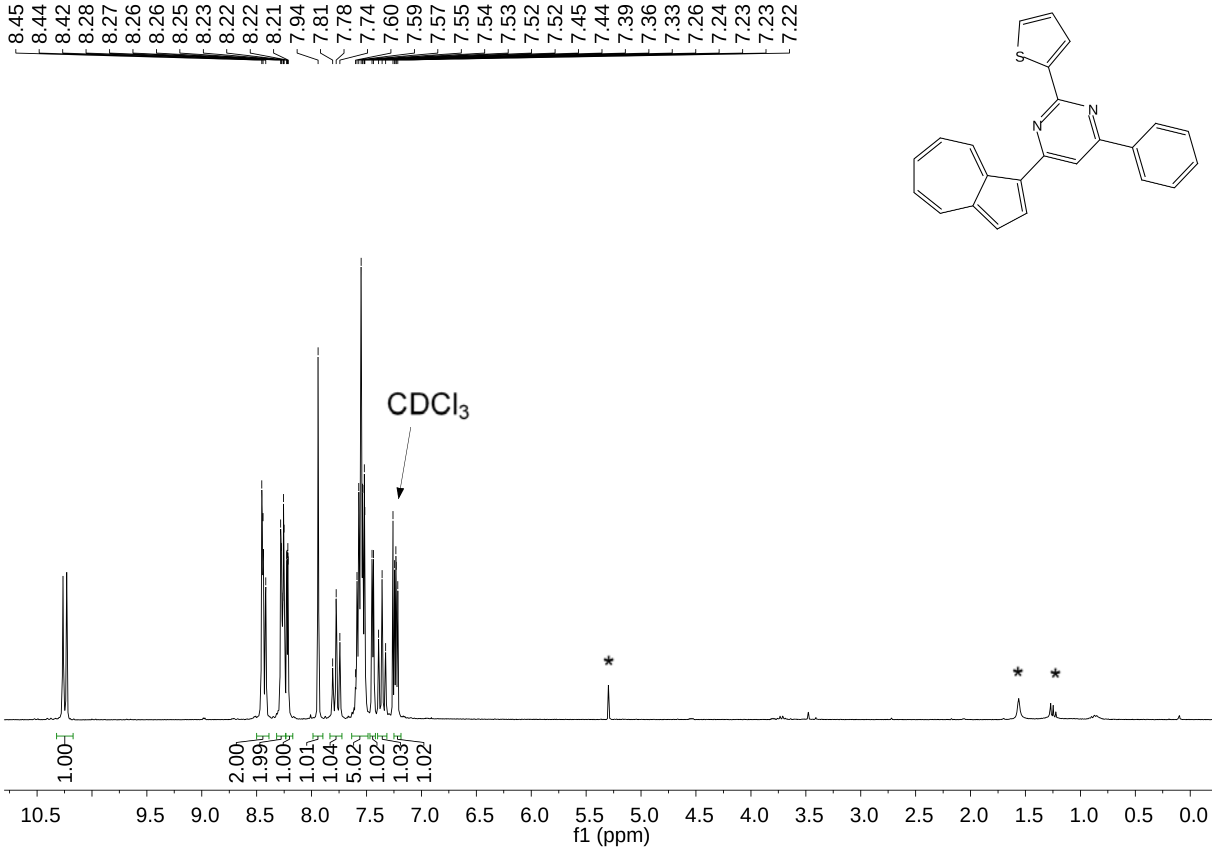

^{1}H NMR of **5b** in $CDCl_3$ at 298 K (δ in ppm). *Impurities from residual solvents.

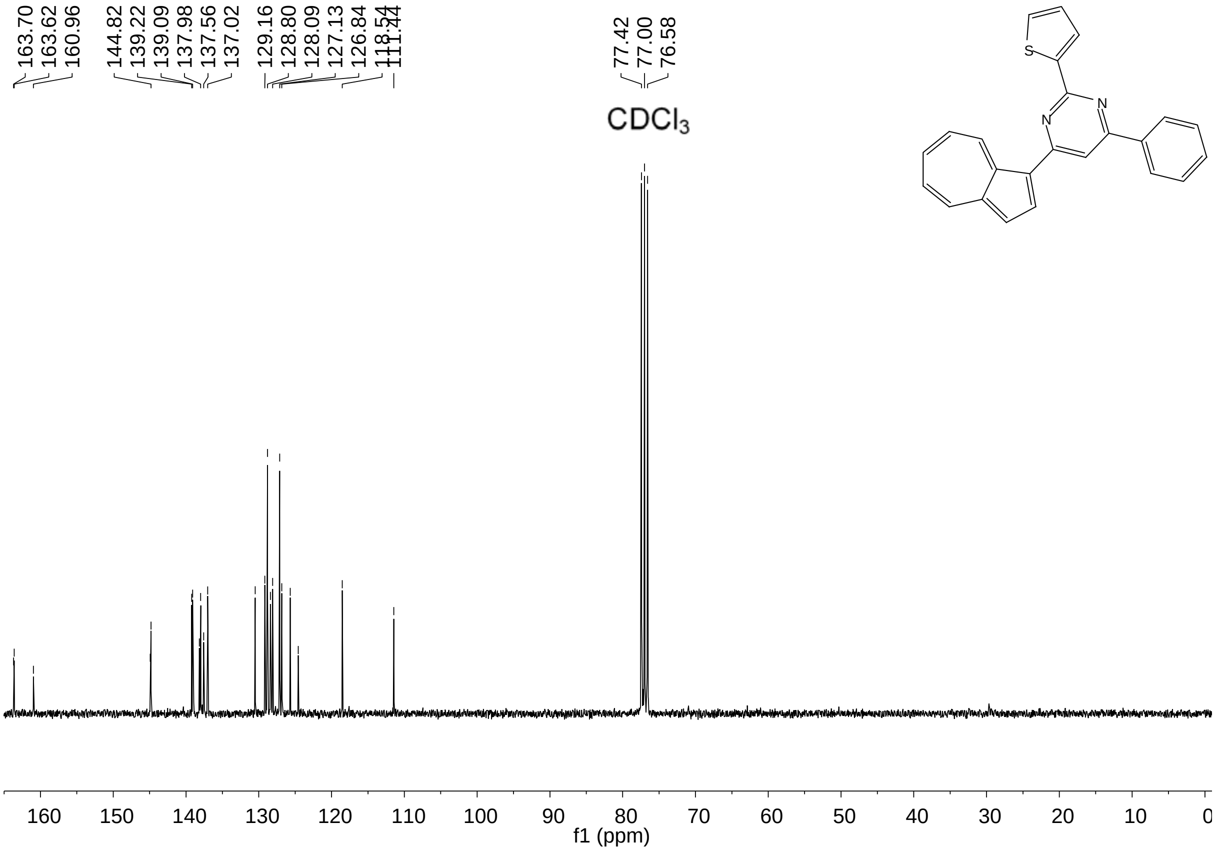

^{13}C NMR of **5b** in $CDCl_3$ at 298 K (δ in ppm).

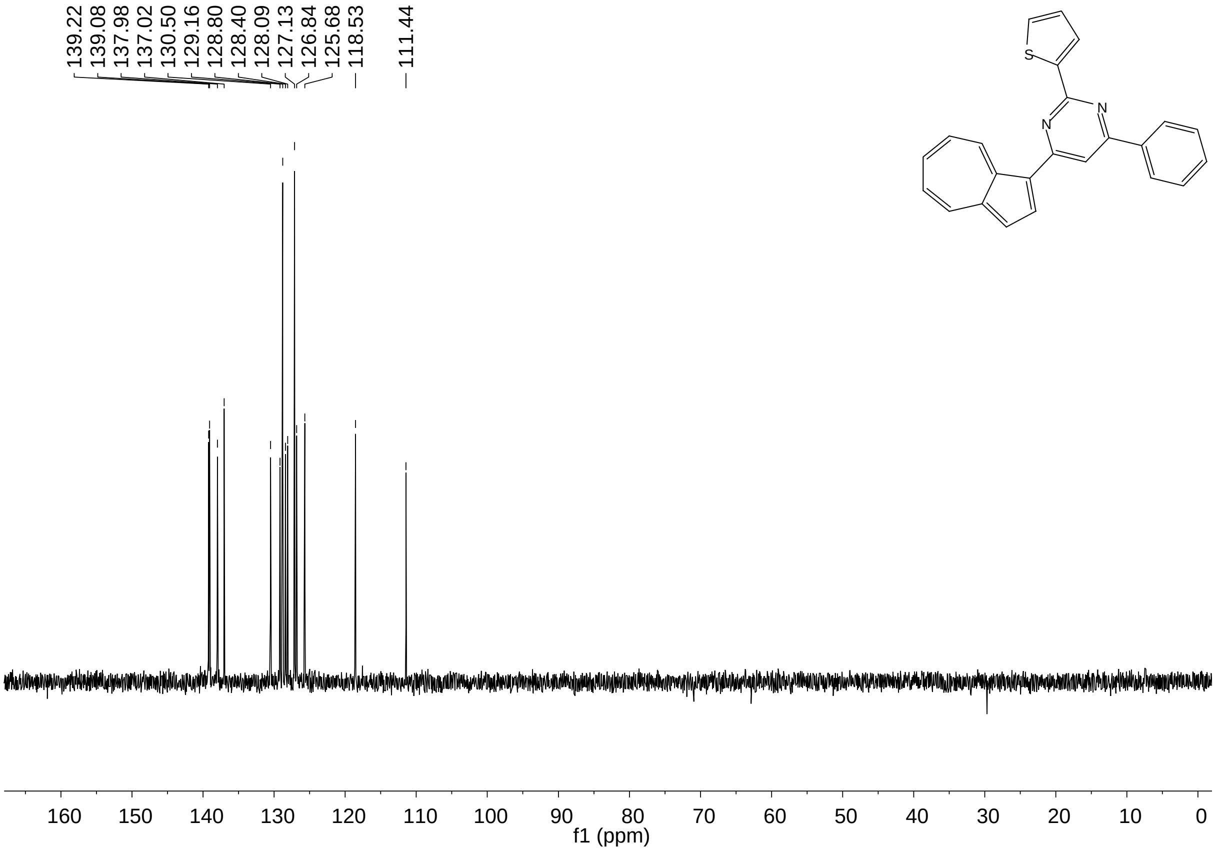

^{13}C DEPT 135-NMR of **5b** in $CDCl_3$ at 298 K (δ in ppm).

6.3 4-(Azulen-1-yl)-2-methyl-6-phenylpyrimidine (5c)

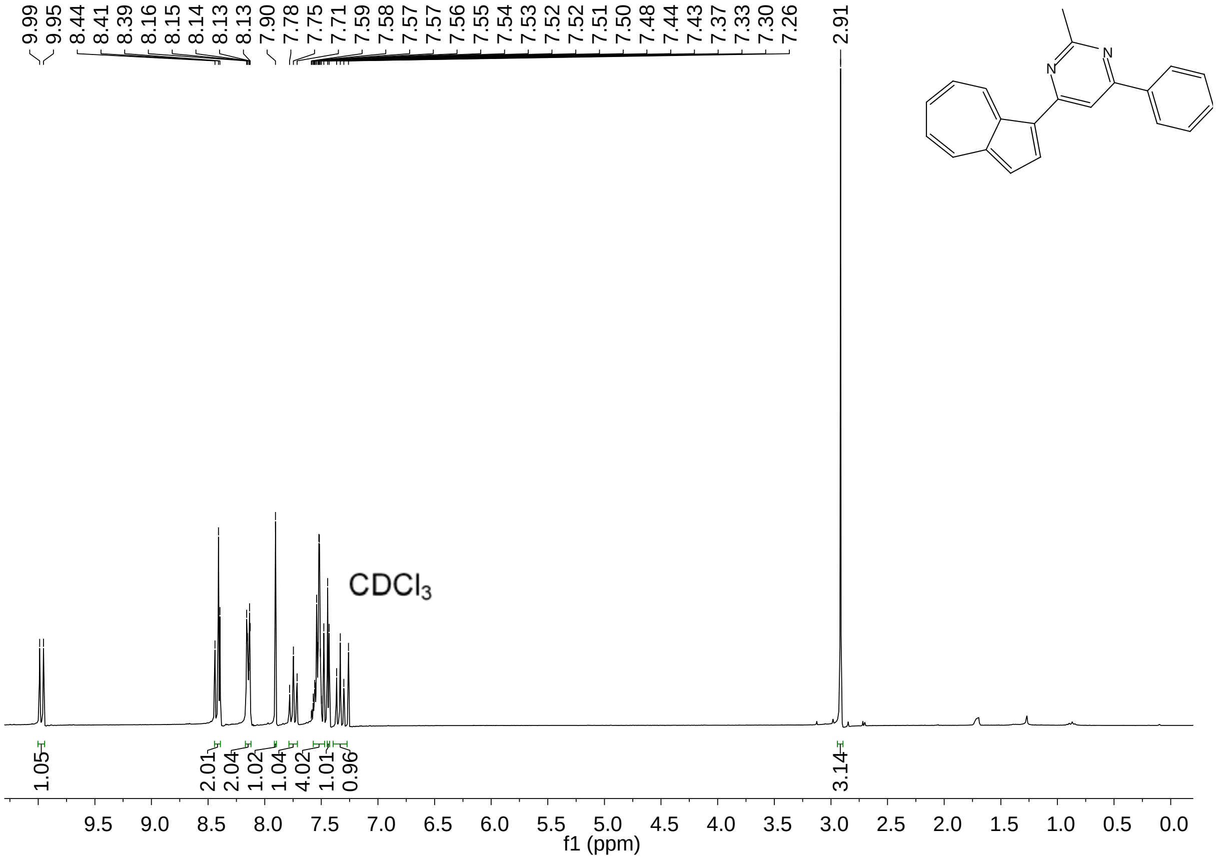

^{1}H NMR of **5c** in $CDCl_3$ at 298 K (δ in ppm).

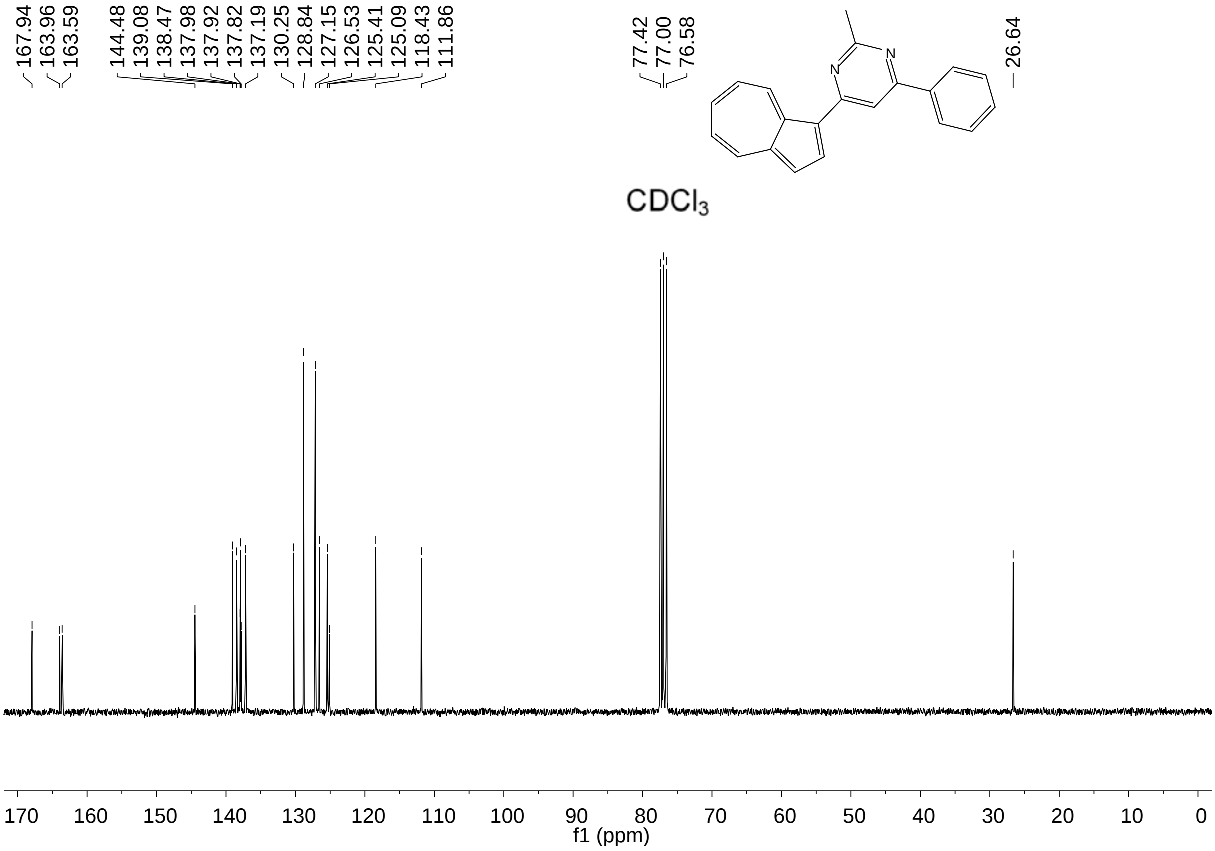

^{13}C NMR of **5c** in $CDCl_3$ at 298 K (δ in ppm).

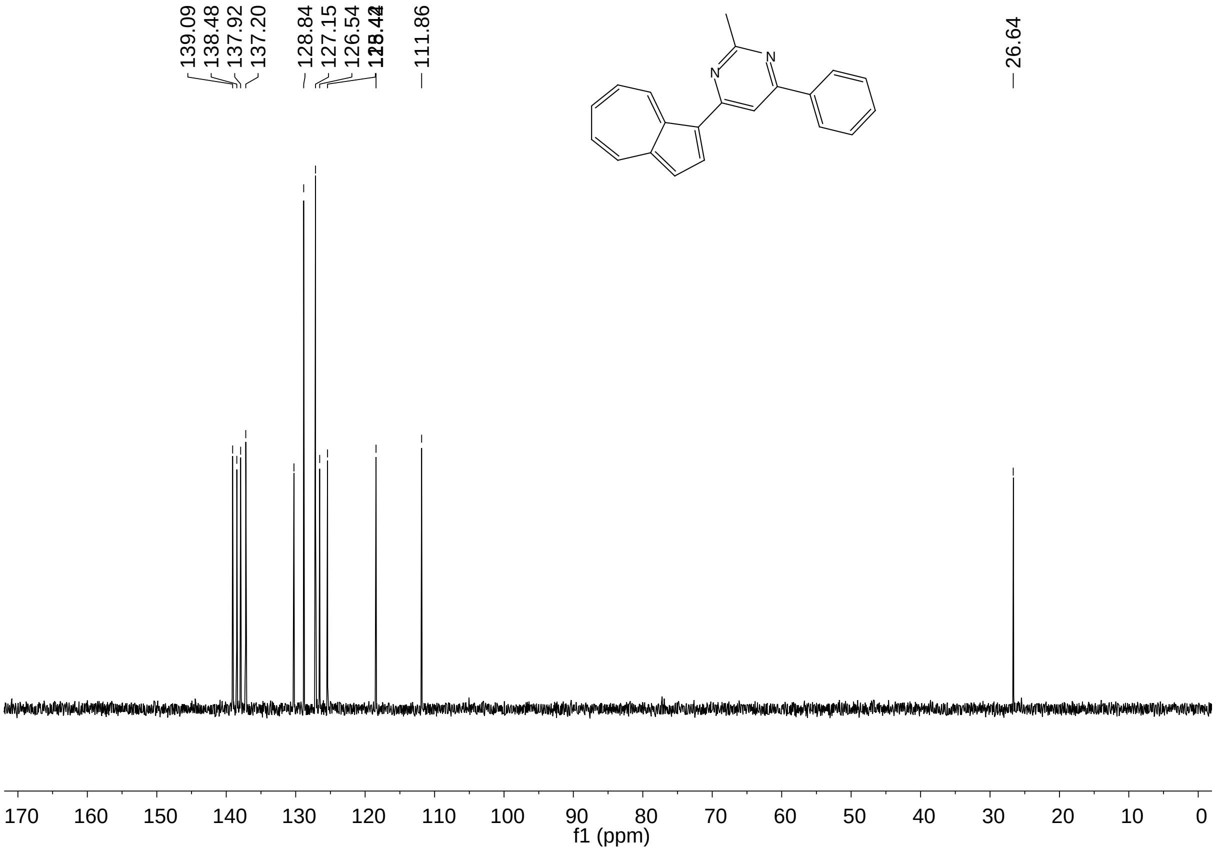

^{13}C DEPT 135-NMR of **5c** in $CDCl_3$ at 298 K (δ in ppm).

6.4 4-(Azulen-1-yl)-6-butyl-2-phenylpyrimidine (5d)

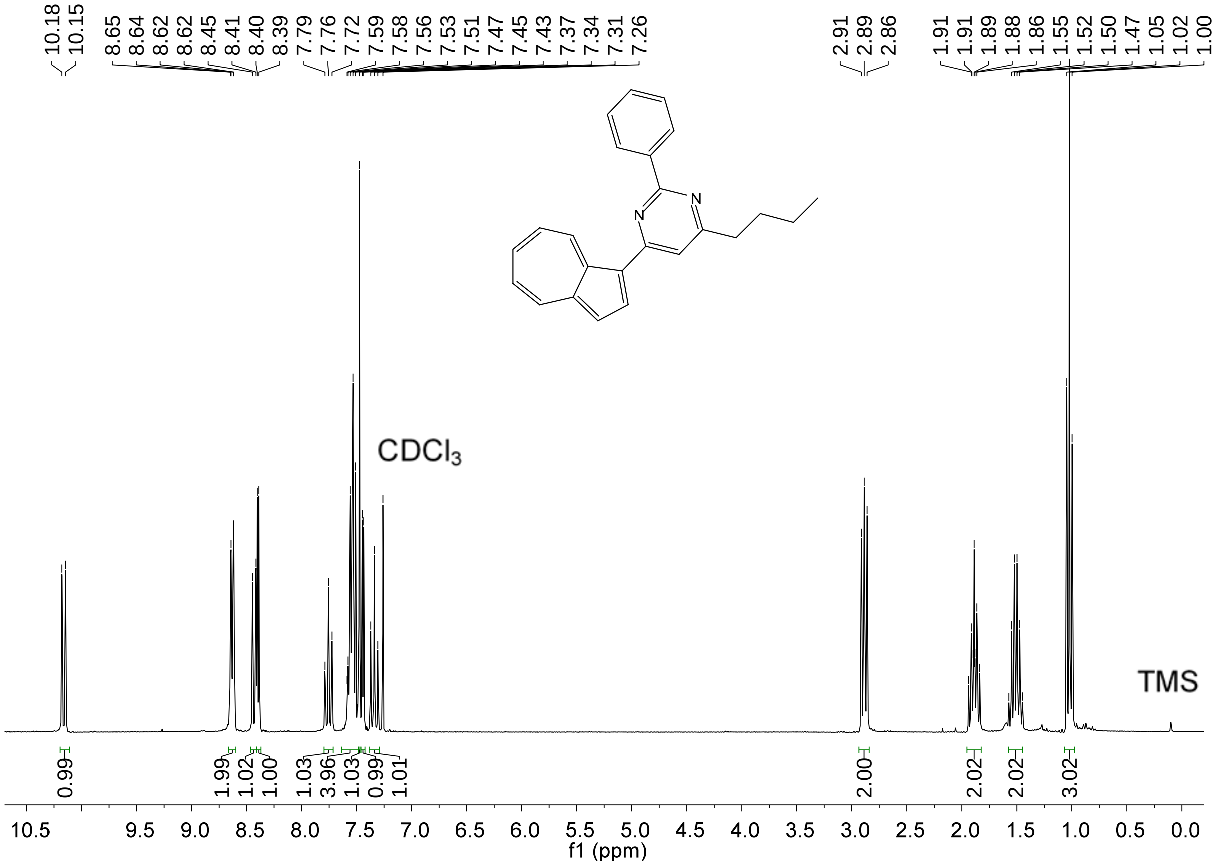

^{1}H NMR of **5d** in $CDCl_3$ at 298 K (δ in ppm).

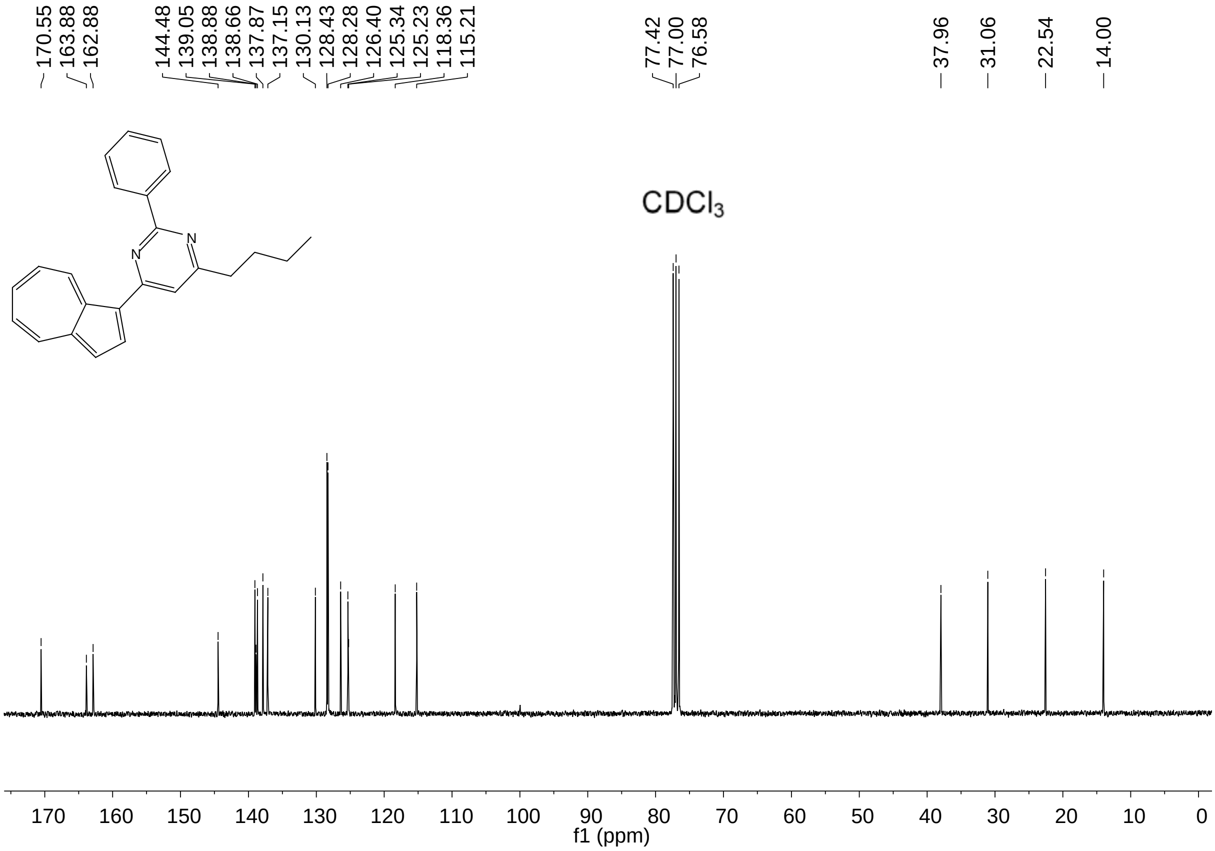

^{13}C NMR of **5d** in $CDCl_3$ at 298 K (δ in ppm).

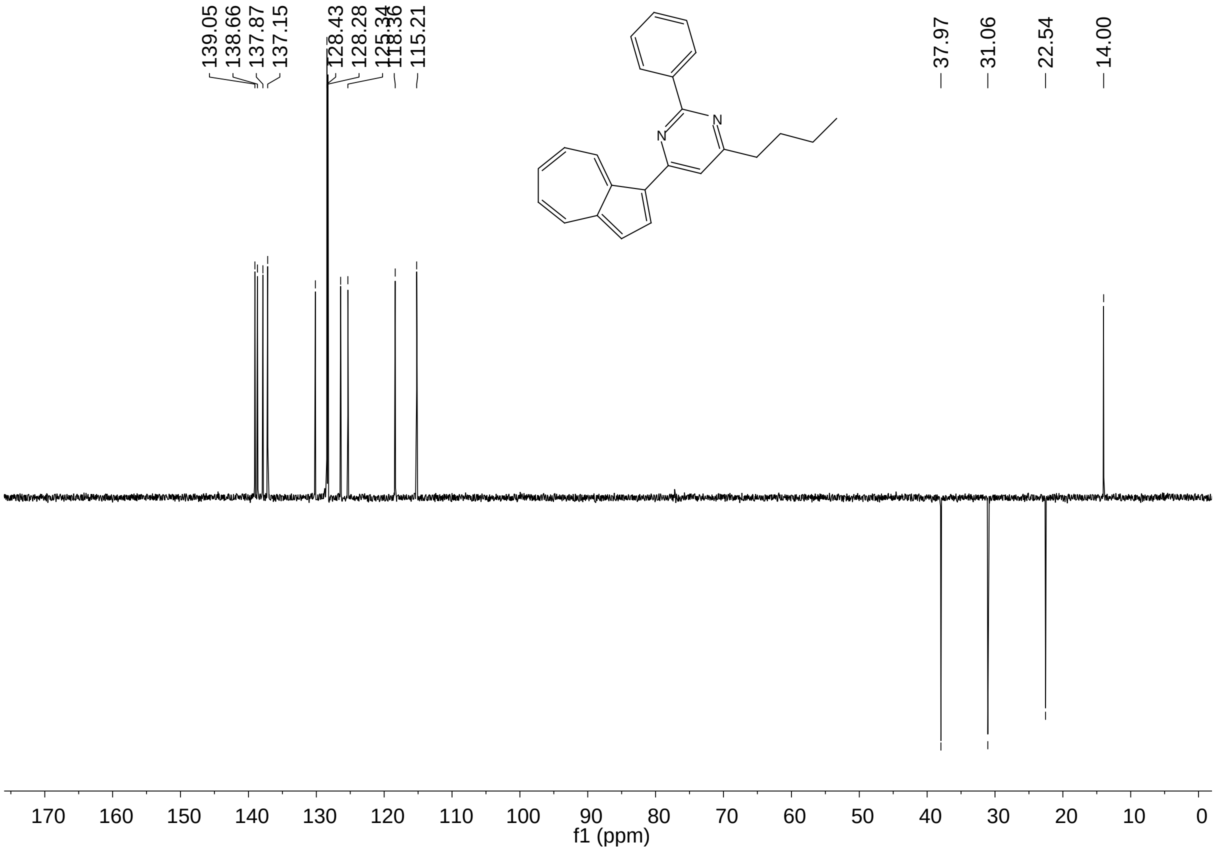

^{13}C DEPT 135-NMR of **5d** in $CDCl_3$ at 298 K (δ in ppm).

6.5 4-[3,8-Dimethyl-5-(propan-2-yl)azulen-1-yl]-2,6-diphenylpyrimidine (5e)

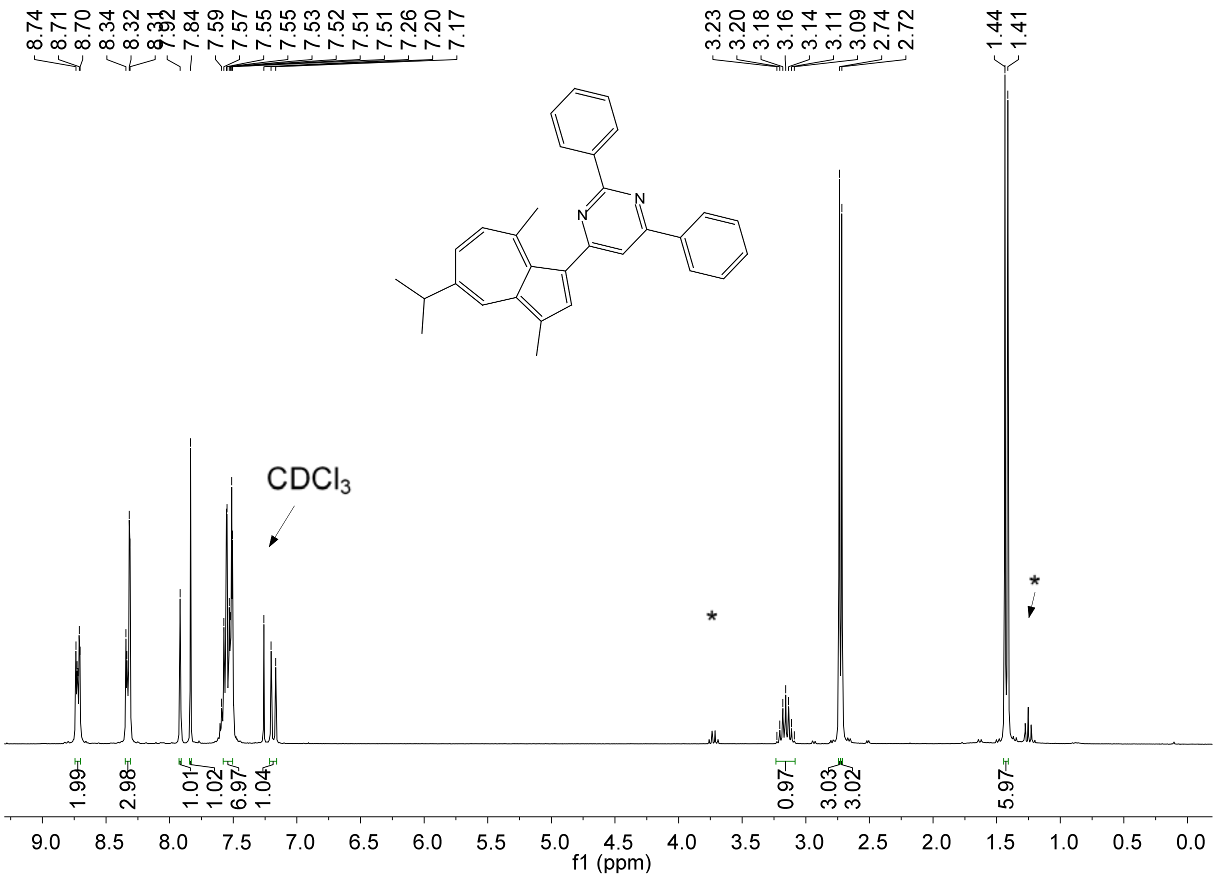

^{1}H NMR of **5e** in $CDCl_3$ at 298 K (δ in ppm). *Impurities from residual ethanol.

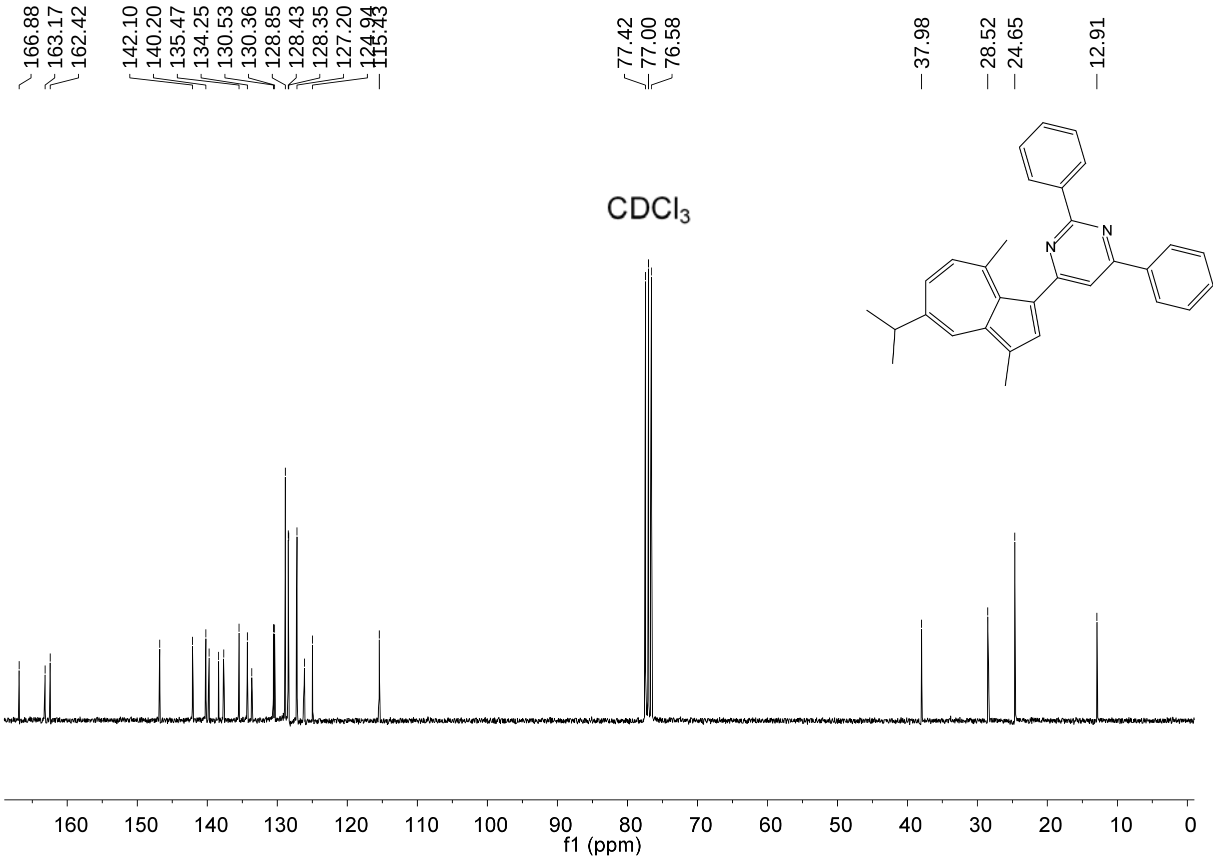

^{13}C NMR of **5e** in $CDCl_3$ at 298 K (δ in ppm).

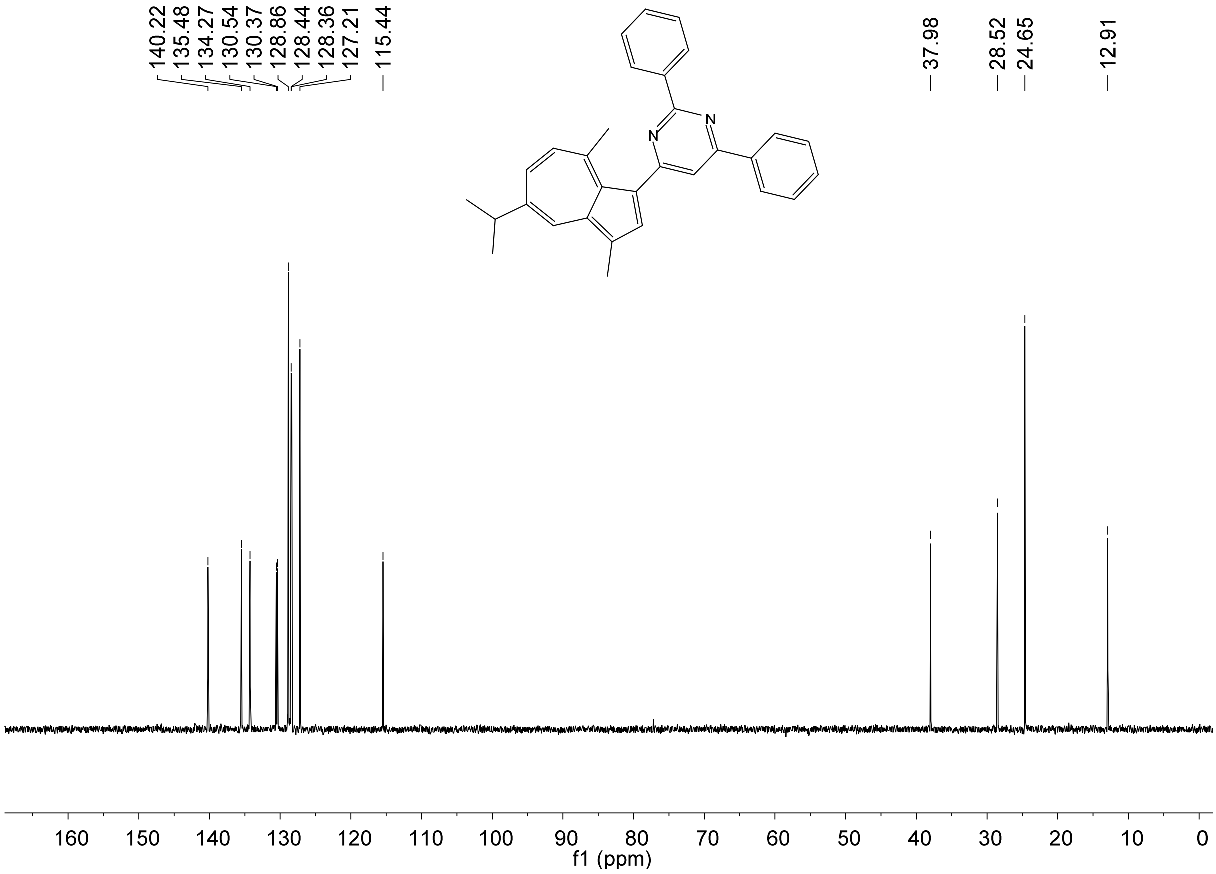

^{13}C DEPT 135-NMR of **5e** in $CDCl_3$ at 298 K (δ in ppm).

6.6 4-Butyl-6-[3,8-dimethyl-5-(propan-2-yl)azulen-1-yl]-2-phenylpyrimidine (5f)

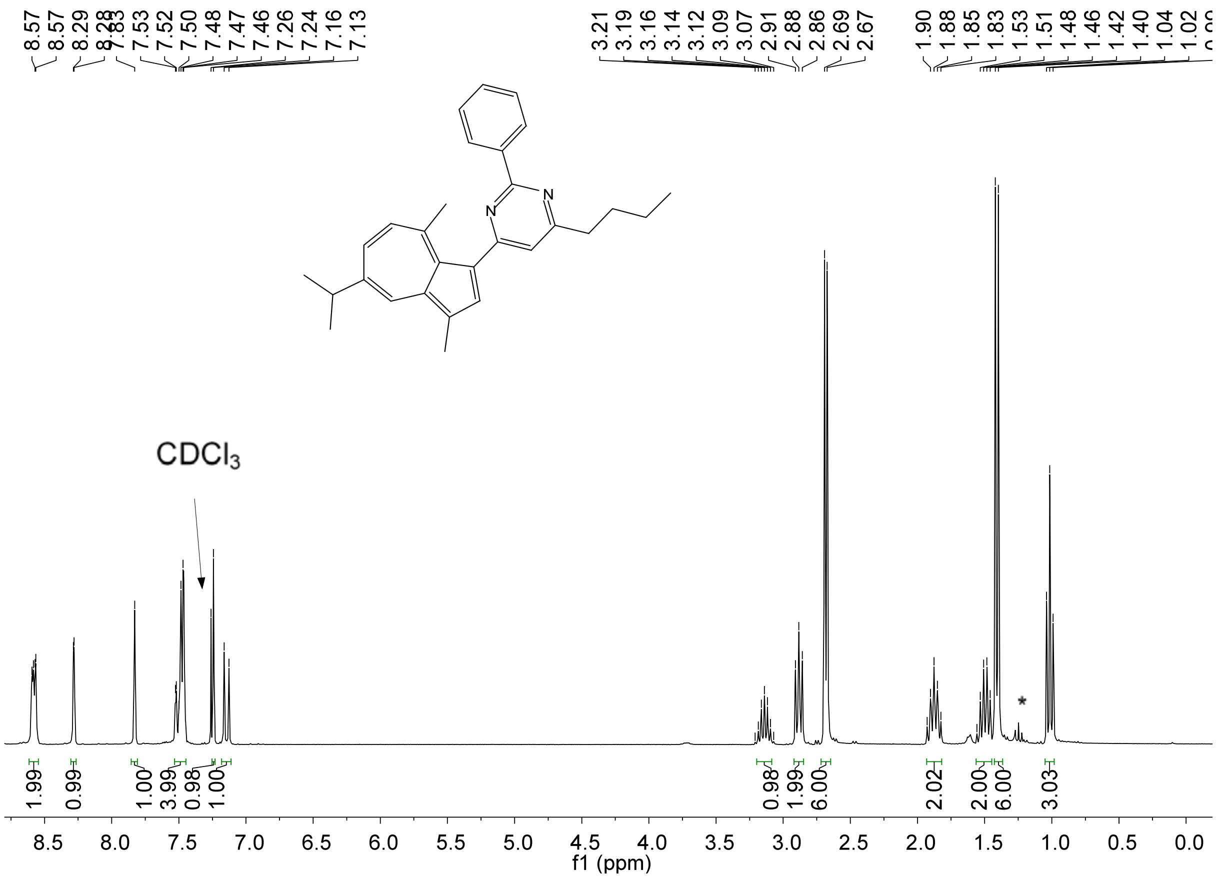

^{1}H NMR of **5f** in $CDCl_3$ at 298 K (δ in ppm). *Impurities from residual ethanol.

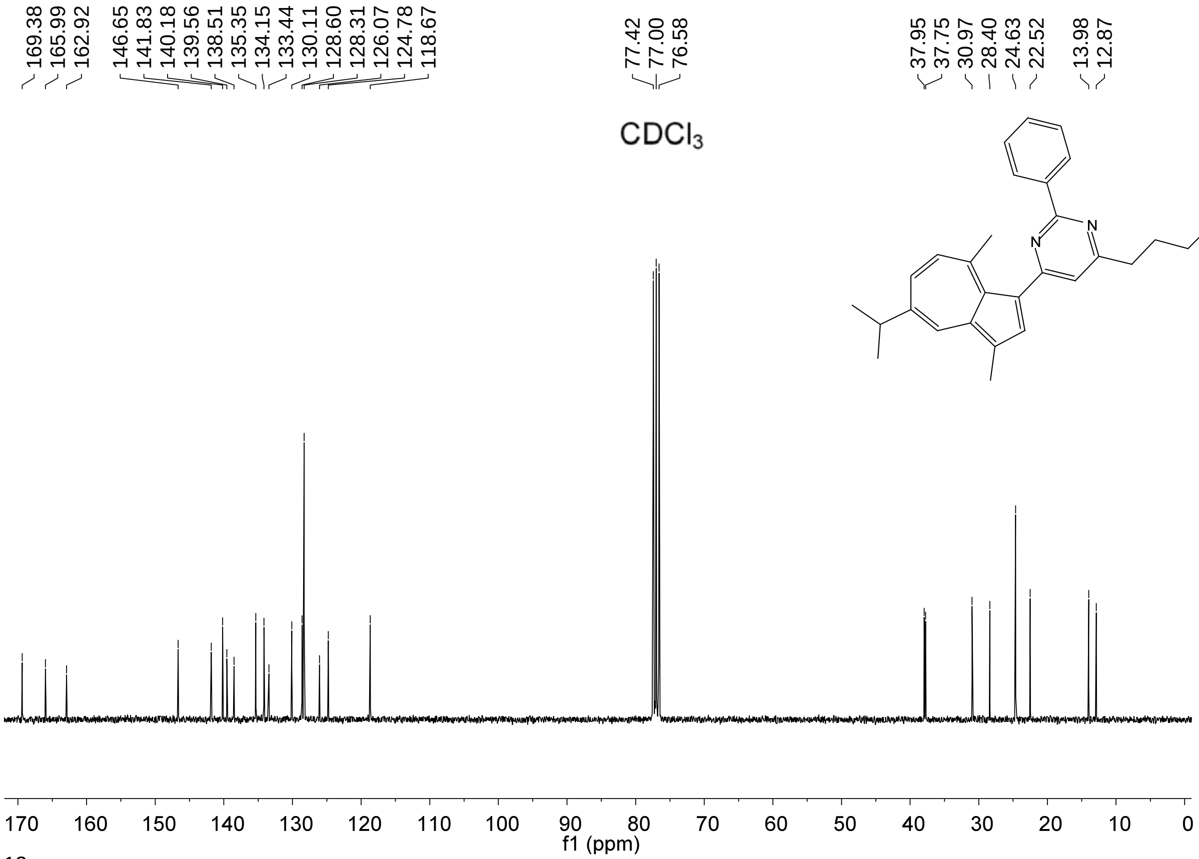

^{13}C NMR of **5f** in $CDCl_3$ at 298 K (δ in ppm).

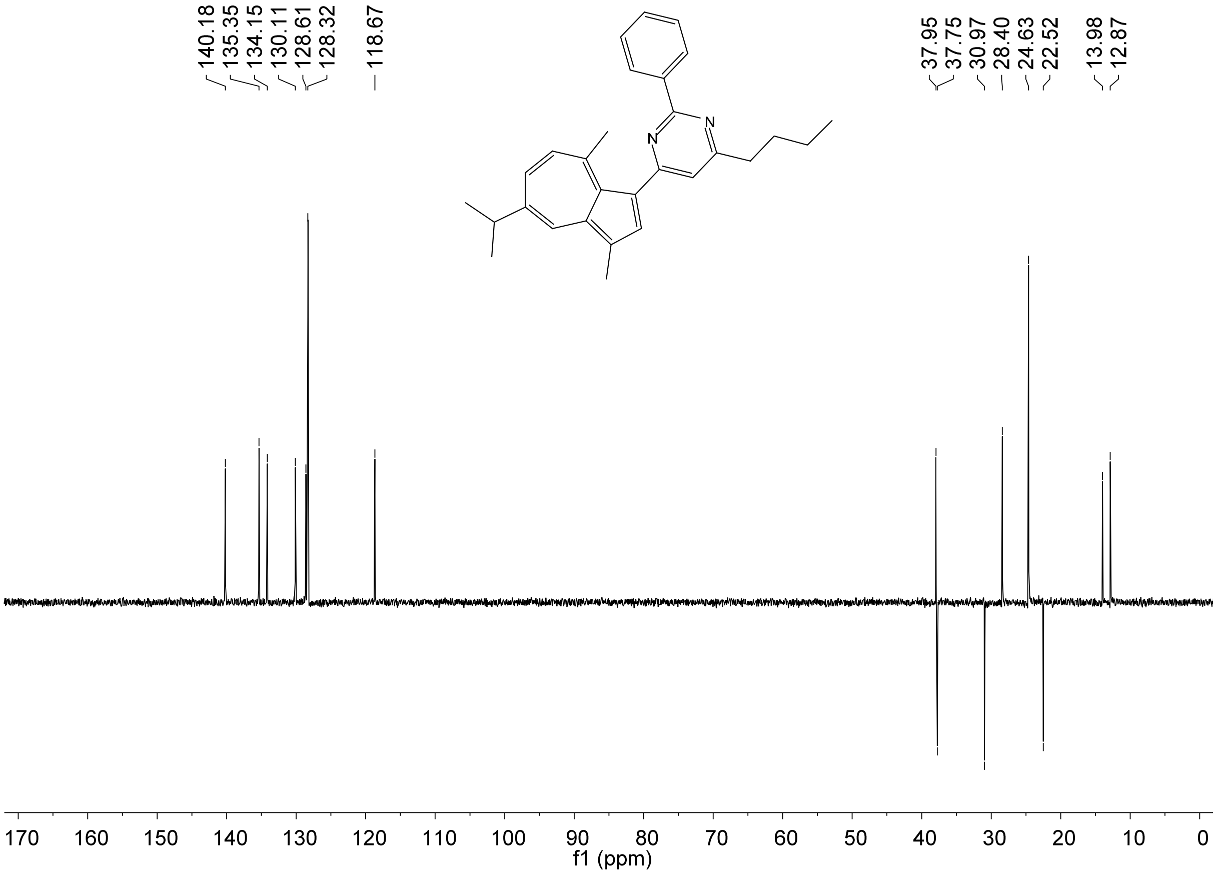

^{13}C DEPT 135-NMR of **5f** in $CDCl_3$ at 298 K (δ in ppm).

7 ^{1}H and ^{13}C NMR Spectra of Pyrazoles 7

7.1 3-(Azulen-1-yl)-1-methyl-5-phenyl-1*H*-pyrazole (7a)

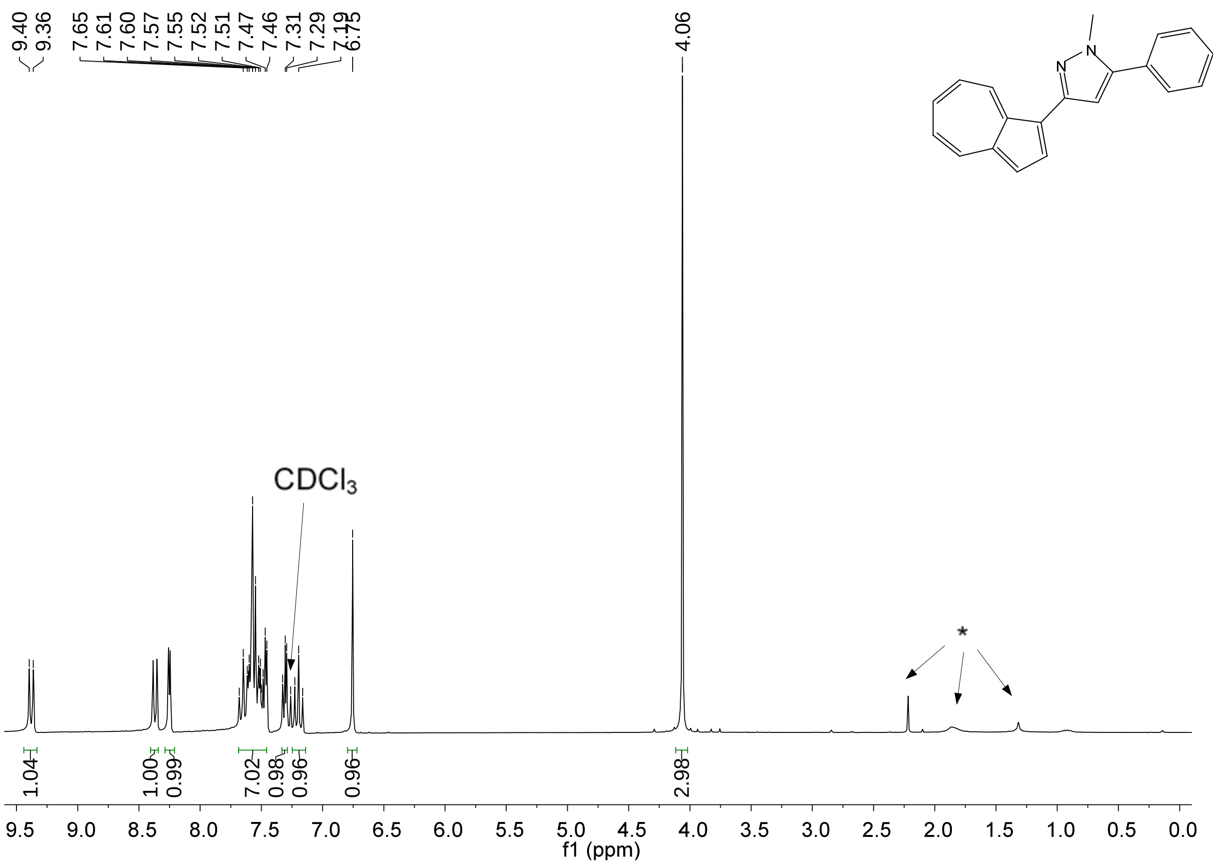

^{1}H NMR of **7a** in $CDCl_3$ at 298 K (δ in ppm). *Impurities from residual solvents.

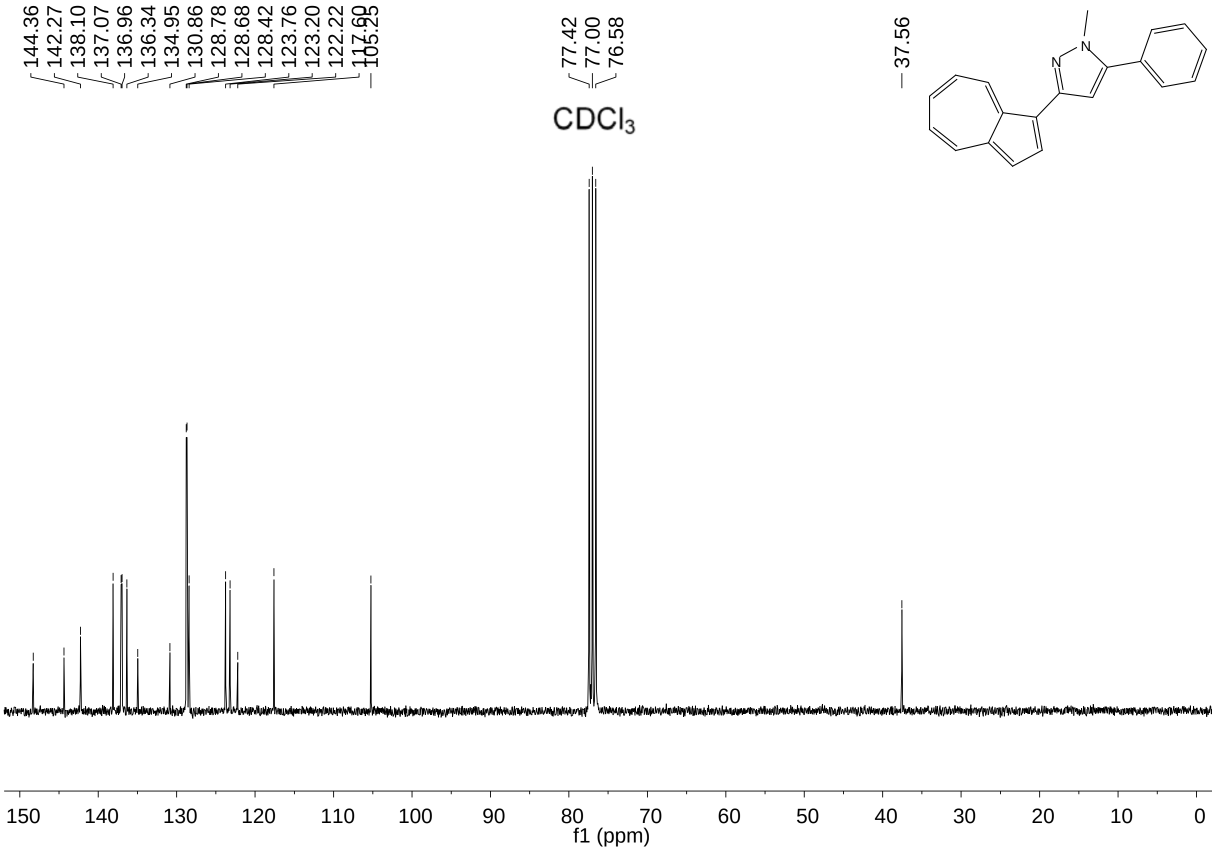

^{13}C NMR of **7a** in $CDCl_3$ at 298 K (δ in ppm).

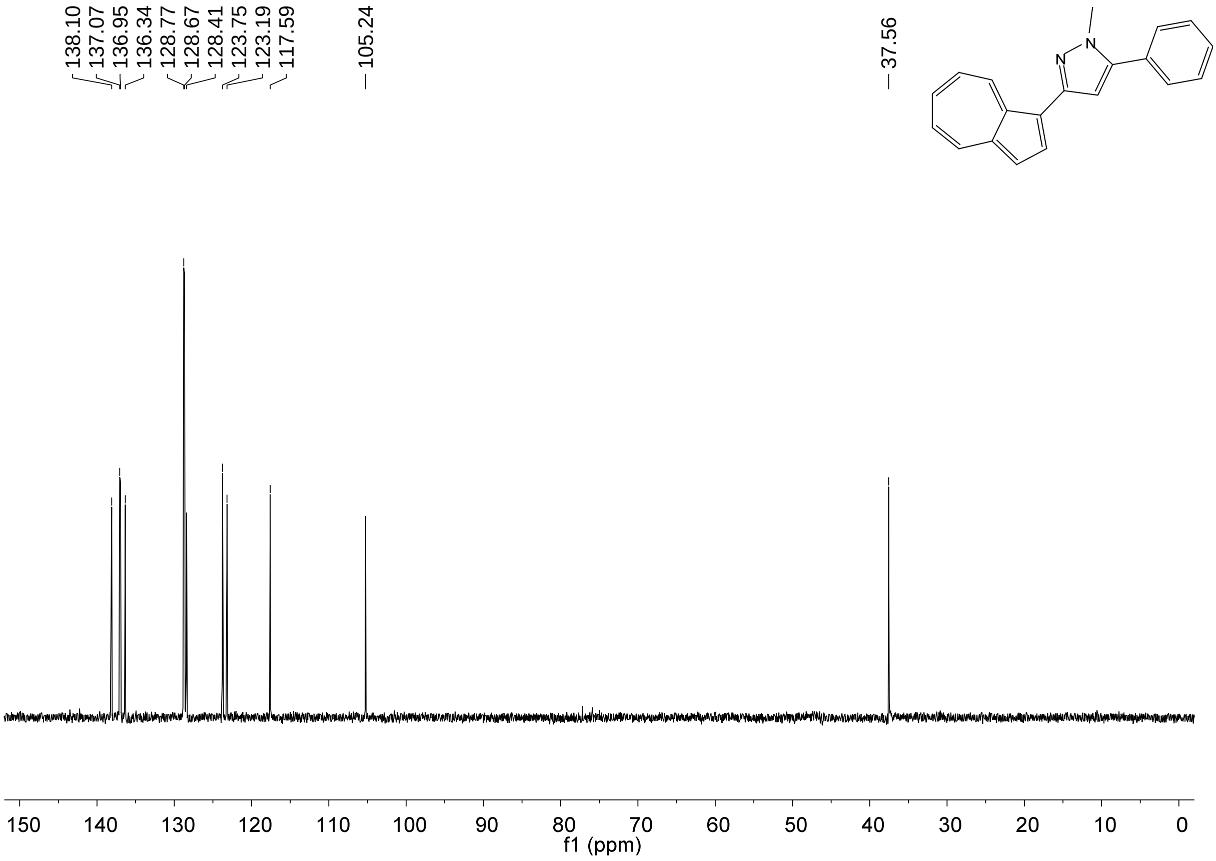

^{13}C DEPT 135-NMR of **7a** in $CDCl_3$ at 298 K (δ in ppm).

7.2 3-{[Azulen-1-yl]-1-methyl-1*H*pyrazol-4-yl}benzonitrile (7b)

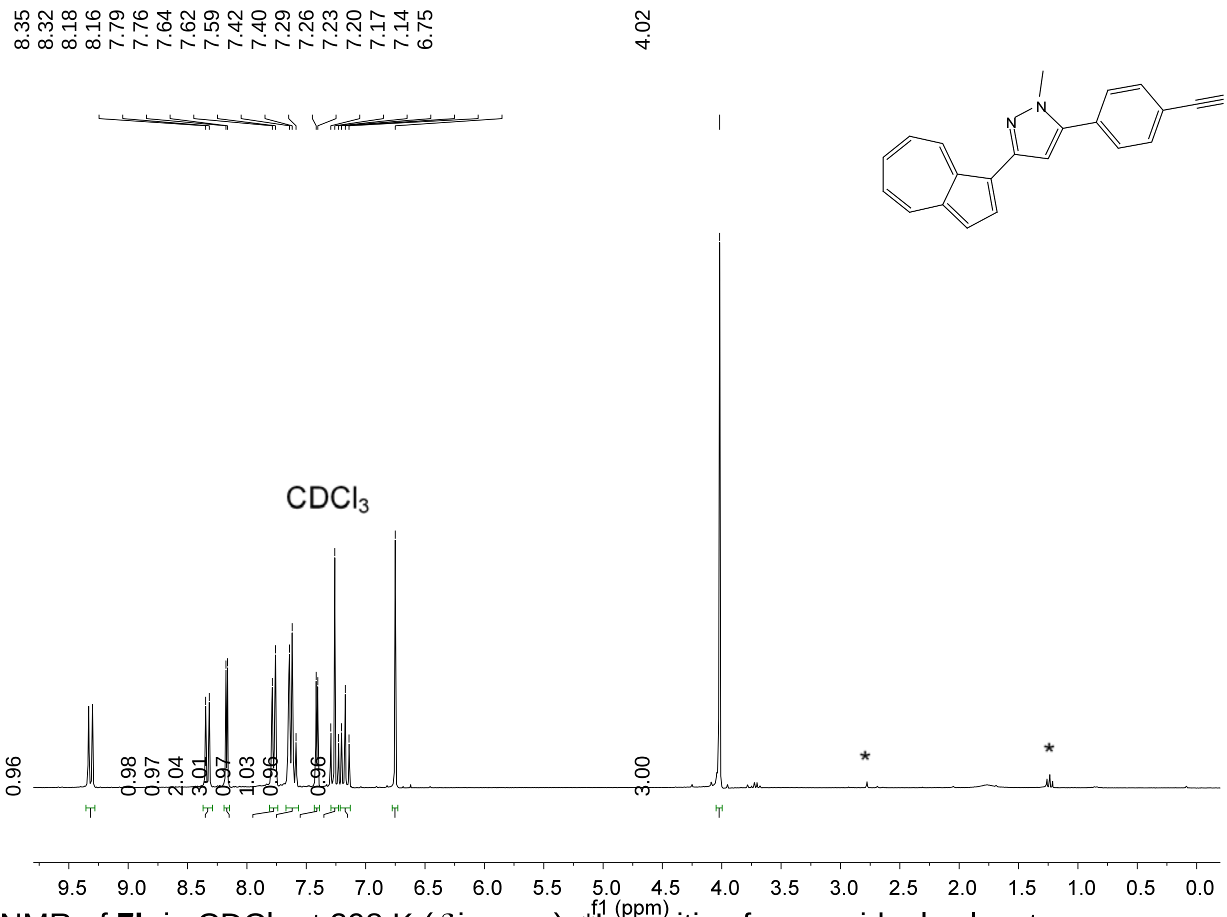

^{1}H NMR of **7b** in $CDCl_3$ at 298 K (δ in ppm). *Impurities from residual solvents.

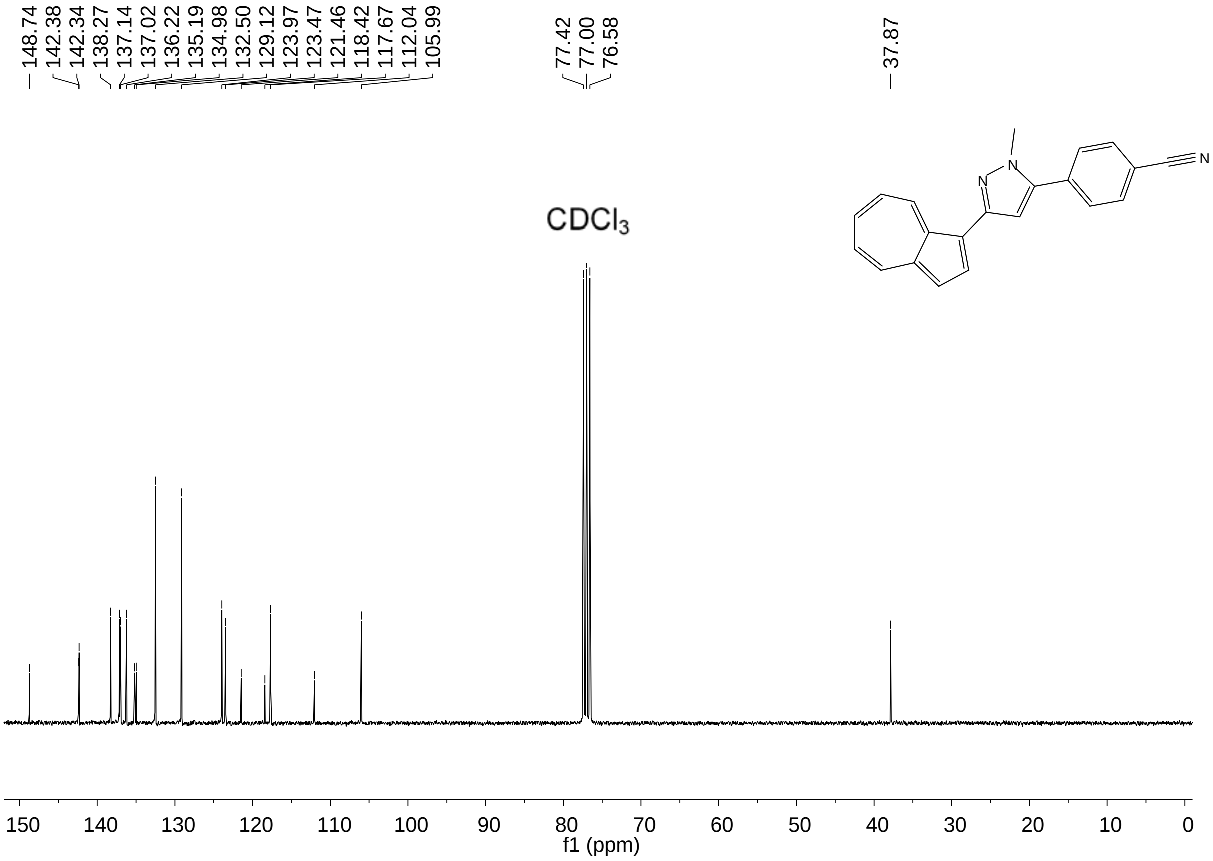

^{13}C NMR of **7b** in $CDCl_3$ at 298 K (δ in ppm).

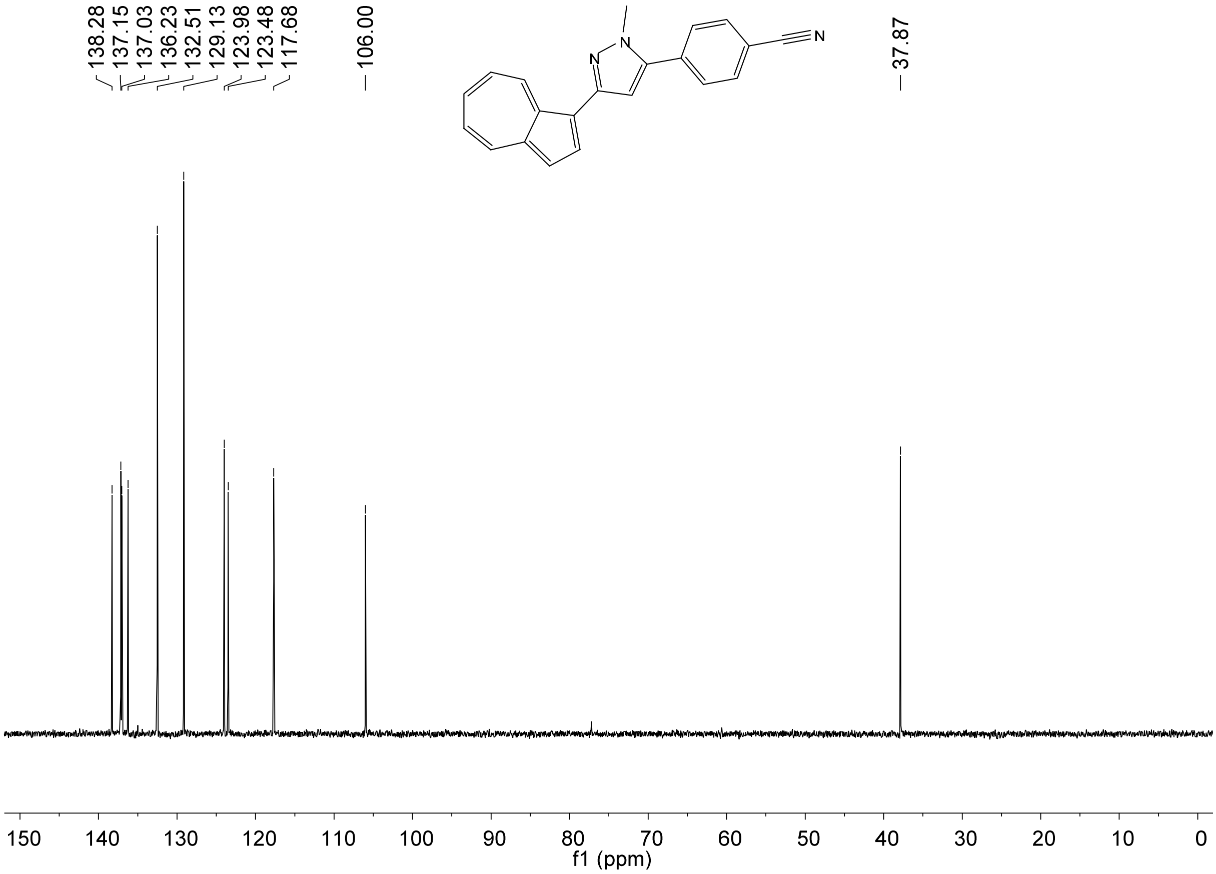

^{13}C DEPT 135-NMR of **7b** in $CDCl_3$ at 298 K (δ in ppm).
